# Genetic analyses reveal temporal stability and connectivity pattern in blue and red shrimp *Aristeus antennatus* populations

**DOI:** 10.1038/s41598-020-78634-2

**Published:** 2020-12-09

**Authors:** Melania Agulló, Sandra Heras, José-Luis García-Marín, Manuel Vera, Laia Planella, María Inés Roldán

**Affiliations:** 1 Laboratori d’Ictiologia Genètica, Universitat de Girona, c/Mª Aurèlia Capmany 40, 17003 Girona, Spain; 2 Departamento de Zooloxía, Xenética e Antropoloxía Física, Universidade de Santiago de Compostela, Campus Lugo, 27002 Lugo, Spain

**Keywords:** Evolutionary biology, Ecological genetics, Genetic markers

## Abstract

Temporal variability of the genetic structure and connectivity patterns of the blue and red shrimp *Aristeus antennatus* in the seven most important fishing grounds of the Western Mediterranean Sea, were assessed using twelve microsatellite loci during 2 consecutive years (2016 and 2017), in a total of 1403 adult individuals. A high level of geographical connectivity among groups was observed in the two studied years. In fact, no significant geographical differentiation was found in 2016 (*F*_ST_ = 0.0018, *p* > 0.05), whereas it was indicated in 2017 (*F*_ST_ = 0.0025, *p* < 0.05). This small divergence in 2017 was not attributed to the distance among locations nor to the effect of the Ibiza Channel. Significant allele frequency changes were found at local level between the 2 years (*F*_CT_ = 0.0006, *p* < 0.05), mainly due to Blanes’ fishing ground. Larval dispersal from the North to the South through the main superficial current supports the high level of connectivity pattern found. The temporal genetic instability detected in the Blanes’ fishing ground could be explained by oceanographic temporary features. Our findings evidence only one biological unit in the study region and establish the baseline for an inter-federal management plan of *A. antennatus*.

## Introduction

The blue and red shrimp *Aristeus antennatus* (Risso, 1816) has a wide distribution in the Western basin of the Mediterranean Sea^1^, where it is the most eurybathic species, with a depth range from 80 to nearly 3000 m and a peak of abundance between 400 and 800 m^2,3^. *Aristeus antennatus* mainly inhabits the muddy bottoms of the upper and middle slope where it is commercially exploited, but it is found also at the lower slope, non-exploited by fishery^4^. This species has temporal movements between the open slope and the margins of submarine canyons^3,4^. Mature males and females form mating aggregations on the middle slope during the reproductive period^4^, which takes place in late spring and summer^5^. On the other hand, even though pelagic larval duration is still unknown in *A. antennatus*, all larval stages found in a few number of studies were in the upper layers of the water column [^6^ and references therein].

In the Western Mediterranean coast, the species has been intensively exploited by trawling fishery between depths of 400–800 m^7,8^ in the Catalan-Levantine coast, the Balearic Islands and Alborán Sea (Mediterranean Subareas GSA6, GSA5 and GSA1 respectively), being considered as one of the most important marine resources^3,9^ included in the priority species list for action by General Fisheries Commission for the Mediterranean Sea^10^. Nevertheless, the lack of an integral management plan for the species along the North-western Mediterranean coast^11^ has implied that the management of *A. antennatus* has been applied locally^12,13^ without taking into account the genetic diversity and connectivity among populations and without identifying the conservation units, which should be required for a sustainable management of the species^14^.

**Figure 1:**
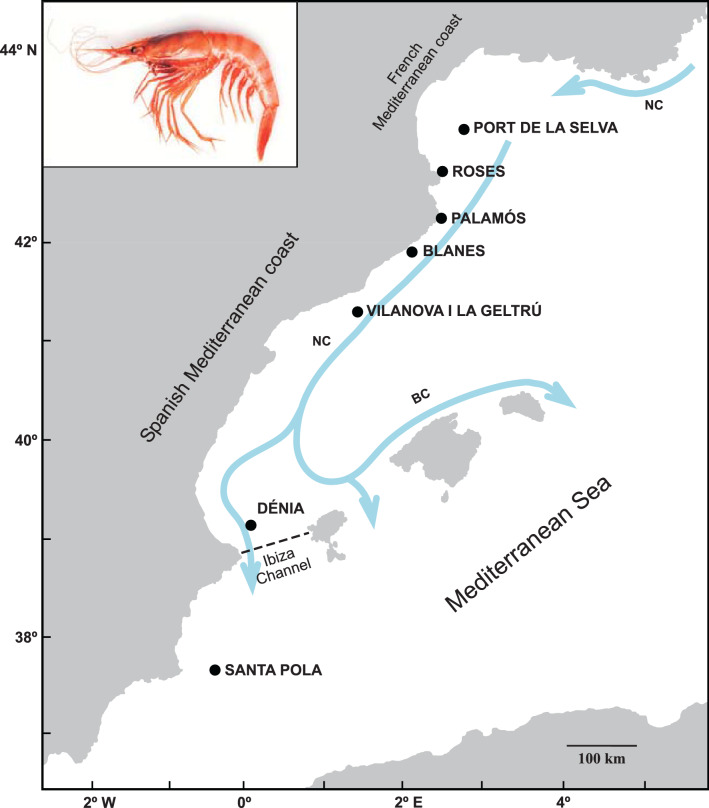
Sampling locations for *Aristeus antennatus* in the Mediterranean Sea used in this study. The dashed line represents the Ibiza Channel oceanographic barrier. Northern and Balearic currents (NC, BC) are shown as blue arrows. For further detail, see Table 1. This map was created from open IGN database (http://www.ign.es/iberpix2/visor/), using FreeHand MX 11.0 version.

Different molecular markers (allozymes, AFLP and mtDNA) have been used to identify population substructure of *A. antennatus* in the Mediterranean Sea, but they pointed out the Western Mediterranean Sea as a single genetic unit^1,15–17^. Thus, in a fine-scale study, where the differentiation could be relatively small, more highly polymorphic molecular markers are required, such as microsatellites^18^. Although a set of eight microsatellite loci failed to establish any population structure in the blue and red shrimp in the Central Mediterranean Sea^19^, microsatellites have successfully been used to identify genetic stock in other marine penaeoid species, such as *Penaeus monodon*^20^ and *Farfantepenaeus notialis*^21^. The presence of oceanographic discontinuities has been identified as one of the main factors determining the genetic differentiation among populations in marine species, but dispersal by oceanic currents promotes connectivity among them^22–24^. Along the Mediterranean coast of the Iberian Peninsula, the main surface current is the Northern Current, which flows south-westward to at least the Ibiza Channel^25^, where a part of the flow crosses this channel towards the Alborán Sea, whereas most recirculates north-eastward to the Balearic Islands, forming the Balearic current^26^. The Ibiza Channel (Fig. 1) is a passage between Ibiza island and the mainland at Cape La Nao, which due to its narrowness (80 km width and 800 m depth)^27^ has been considered an oceanographic barrier limiting the dispersion of marine species and the genetic connectivity^23,28^.

**Table 1:** Description of the analysed *A. antennatus* samples, including geographical coordinates, sampled year, depth and sample size (*N*).

Location	Sample code	Fishing ground	Geographical coordinates	Sampled year	Depth (m)	*N*
Port de la Selva	PDS16	Avión	42° 44′ 59″ N 3° 41′ 20″ E	2016	575	100
	PDS17			2017	575	98
Roses	ROS16	Cap de Creus	42° 21′ 17″ N 3° 24′ 22″ E	2016	600	100
	ROS17			2017	600	100
Palamós	PAL16	Sant Sebastià	41° 54′ 04″ N 3° 16′ 08″ E	2016	500	103
	PAL17			2017	530	100
Blanes	BLA16	La Rocassa	41° 35′ 85″ N 2° 50′ 56″ E	2016	550	100
	BLA17			2017	550	100
Vilanova i la Geltrú	VIG16	Can Pere Negre	41° 03′ 18″ N 2° 03′ 10″ E	2016	700	100
	VIG17			2017	600	100
Dénia	DEN16	Dénia’s fishing ground	39° 02′ 54″ N 0° 24′ 19″ E	2016	611	102
	DEN17			2017	630	100
Santa Pola	SPO16	Playa Nueva	37° 37′ 05″ N 0° 14′ 06″ W	2016	–	100
	SPO17			2017	550	100

Temporal changes in the distribution along the water column, in the diet and the food consumption rates, in the abundance and fishery landings of *A. antennatus* at regional scale have been reported^3,4,29,30^. It is particularly interesting to determine whether these changes are related to spatial and temporal variation on the genetic structure of *A. antennatus* populations, because a federal interest of integral management has been recently stated^31^ following the Food and Agriculture Organization recommendations^32^.

The main aim of this study was to describe the temporal stability of the genetic structure and connectivity patterns of *A. antennatus* among the most important fishing grounds in the North-western Mediterranean Sea, from the Gulf of Lions (France) to Cabo de Palos (Spain), using twelve microsatellite loci. We also analysed the influence of the Ibiza Channel as oceanographic barrier to the dispersion of individuals.

## Results

### Genetic diversity within samples

After the quality filtering, where individuals with less than eight genotyped loci were eliminated, 1386 out of 1403 individuals were used for further analysis (Table 2).

**Table 2:** Genetic diversity within samples.

Location	Sample code	n	*N*_A_	*A*_R_	*H*_O_	*H*_E_	*F*_IS_
Port de la Selva	PDS16	98	8.8	8.7	0.446	0.638	0.303
	PDS17	97	9.5	9.3	0.476	0.632	0.247
Roses	ROS16	99	8. 7	8.5	0.477	0.630	0.243
	ROS17	99	9.2	9.0	0.494	0.631	0.216
Palamós	PAL16	101	8.9	8.7	0.486	0.639	0.240
	PAL17	100	9.5	9.3	0.454	0.643	0.295
Blanes	BLA16	98	9.1	8.9	0.474	0.634	0.252
	BLA17	100	9.2	9.0	0.473	0.634	0.254
Vilanova i la Geltrú	VIG16	97	8.9	8.7	0.440	0.631	0.302
	VIG17	100	9.2	9.0	0.498	0.620	0.197
Dénia	DEN16	100	9.0	8.8	0.447	0.629	0.290
	DEN17	99	8.9	8.8	0.431	0.629	0.315
Santa Pola	SPO16	98	9.2	9.0	0.476	0.639	0.256
	SPO17	100	9.3	9.1	0.483	0.632	0.236

Number of genotyped individuals (n); mean number of alleles detected (*N*_A_); mean allelic richness (*A*_R_); mean observed heterozygosity (*H*_O_); mean expected heterozygosity (*H*_E_); inbreeding coefficient (*F*_IS_).

In all analysed samples, the twelve microsatellite loci were polymorphic, where the number of alleles per locus (*N*_A_) ranged from 2 (locus *Aa496b* in all samples, and locus *Aa751* in ROS17) to 23 (locus *Aa138* in BLA16 and locus *Aa681* in VIG17) (Supplementary Table S1) with a mean of 9.1 alleles per locus per sample. Average allelic richness (*A*_R_) ranged from 8.5 (ROS16) to 9.0 (SPO16) in 2016, and from 8.8 (DEN17) to 9.3 (PDS17 and PAL17) in 2017 (Table 2).

Observed heterozygosity (*H*_O_) ranged from 0.440 (VIG16) to 0.486 (PAL16) among samples collected in 2016, and from 0.431 (DEN17) to 0.498 (VIG17) among those in 2017. The expected heterozygosity (*H*_E_) was higher than *H*_O_ and ranged from 0.629 (DEN16) to 0.639 (PAL16 and SPO16) in 2016, and from 0.620 (VIG17) to 0.643 (PAL17) in 2017 (Table 2). Significant linkage disequilibrium was only detected in the pair of loci *Aa1255* and *Aa1061* in PAL16 after Bonferroni correction (*p* < 0.0001) but not in other locations. Therefore, all loci were included in further analyses.

Significant deviation from the Hardy Weinberg Equilibrium (HWE) occurred at 10 out of 12 loci at least in one sample, related to deficiency of heterozygote individuals (positive *F*_IS_ values, see Supplementary Table S1). Null alleles were detected in the samples with significant HWE deviations. However, estimated allele frequencies for null alleles were low, only the *Aa1255*, *Aa1444*, and *Aa818* loci had null allele frequencies higher than 0.2 in four, one and two samples, respectively (Supplementary Table S1). Nevertheless, given the lack of consistency among samples in the presence of null alleles, all loci were included in further analyses.

### Geographical and temporal variation

**Figure 2:**
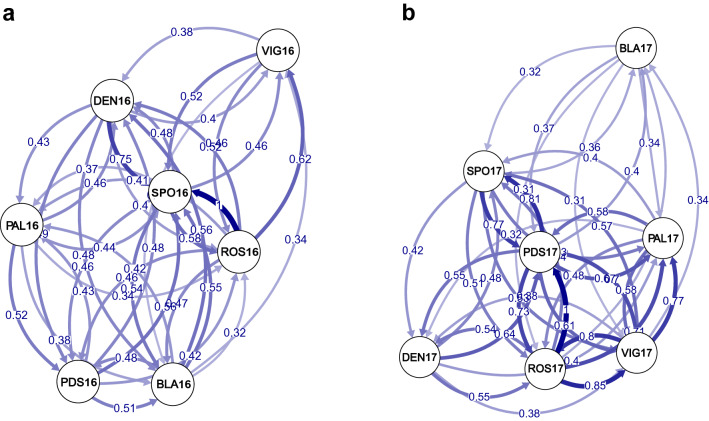
Relative migration networks estimated by divMigrate-online. Filter threshold was set to 0.3. Circles represent samples, while arrows indicate the direction and magnitude of relative migration levels using *Nm* estimator. Thicker arrows indicate stronger migration. Sample codes as in Table 1. (**a**) Illustrates 2016 collection, (**b**) illustrates 2017 collection.

There were no substantial differences in estimated *F*_ST_ values correcting, or not correcting, for the presence of null alleles. In fact, none of the pairwise *F*_ST_ comparisons between samples was significant after Bonferroni correction in year 2016. Among the 2017 samples, pairwise *F*_ST_ values including BLA17 were the highest and significant ones, although only the BLA17 and DEN17 comparison remained significant after Bonferroni correction (Table 3). Genetic and geographical distances were not correlated among 2016 samples (r = −0.403, *p* = 0.057) nor among the 2017 ones (r = 0.056, *p* = 0.581), indicating no signal of isolation by distance. For the two temporal collections, migration rates among samples suggested a high level of connectivity, but not related to the geographical proximity among locations (Fig. 2).

**Table 3:** Pairwise *F*_ST_ values between the seven samples (below the diagonal) and *p* values (above the diagonal) for the two temporal collections.

2016		PDS16	ROS16	PAL16	BLA16	VIG16	DEN16	SPO16
	PDS16	–	0.447	0.379	0.653	0.017	0.579	0.663
	ROS16	0.0014	–	0.123	0.212	0.785	0.506	0.982
	PAL16	0.0016	0.0030	–	0.166	0.006	0.297	0.167
	BLA16	0.0006	0.0024	0.0026	–	0.076	0.773	0.809
	VIG16	0.0054	0.0000	0.0062	0.0038	–	0.391	0.729
	DEN16	0.0010	0.0011	0.0020	0.0001	0.0017	–	0.913
	SPO16	0.0006	0.0000	0.0026	0.0000	0.0003	0.0000	–

2017		PDS17	ROS17	PAL17	BLA17	VIG17	DEN17	SPO17
	PDS17	–	0.985	0.600	0.113	0.874	0.688	0.926
	ROS17	0.0000	–	0.667	0.015	0.983	0.423	0.372
	PAL17	0.0008	0.0005	–	0.014	0.909	0.017	0.187
	BLA17	0.0030	0.0050	0.0052	–	0.017	**0.000**	0.031
	VIG17	0.0000	0.0000	0.0000	0.0047	–	0.068	0.622
	DEN17	0.0005	0.0014	0.0052	0.0095	0.0036	–	0.216
	SPO17	0.0000	0.0014	0.0025	0.0043	0.0005	0.0025	–

Sample codes are given in Table 1. Significance after Bonferroni correction indicated in bold (*p* < 0.0024).

Using the Evanno’s approach, the Bayesian clustering analysis revealed the most likely model to be *K* = 4 for 2016 and *K* = 3 for 2017. However, the distribution of the clusters across individuals did not show any geographical pattern in both collections (2016 and 2017) (Supplementary Fig. S1). An evaluation of the statistical power estimated a high probability (99.9–100% for chi-square and 99.6–100% for Fisher exact test) of detecting structure for a *F*_ST_ value of 0.0010–0.0025. This result suggests that our test would detect a real population structure if true estimates of *F*_ST_ were at this level. AMOVA results among the 2016 samples (unstructured 2016, Table 4) indicated that more than 99% of genetic diversity was shared among samples and only a small and not significant proportion was allocated to divergence among samples (*F*_ST_ = 0.0018, *p* > 0.05). Thus, no oceanographic groupings were tested further for 2016 collection. Otherwise, in the 2017 collection a small but significant divergence was detected among samples (*F*_ST_ = 0.0025, *p* < 0.05). Nevertheless, hierarchical analyses did not give support to the Ibiza Channel as a barrier to *A. antennatus* dispersal (2017 two regions, Table 4).

**Table 4:** Hierarchical analysis of molecular variance (AMOVA) with different grouping criteria.

Hypothesis	Source of variation	Sum of squares	Variance components	% variation	*F*-statistic	*p* value
2016 unstructured	Among samples	30.47	0.007	0.177	*F*_ST_ = 0.0018	0.270
	Within samples	5115.67	3.793	99.823		
2017 unstructured	Among samples	33.34	0.009	0.249	*F*_ST_ = 0.0025	**0.035**
	Within samples	5138.82	3.782	99.751		
**I—Oceanographic groupings**						
2017 Two regions^a^	Between regions	7.17	0.003	0.090	*F*_CT_ = 0.0009	0.135
(PDS17, ROS17, PAL17, BLA17, VIG17) (DEN17, SPO17)	Among samples within regions	26.18	0.008	0.206	*F*_SC_ = 0.0021	0.134
	Within samples	5138.82	3.782	99.704	*F*_ST_ = 0.0030	**0.035**
2017 Two regions^b^	Between regions	4.92	− 0.003	− 0.066	*F*_CT_ = 0.0000	0.514
(PDS17, ROS17, PAL17, BLA17, VIG17, DEN17) (SPO17)	Among samples within regions	28.43	0.010	0.269	*F*_SC_ = 0.0027	**0.031**
	Within samples	5138.82	3.782	99.797	*F*_ST_ = 0.0020	**0.034**
**II—Temporal variation**						
2016 versus 2017	Between temporal collections	8.12	0.002	0.056	*F*_CT_ = 0.0006	**0.027**
	Among samples within temporal collections	63.81	0.008	0.211	*F*_SC_ = 0.0021	0.054
	Within samples	10,254.49	3.785	99.733	*F*_ST_ = 0.0027	**0.009**

Sample codes as in Table 1.

Hypothesis I variation within and among different oceanographic groupings and hypothesis II between temporal collections. In bold, significant *p* values (< 0.05).

^a^Dénia included in the southward region of the Ibiza Channel.

^b^Dénia included in the northward region of the Ibiza Channel.

The AMOVA analysis for temporal variation (Table 4) assigned a significant percentage of variation to the temporal component (*F*_CT_ = 0.0006, *p* < 0.05). However, at the local level, significant differentiation between 2016 and 2017 was restricted to the Blanes’ fishing ground (*F*_ST_ = 0.0048, *p* < 0.05) (Supplementary Table S2).

## Discussion

Our mean observed and expected heterozygosities (*H*_O_ = 0.468, *H*_E_ = 0.633) were in accordance with recent studies using the same set of microsatellite loci (*H*_O_ = 0.443, *H*_E_ = 0.611 recalculated excluding *Aa421*^33^; *H*_O_ = 0.458, *H*_E_ = 0.628^34^). The mean number of alleles per locus and sample (*N*_A_ = 9.1, Table 2) was slightly higher than reported previously with the same microsatellite loci (*N*_A_ = 7.9 recalculated excluding *Aa421*^33^; *N*_A_ = 7.8^34^), probably due to the sample size in our study almost doubling the one in those previous studies. Higher diversity levels were observed in Italian and Algerian *A. antennatus* samples using eight distinct loci (*H*_O_ = 0.650, *H*_E_ = 0.800^19^). Nevertheless, our results were similar to those reported in other shrimp species, such as *Acanthephyra pelagica* from the Atlantic coast of Canada (*N*_A_ = 9.3, *H*_O_ = 0.473, *H*_E_ = 0.562^35^), the deep-sea shrimp *Rimicaris hybisae* from the Caribbean Sea (*N*_A_ = 8.2, *H*_O_ = 0.525, *H*_E_ = 0.582^36^) and *Melicertus kerathurus* from the Mediterranean Sea and the North-eastern Atlantic Ocean (*N*_A_ = 16.0, *H*_O_ = 0.455, *H*_E_ = 0.572^37^).

A common feature of studies on penaeids is a significant deficit of observed heterozygosity^38^. In the present study the fourteen samples showed a general heterozygote deficit, resulting in the high positive *F*_IS_ values (Table 2). There are some factors that could be responsible for these deviations from HWE genotypic proportions, such as inbreeding, null alleles or subpopulation structure. According to a recent study about the mating structure of *A. antennatus*, most of the mating individuals of the spawning groups are not related^33^, so we did not consider inbreeding to be the cause of the HWE disequilibrium found in our results. Null alleles could explain the heterozygote deficiency, according to our results showed by Micro-checker (Supplementary Table S1). The Wahlund effect cannot be ruled out as one reason for heterozygote deficiency, due to the possibility of having sampled genetically differentiated subpopulations or cohorts simultaneously, as it was pointed out previously for *A. antennatus*^33^. Overall, our results from pairwise *F*_ST_, Isolation by distance, relative migration networks, Bayesian analysis and AMOVAs, showed a high connectivity pattern of *A. antennatus* fishing grounds (Tables 3, 4, Fig. 2 and Supplementary Fig. S1). A result in accordance with the genetic homogeneity previously reported among the Western Mediterranean Basin red and blue shrimp populations from allozyme^15^, mitochondrial (16S rDNA, COI and control region^1,16,39^) and microsatellites diversity^19^. In a recent study using the same set of microsatellite loci, genetic differentiation between the Alborán Sea and the remainder Western Mediterranean Basin was detected^34^. However, their results did not point out any significant differentiation among the only three samples of the Spanish Mediterranean coast, whereas our results indicated some degree of differentiation in the 2017 temporal collection (Tables 3, 4). The pelagic life stages are considered the main dispersal mechanism in many marine species including crustaceans^40,41^. To date, the pelagic larval duration of *A. antennatus* is still unknown, but extrapolating from other closely related species such as *Aristaeomorpha foliacea* whose pelagic larval duration could last between 3 and 6 weeks^42^, *A. antennatus* may have a long pelagic larval duration, and therefore, high dispersal potential^41^. The larvae dispersal could be mainly driven by the Northern Current, which flows throughout the North-western Mediterranean coast (Fig. 1) from the Ligurian Sea and continues south of the Ibiza Channel. The Ibiza Channel is considered a seasonal oceanographic barrier because it is thought to have more force in reducing gene flow between populations during spring and early summer^25,27^. The seasonality is due to the presence of the Western Intermediate Water, which forms an anticyclonic gyre in the northern part of the Ibiza Channel. The gyre reduces or even blocks the southward progression of the Northern Current through this channel, and instead enhances the north-eastward recirculation to the Balearic current^25,27^. Hence, the Ibiza Channel may restrict the southward dispersal of those species with reproductive time and larval pelagic stages during that period, as it has been observed for the comber fish *Serranus cabrilla*^23^, the red gorgonian *Paramuricea clavata*^43^ and the swimming crab *Liocarcinus depurator*^28^. Instead, for species whose reproductive period is during late summer such as the dusky grouper fish, *Epinephelus marginatus*, the Ibiza Channel does not seem to prevent gene flow^44^. Usually, the reproduction of *A. antennatus* takes place from May until October, being maximum from June to September^5^, which means that during the last months of the *A. antennatus* reproductive season, the Ibiza Channel is not acting as a barrier preventing the southward larval transport.

Several factors may explain the temporal genetic instability detected in Blanes’ fishing ground between 2016 and 2017 collections (Table 4 and Supplementary Table S2). One feasible reason could be a variation in the age composition of the samples between years. *Aristeus antennatus*’ age is inferred by the cephalothorax length (CL), where each modal size class is related to an age class^7,45^. Our samples are mainly composed of 2-year-old individuals as showed by their mean CL (Supplementary Fig. S2). Besides, unpaired two-sided Student’s *t*-tests performed at each location to compare the individuals’ CL indicated a widespread variation between temporal collections (Supplementary Table S3), where the CL’s variation found in Palamós’ fishing ground had a similar pattern to the variation in Blanes, however in Palamós the results indicated genetic stability. Therefore, the temporal variation found in Blanes is most likely due to a hydro-biological matter.

Special attention should be given to the role of submarine canyons, taking into account the fact that they are considered to serve as nursery, refuge and recruitment grounds for *A. antennatus*^46,47^. Furthermore, it has been indicated that submarine canyons provide habitat heterogeneity because hydrodynamics differs between them^47,48^, so Blanes canyon may have specific physical features which combined with micro-oceanographic characteristics may causing a temporal decrease in gene flow with the surrounding areas. In fact, unlike other nearby submarine canyons such as La Fonera canyon (Palamós) and Cap de Creus canyon (Roses) where water flow has cyclonic vorticity, in Blanes canyon an anticyclonic eddy has been detected, which can cause a northward direction against the main southward water circulation^49,50^. Indeed, Clavel-Henry et al.^48^ applying different biophysical models in order to estimate potential connections between North-western Mediterranean fishing grounds of *A. antennatus*, confirmed that singular features in submarine canyons could have an important role in the species dispersal. For instance, in the open slope at the south of Blanes canyon a northward circulation of particles was detected due to the anticyclonic eddy in Blanes area^48^. Another explanation could be the cascading event, an intense dense shelf water mass formed in the Gulf of Lions and the subsequent downslope cascade, which occurs periodically during dry and cold winters^30^. Cascading events have been related to an increase in *A. antennatus* recruitment inside the North-western Mediterranean submarine canyons in the following years after a cascading event^51^, including Blanes canyon^52^. Nevertheless, this could be ruled out not only because the last cascading event (2012–2013) was some years before our collections^53,54^, but also because it did not have a significant effect in the Blanes canyon^55^. Additionally, the anthropogenic effects on submarine canyons have been deeply discussed. The bottom trawling activities have a direct effect on the ecological dynamics of the submarine canyons, causing physical disturbances which are variable over time and changes in density and composition of different meiofaunal species, as noticed in Blanes canyon^47,55^.

In conclusion, despite the temporal differentiation found in Blanes’ fishing ground, in the present study we identified a high level of genetic connectivity of *A. antennatus* from the Gulf of Lions (France) to Cabo de Palos (Spain). From a fisheries perspective, we advise considering *A. antennatus* in the North-western Mediterranean coast as one biological unit for stock assessment, and promoting coordinated fishery management plans. Taking the genetic information into account for future management advice would be of significant help to design an effective inter-federal management policy, and therefore a long term sustainability of the fishery resource would be ensured^14,22^.

## Material and methods

### Sampling collection and DNA extraction

Sampling was coordinated by the CONECTA-GEN project (Roldán 2014). Locations were chosen because of the large quantity of historical *A. antennatus* fisheries information and the interest of the Spanish Government to start an integrated management plan^11–13,31^. A total of 1403 adults were collected at seven Mediterranean locations (fishing grounds): Port de la Selva, Roses, Palamós, Blanes, Vilanova i la Geltrú, Dénia, and Santa Pola (Table 1). A sample of 100 adult individuals (~ 50♀: 50♂) was obtained from local fishermen at each location during winter season in 2016 and 2017 years (Table 1; Fig. 1). A portion of about 10 mg of muscle tissue from each individual was preserved in 70% ethanol until DNA extraction was performed using the modified phenol–chloroform protocol outlined in Fernández et al.^56^.

### Molecular markers

A set of twelve polymorphic microsatellite loci split into three multiplex PCRs and one singleplex PCR were used: *Aa138*, *Aa1255*, *Aa956*, *Aa496b*, *Aa123*, *Aa681*, *Aa667*, *Aa1444*, *Aa751*, *Aa818*, *Aa1061*, and *Aa1195*^33,57^. Microsatellite loci were amplified using approximately 30 ng of template DNA in 10 μl final volume, following the methodology described in Planella et al.^33^. PCR products were analysed in an ABI PRISM 3730xl automatic sequencer (Applied Biosystems, Foster City, CA) at the sequencing unit of the University of Santiago de Compostela (Campus Lugo). Allele scoring was performed with GeneMapper 4.0 software (Applied Biosystems, Foster City, CA), using the LIZ 500 (Applied Biosystems) as size standard.

### Genetic analysis

Genetic diversity within each sample was described by the mean number of alleles per locus (*N*_A_) and the allelic richness (*A*_R_) calculated using FSTAT v. 2.9.3^58^, and the average among loci of the observed and expected heterozygosity (*H*_O_, *H*_E_) using Genepop v. 4.6^59^. Deviations of observed genotype proportions from expected under HWE were summarized using the inbreeding coefficient *F*_IS_^60^ and its significance was statistically checked using the exact probability test of Guo and Thompson^61^ implemented in Genepop v. 4.6^59^. Genotypic linkage disequilibrium between all pairs of loci was tested with Genepop v. 4.6^59^, using a Fisher’s exact test with 10,000 dememorizations and 5000 iterations per batch. The Bonferroni correction was used to adjust significance levels for multiple simultaneous comparisons. The presence of null alleles was assessed with the software Micro-Checker v. 2.2.3^62^ by performing 1000 randomizations.

Pairwise genetic differentiation between samples (*F*_ST_) were calculated using the software Arlequin v. 3.5^63^ with 10,000 permutations to test significance. We applied the Bonferroni correction for multiple comparisons. *F*_ST_ values corrected for the presence of null alleles and its significance were estimated using the FreeNA software^64^. Hierarchical analyses of molecular variance (AMOVA^65^) as implemented in Arlequin v.3.5^63^ were performed to assess oceanographic and temporal divergence among samples. The oceanographic grouping considers the Ibiza Channel as an oceanographic barrier isolating fishing areas located northward and southward (Fig. 1). As the Dénia’s fishing ground is the closest location to the Ibiza Channel, we performed two hierarchical oceanographic groupings, the first one included Dénia in the southward region of the channel, and the second grouping included Dénia in the northward region. The hierarchical temporal grouping was used to check whether geographical structure was maintained over time. The significance of the tests was assessed by permutation with 10,000 replicates. A Bayesian clustering analysis was performed applying the admixture model implemented in STRUCTURE v. 2.3.4^66^ to infer the underlying population structure. Ten independent runs were made for each number of genetic clusters (*K* = 1–8) where each run consisted of a burn-in period length of 50,000 iterations followed by 200,000 Markov chain Monte Carlo iterations. The most likely number of *K* was selected according to the Evanno’s method^67^ implemented in STRUCTURE HARVESTER^68^.

The software POWSIM v. 4.1^69^ was used to assess the statistical power of our set of loci to detect significant genetic differentiation for the present sample sizes, number of loci and their allele frequencies, by both Pearson’s chi-square and Fisher exact tests (1000 dememorizations, 100 batches, and 1000 iterations) and 1000 replicates. We explored a range of different values for *F*_ST_ by varying the number of generation of drift (*t*). The percentage of significant outcomes was interpreted as the power of the tests for detecting the defined level of genetic divergence. The effective population size (*Ne*) was estimated for each sample using the bias-corrected version of the method based on linkage disequilibrium^70,71^ as implemented in *Ne*Estimator v. 2.1^72^. Ten out of the fourteen samples had an infinite *Ne*, so the selected *Ne* to assess the statistical power of our loci to detect genetic differentiation was 10,000.

Isolation by distance (IBD) among fishing grounds was assessed from the correlation between genetic and geographical distance matrices among studied locations. Pairwise genetic differentiation was linearized as *F*_ST_/(1 − *F*_ST_)^73^. The geographical distances were measured as the shortest coastline between locations in kilometres. The statistical significance was assessed by Mantel tests with 10,000 permutations using the software NTSYSpc v. 2.1^74^.

Using divMigrate-online^75^, the relative direction and magnitude of migration between samples for each temporal collection were summarized as a network from the estimated effective number of migrants (*Nm*).

## Supplementary information

Supplementary Information.

## Data Availability

All data are included in the article.
