# Supplementary material for: Genetic analyses reveal temporal stability and connectivity pattern in blue and red shrimp Aristeus antennatus populations
Source: Sci Rep. 2020 Dec 9;10:21505. doi: 10.1038/s41598-020-78634-2 (PMC7725768; doi:10.1038/s41598-020-78634-2)
Supplement: Supplementary file 1 — Supplementary Information. [file 41598_2020_78634_MOESM1_ESM.pdf]

Supplementary information for

Genetic analyses reveal temporal stability and connectivity pattern in blue and red shrimp *Aristeus antennatus* populations.

Melania Agulló[1], Sandra Heras[1*], José-Luis García-Marín[1], Manuel Vera[2], Laia Planella[1], and María Inés Roldán[1]

[1]*Laboratori d'Ictiologia Genètica, Universitat de Girona, c/ Mª Aurèlia Capmany 40, E-17003 Girona, Spain.*

[2]*Departamento de Zooloxía, Xenética e Antropoloxía Física, Campus Lugo, Universidade de Santiago de Compostela, E-27002 Lugo, Spain.*

*Corresponding author: sandra.heras@udg.edu

This pdf file includes:

Supplementary Figure S1

Supplementary Figure S2

Supplementary Table S1

Supplementary Table S2

Supplementary Table S3

Supplementary Table S4

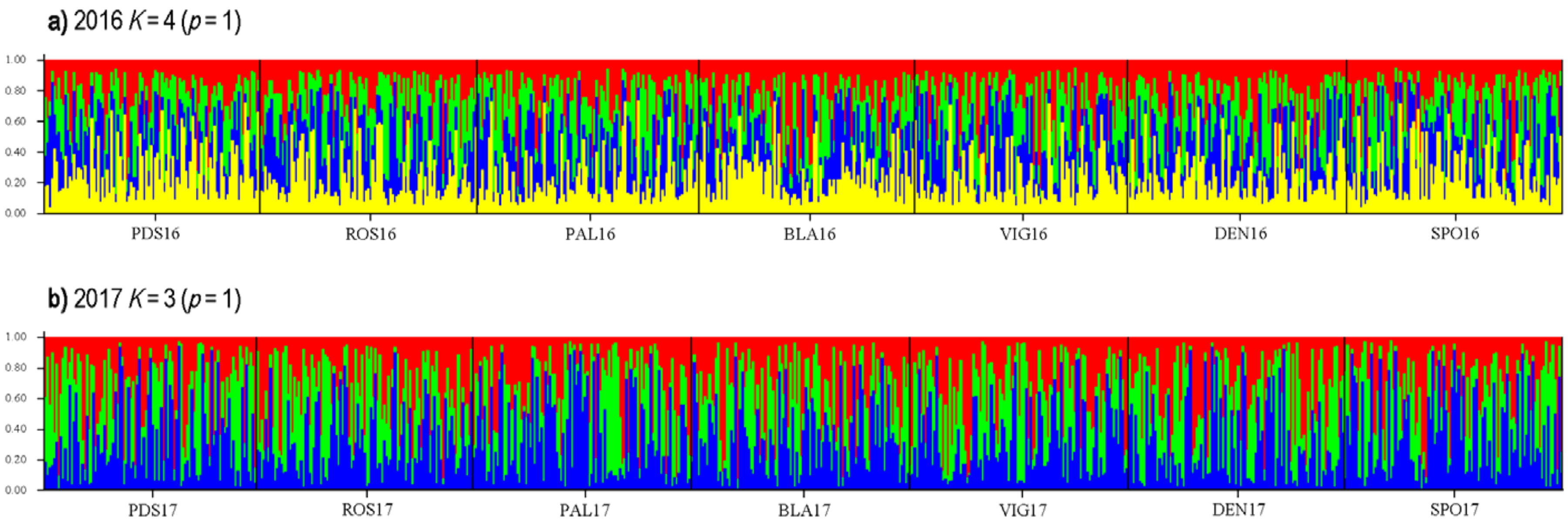

Supplementary Figure S1. Bayesian analyses of population structure. Each vertical bar represents an individual and its associated probability of belonging to each of the clusters detected (represented by colours). *K* is the most likely number of genetic clusters following the Evanno's method. Sample codes as in Table 1.

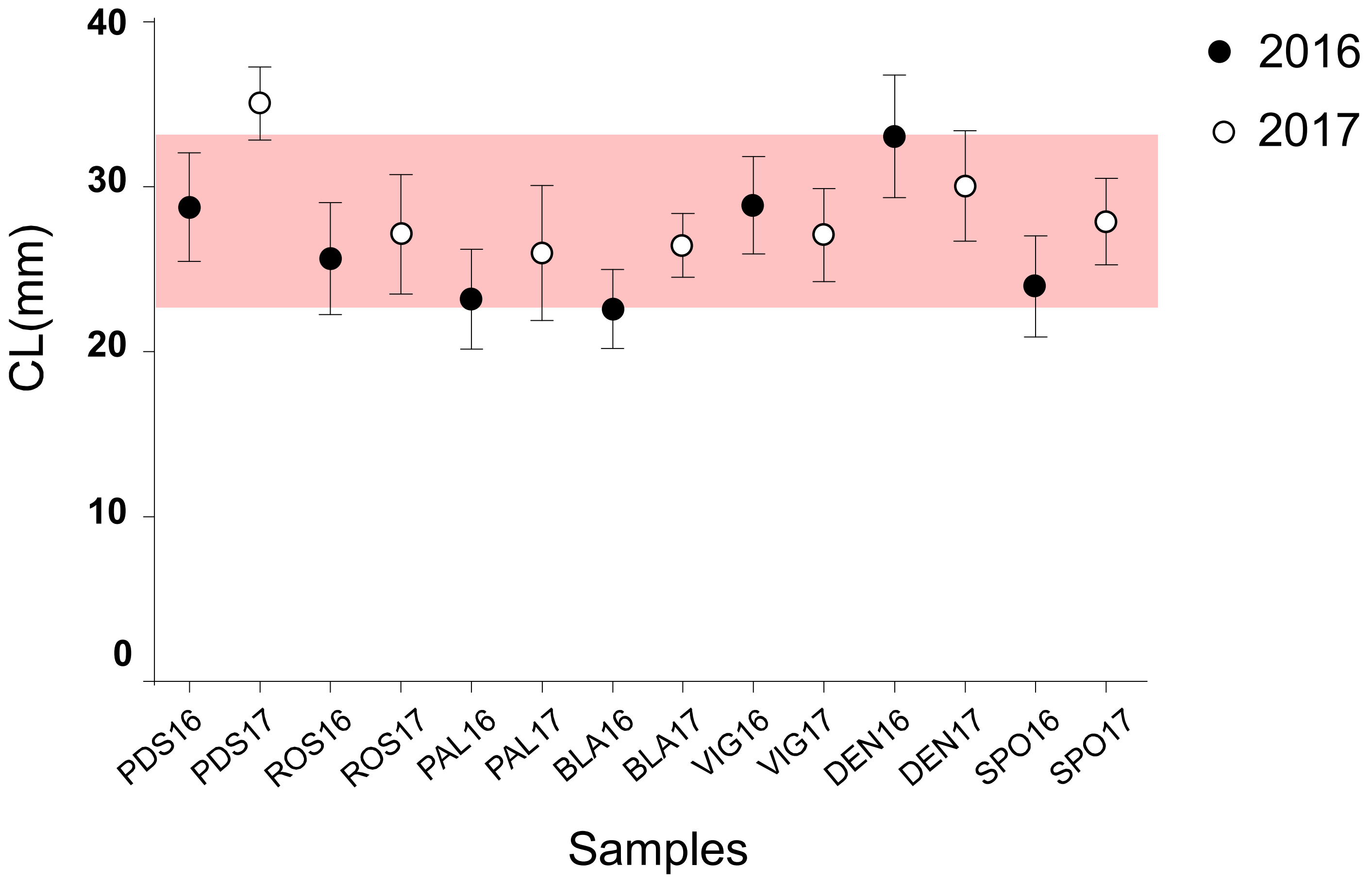

Supplementary Figure S2. Mean cephalothorax length (CL) per sample, with standard deviation. In colour, size's range for the 2nd age class (see text for details). Sample codes as in Table 1.

Supplementary Table S1. Summary statistics of genetic diversity for each of twelve microsatellite loci and fourteen samples.

Number of genotyped individuals (n); number of alleles detected per locus (N_A); observed heterozygosity (H_O); expected heterozygosity (H_E); inbreeding coefficient (F_{IS}) (* Significant deviation from Hardy-Weinberg equilibrium after Bonferroni correction (α/168, p <0.0003)); Null allele frequency (Nu).

Sample		*Aa123*	*Aa1255*	*Aa138*	*Aa1444*	*Aa496b*	*Aa667*	*Aa681*	*Aa751*	*Aa956*	*Aa1061*	*Aa1195*	*Aa818*	Mean
PDS16	n	98	95	98	94	96	94	97	86	96	95	95	94	
	N_A	6	13	20	11	2	7	19	3	6	7	5	6	8.8
	H_O	0.439	0.516	0.786	0.500	0.146	0.436	0.546	0.058	0.469	0.474	0.579	0.340	0.445
	H_E	0.626	0.791	0.903	0.813	0.154	0.692	0.787	0.287	0.594	0.706	0.612	0.653	0.638
	F_{IS}	0.299*	0.348*	0.130	0.385*	0.051	0.370*	0.305*	0.797*	0.211*	0.330*	0.054	0.478*	
	Nu	0.114	0.136	0.055	0.158		0.129	0.125	0.161	0.086	0.131		0.211	
PDS17	n	97	93	95	94	97	94	96	91	96	97	97	96	
	N_A	5	16	22	13	2	9	20	3	6	8	4	6	9.5
	H_O	0.485	0.462	0.884	0.489	0.124	0.489	0.573	0.165	0.552	0.567	0.557	0.354	0.476
	H_E	0.613	0.779	0.910	0.778	0.117	0.716	0.758	0.350	0.627	0.721	0.584	0.630	0.632
	F_{IS}	0.209*	0.406*	0.029	0.371*	-0.061	0.317*	0.244	0.529*	0.119	0.214*	0.045	0.438*	
	Nu	0.070	0.170		0.162		0.125	0.103	0.154	0.054	0.084		0.159	
ROS16	n	99	92	99	96	97	98	99	92	93	99	99	99	
	N_A	5	14	21	10	2	7	19	3	5	8	5	5	8.7
	H_O	0.434	0.478	0.788	0.500	0.144	0.459	0.546	0.163	0.613	0.444	0.717	0.414	0.477
	H_E	0.608	0.752	0.899	0.724	0.135	0.685	0.789	0.316	0.681	0.678	0.608	0.667	0.630
	F_{IS}	0.286*	0.364*	0.124*	0.310*	-0.073	0.330*	0.309*	0.484*	0.100	0.345*	-0.180	0.379*	
	Nu	0.116	0.131	0.053	0.128		0.121	0.128	0.100		0.107		0.140	

Sample		*Aa123*	*Aa1255*	*Aa138*	*Aa1444*	*Aa496b*	*Aa667*	*Aa681*	*Aa751*	*Aa956*	*Aa1061*	*Aa1195*	*Aa818*	Mean
ROS17	n	99	93	99	93	97	99	99	92	99	99	99	99	
	N_A	5	16	22	11	2	6	21	2	6	8	6	5	9.2
	H_O	0.414	0.495	0.889	0.602	0.155	0.535	0.576	0.174	0.566	0.475	0.667	0.364	0.494
	H_E	0.584	0.783	0.914	0.779	0.143	0.675	0.787	0.275	0.662	0.714	0.613	0.621	0.631
	F_{IS}	0.291	0.368*	0.027	0.227	-0.079	0.207	0.269*	0.368	0.145	0.335*	-0.089	0.415*	
	Nu	0.113	0.154		0.097		0.113	0.115	0.087		0.121		0.160	
PAL16	n	101	100	101	99	97	96	101	94	101	100	100	100	
	N_A	6	15	19	10	2	7	21	3	6	8	4	6	8.9
	H_O	0.485	0.390	0.861	0.515	0.289	0.469	0.584	0.181	0.604	0.450	0.630	0.340	0.486
	H_E	0.621	0.747	0.898	0.759	0.248	0.721	0.838	0.251	0.624	0.712	0.611	0.609	0.639
	F_{IS}	0.219	0.478*	0.041	0.321*	-0.164	0.350*	0.303*	0.278	0.032	0.368*	-0.031	0.442*	
	Nu	0.091	0.215		0.148		0.144	0.141	0.080		0.134		0.170	
PAL17	n	100	93	100	95	100	97	99	96	100	100	100	99	
	N_A	6	14	21	14	2	8	20	3	6	9	5	6	9.5
	H_O	0.510	0.376	0.800	0.526	0.140	0.516	0.515	0.094	0.580	0.510	0.510	0.343	0.453
	H_E	0.642	0.811	0.895	0.758	0.197	0.691	0.854	0.251	0.677	0.702	0.558	0.683	0.643
	F_{IS}	0.205	0.536*	0.106	0.305*	0.290	0.254*	0.397*	0.627*	0.143	0.274*	0.085	0.497*	
	Nu	0.075	0.266	0.061	0.120		0.103	0.189	0.139	0.065	0.087		0.203	
BLA16	n	98	96	96	93	96	94	97	94	94	98	98	98	
	N_A	5	16	23	11	2	8	18	3	6	8	3	6	9.1
	H_O	0.408	0.438	0.750	0.538	0.104	0.521	0.650	0.138	0.543	0.500	0.684	0.408	0.474
	H_E	0.619	0.765	0.912	0.808	0.099	0.697	0.796	0.239	0.669	0.713	0.607	0.675	0.634
	F_{IS}	0.341*	0.428*	0.178	0.335*	-0.050	0.252	0.184	0.421*	0.189	0.299*	-0.127	0.400*	
	Nu	0.134	0.168	0.067	0.140		0.098	0.074	0.073	0.068	0.106		0.152	

Sample		*Aa123*	*Aa1255*	*Aa138*	*Aa1444*	*Aa496b*	*Aa667*	*Aa681*	*Aa751*	*Aa956*	*Aa1061*	*Aa1195*	*Aa818*	Mean
BLA17	n	100	92	100	97	100	98	100	93	100	100	100	100	
	N_A	6	13	20	14	2	9	18	3	6	8	5	6	9.2
	H_O	0.410	0.337	0.800	0.650	0.130	0.510	0.600	0.129	0.530	0.510	0.630	0.410	0.473
	H_E	0.585	0.731	0.919	0.818	0.122	0.713	0.798	0.229	0.642	0.716	0.621	0.698	0.634
	F_{IS}	0.299*	0.539*	0.129	0.206*	-0.065	0.284*	0.248*	0.437*	0.175	0.288*	-0.015	0.413*	
	Nu	0.096	0.208	0.064	0.089		0.110	0.104	0.074	0.065	0.134		0.168	
VIG16	n	95	88	93	96	95	88	96	90	91	96	97	95	
	N_A	6	13	21	10	2	7	20	3	6	7	6	6	8.9
	H_O	0.390	0.580	0.850	0.384	0.095	0.443	0.563	0.089	0.560	0.396	0.598	0.337	0.440
	H_E	0.619	0.764	0.914	0.713	0.110	0.696	0.851	0.258	0.696	0.680	0.617	0.654	0.631
	F_{IS}	0.371*	0.241*	0.070	0.460*	0.137	0.364*	0.339*	0.656*	0.195	0.418*	0.030	0.485*	
	Nu	0.154	0.121		0.179		0.135	0.181	0.162	0.089	0.187		0.182	
VIG17	n	100	85	100	98	100	96	100	96	100	99	99	99	
	N_A	4	15	21	11	2	7	23	3	6	8	4	6	9.2
	H_O	0.500	0.435	0.890	0.561	0.170	0.604	0.630	0.104	0.520	0.495	0.647	0.404	0.498
	H_E	0.592	0.739	0.906	0.753	0.156	0.692	0.839	0.189	0.628	0.719	0.586	0.655	0.620
	F_{IS}	0.155	0.411*	0.018	0.254*	-0.088	0.127	0.249*	0.449	0.171	0.311*	-0.104	0.383*	
	Nu	0.065	0.150		0.099		0.067	0.096	0.072	0.082	0.141		0.153	
DEN16	n	100	99	100	97	100	100	99	98	100	100	100	100	
	N_A	6	16	20	10	2	8	21	3	6	7	4	5	9.0
	H_O	0.370	0.384	0.800	0.412	0.110	0.580	0.566	0.184	0.610	0.400	0.620	0.320	0.447
	H_E	0.592	0.784	0.903	0.756	0.104	0.699	0.847	0.292	0.644	0.697	0.606	0.630	0.629
	F_{IS}	0.375*	0.510*	0.114	0.454*	-0.053	0.170	0.333*	0.371	0.052	0.426*	-0.023	0.492*	
	Nu	0.129	0.234	0.047	0.201		0.062	0.148	0.084		0.170		0.183	

Sample		*Aa123*	*Aa1255*	*Aa138*	*Aa1444*	*Aa496b*	*Aa667*	*Aa681*	*Aa751*	*Aa956*	*Aa1061*	*Aa1195*	*Aa818*	Mean
DEN17	n	99	93	99	96	99	98	98	87	99	99	99	99	
	N_A	4	14	22	12	2	8	19	3	6	7	5	5	8.9
	H_O	0.384	0.387	0.808	0.490	0.101	0.449	0.571	0.138	0.495	0.455	0.525	0.333	0.431
	H_E	0.584	0.830	0.918	0.772	0.096	0.685	0.744	0.336	0.626	0.714	0.583	0.644	0.629
	F_{IS}	0.343*	0.534*	0.120	0.366*	-0.048	0.345*	0.232*	0.589*	0.210	0.363*	0.099	0.482*	
	Nu	0.144	0.231	0.048	0.147		0.131	0.097	0.156	0.090	0.152	0.051	0.190	
SPO16	n	98	92	98	97	98	97	97	94	98	98	98	98	
	N_A	5	16	21	12	2	8	19	3	6	8	4	6	9.2
	H_O	0.439	0.435	0.857	0.454	0.143	0.516	0.567	0.106	0.592	0.459	0.663	0.459	0.476
	H_E	0.603	0.790	0.915	0.788	0.133	0.682	0.827	0.251	0.692	0.705	0.591	0.692	0.639
	F_{IS}	0.272	0.450*	0.063	0.424*	-0.072	0.244*	0.314*	0.576*	0.145	0.349*	-0.122	0.336*	
	Nu	0.091	0.192		0.169		0.120	0.148	0.108		0.148		0.158	
SPO17	n	100	94	100	96	100	98	100	97	100	99	100	100	
	N_A	5	14	22	14	2	9	20	3	6	8	4	5	9.3
	H_O	0.460	0.457	0.800	0.573	0.080	0.500	0.530	0.175	0.670	0.495	0.650	0.400	0.483
	H_E	0.616	0.760	0.898	0.772	0.077	0.744	0.785	0.324	0.630	0.717	0.581	0.687	0.632
	F_{IS}	0.253	0.398*	0.109	0.258*	-0.037	0.328*	0.325*	0.458*	-0.063	0.310*	-0.119	0.418*	
	Nu	0.079	0.168	0.057	0.098		0.121	0.143	0.118		0.138		0.164	

Supplementary Table S2. Pairwise F_{ST} and *p* values between temporal collections for the seven locations. Significance indicated in bold (p <0.05).

2016 vs 2017	**Port de la Selva**	**Roses**	**Palamós**	**Blanes**	**Vilanova i la Geltrú**	**Dénia**	**Santa Pola**
**F_{ST} value**	0.0000	0.0007	0.0037	0.0048	0.0021	0.0034	0.0001
p	0.958	0.574	0.065	**0.020**	0.260	0.122	0.767

Supplementary Table S3. Unpaired two-sided Student's *t*-test results performed to compare the individuals' cephalothorax length (CL) between temporal collections for the seven locations. In bold, significant *p* values at 0.05 level. Degree of freedom (df). Analysis performed in GraphPad Prism version 8.4.1 (GraphPad Software, San Diego, CA, USA).

2016 vs 2017	**Port de la Selva**	**Roses**	**Palamós**	**Blanes**	**Vilanova I la Geltrú**	**Dénia**	**Santa Pola**
p	**<0.000**	**0.000**	**<0.000**	**<0.000**	**<0.000**	**<0.000**	**<0.000**
t	15.64	2.97	5.55	12.55	4.46	6.00	9.71
df	194	198	201	198	198	200	198

Supplementary Table S4. Raw data set.

Name	POP	Aa123A	Aa123B	Aa1255A	Aa1255B	Aa138A	Aa138B	Aa1444A	Aa1444B	Aa496bA	Aa496bB	Aa667A	Aa667B	Aa681A	Aa681B	Aa751A	Aa751B	Aa956A	Aa956B	Aa1061A	Aa1061B	Aa1195A	Aa1195B	Aa818A	Aa818B
Aa1219	1	427	427	139	139	197	205	182	194	412	412	250	256	224	264	230	230	205	205	197	207	198	198	175	175
Aa1220	1	431	431	133	133	197	205	196	198	412	412			248	248	230	230	205	209	191	207	195	198	170	190
Aa1221	1	431	431	155	155	201	217	182	182	412	412	253	256	238	258	235	235	213	213	197	205	195	201	175	175
Aa1222	1	427	431	133	133	215	221	182	182	412	418	256	256	230	238	230	235	205	213	207	207	195	195	165	170
Aa1223	1	427	429	137	139	217	223	192	192	412	418	250	256	230	236	230	230	209	209	189	207	201	201	170	170
Aa1224	1	429	431	125	125	211	213	182	182	412	412	256	256	236	238	230	230	213	213	191	209	198	201	165	170
Aa1225	1	429	429	143	143	201	219	182	182	412	412	253	253	224	236	230	230			191	207	195	195	170	170
Aa1226	1	427	431	137	155	203	213	182	182	412	412	256	256	236	236	230	230	205	209	195	207	198	198	175	190
Aa1227	1	427	431	137	137	201	217	182	194	412	412	241	250	238	248	230	230	201	217	191	191	195	201	190	190
Aa1228	1	429	431	143	169	213	213	184	206	412	418	250	256	258	258	230	230	205	209	207	207	192	198	165	170
Aa1229	1	427	431	133	155	213	213	184	194	412	418	253	253	236	236	230	230	213	213	197	197	195	195	170	190
Aa1230	1	431	431	125	135	213	221	182	192	412	412	250	256	264	264	235	235	201	213	191	207	195	198	165	190
Aa1231	1	427	431	137	137	189	201			412	412	244	256	236	240	230	230	209	213	191	195	195	201	170	175
Aa1232	1	429	429	137	137	205	217	194	194	412	412	256	256	230	230	230	230	213	213						
Aa1233	1	429	431	125	137	199	213	194	196	412	418	250	256	268	268	230	230	201	213	205	207	195	195	170	170
Aa1234	1	427	431			223	227	194	194	412	412	250	253	236	236	230	230	213	213	191	195	195	195	170	170
Aa1235	1	427	431	125	125	197	217	182	182	412	418	250	256	236	236	235	235	201	213	195	207	195	201	170	170
Aa1236	1	431	431	125	125	221	227	184	194	412	412	250	253	236	266	230	230	213	213	207	207	195	195	165	175
Aa1237	1	431	431	137	137	205	205	186	194	412	412	253	253	238	268			213	213	191	191	195	198	170	175
Aa1238	1	427	427	135	137	201	217	192	192	412	412			238	238	230	235	201	209	207	207	195	195	170	175
Aa1240	1	429	431	125	165	205	217	182	194	412	412	250	256	238	264	235	235	205	209	197	207	195	201	190	190
Aa1241	1	427	427	137	137	217	231	182	208	412	412	256	256	236	248	230	230	213	213	207	207	195	195	165	165
Aa1242	1	427	435	137	139	211	211	182	182	412	418	250	256	236	236	230	230	213	213	191	191	195	195	180	180
Aa1243	1	429	431	125	141	203	213	182	194	412	412	256	256	236	236	235	235	213	213						
Aa1244	1	431	431	137	137	205	213	182	194	412	412	256	256	236	314			213	213	191	195	195	195	170	170
Aa1245	1	431	431	137	139	195	213	194	194	412	412			248	258	235	235	209	213	197	197	195	198	170	170
Aa1246	1	431	431	137	137	213	221	192	194	412	412	250	256	236	286	235	235	213	213	207	207	195	198	170	170
Aa1247	1	427	427	165	167	201	203	182	182	412	412	253	253	242	242	230	230	213	213	191	195	195	198	175	190
Aa1248	1	429	431	139	167	217	231	192	192	412	412	244	256	230	236	230	230	209	213	207	207	195	201	165	170
Aa1249	1	427	431	125	133	213	213	182	192	412	412	244	256	230	236			193	213	191	197	198	201	170	170
Aa1250	1	431	431	133	139	197	213	186	206	412	412	256	256	238	264	230	230	213	213	191	191	192	195	165	165
Aa1251	1	431	431	133	157	205	213	182	192	412	412	250	256	258	258	230	230	213	213	207	207	195	198	170	170
Aa1252	1	431	431	139	139	217	217	184	194	412	412	256	256	322	322	230	230	205	209	207	207	198	198	175	190
Aa1253	1	431	431	169	169	191	201	182	208	412	412	250	250	236	236	230	230	209	213	207	207	195	198	165	170
Aa1254	1	431	431	137	137	201	213	194	194	412	412	253	253	236	240	230	230	209	213	207	207	198	198	165	165
Aa1255	1	427	427	137	137	187	211			412	412	241	250	266	314	230	230	209	213	191	191	195	198	170	175
Aa1256	1	431	431	137	137	207	213	194	194	412	412	256	256	236	236	230	230	201	205	191	207	195	201	165	165
Aa1257	1	425	427	151	155	207	221	182	192	412	412	256	256	240	240	230	230	213	213	207	207	195	198	190	190
Aa1258	1	431	431	135	151	211	217	182	192	412	412	244	256	238	238	230	230	209	213	207	207	195	201	170	175
Aa1259	1	431	431	137	137	205	205	198	198	412	412	256	256	238	238	230	235	213	213	191	191	195	198	170	170
Aa1260	1	431	431	125	125	199	205	184	194	412	412	250	250	236	264	230	230	213	213	191	191	198	201	175	175
Aa1261	1	431	431	139	139	207	207			412	412	250	250	260	260	230	235	213	213	191	191	195	198	170	170
Aa1262	1	427	427	125	137	217	217	182	192	412	412	253	253	248	248	230	230	201	201	207	209	195	195	170	175
Aa1263	1	427	431	133	137	213	217	184	194			256	256	236	236	230	230	209	213	191	207	198	198		
Aa1264	1	429	429	125	135	197	221	206	208	412	412	253	256	236	236	235	235	213	213	191	207	195	198	170	170
Aa1265	1	429	431	133	137	187	187	182	182	412	412	250	250	238	238	230	230	213	213	207	207	195	195	190	190
Aa1266	1	427	427	137	137	221	231	182	182	412	418	256	256	236	240	230	230	209	213	191	197	195	198	165	170
Aa1267	1	431	431	125	137	221	227	182	188	412	412	250	256	236	236	230	230	213	213	191	209	195	195	170	175
Aa1268	1	429	431	125	137	191	201	186	186	412	412	250	256	240	260	230	230	209	213	189	207	198	198	170	170
Aa1269	1	429	431	125	137	213	213	206	206	412	412	241	241	236	268	230	230	213	213	207	207	195	195	170	170
Aa1270	1	429	429	137	137	201	217	184	206	412	418	256	256	236	236	230	230	205	209	191	207	195	201	170	170
Aa1271	1	429	431	137	137	223	231	194	194	412	418	250	256	236	258	230	230	213	213	191	207	195	198	170	170
Aa1272	1	431	433	125	125	213	213	182	188	412	412	253	271	236	264	230	230	213	213	191	207	195	198	170	170
Aa1273	1	427	431	135	139	219	219	186	186	412	412	256	256	236	236	230	230	213	213	195	207	195	195	170	170
Aa1274	1	431	431	125	125	213	217	184	194	412	412	250	250	236	236	230	230	213	213	191	191	198	201	175	190
Aa1275	1	427	431	139	157	201	217	194	194	412	412	241	253	236	236	230	230	205	213	195	197	195	195	165	170
Aa1276	1	431	431	137	137	187	201	186	208	412	412	256	256	238	248	230	230	213	213	207	207	189	195	165	165
Aa1277	1	431	431	137	137	201	213	182	182	412	412	241	250	236	236	230	230	213	213	195	195	198	198	170	170
Aa1278	1	431	431	125	137	213	213	206	206	412	412	256	256	232	236	230	230	205	213	195	195	195	201	170	170
Aa1279	1	431	431	137	137	217	217			412	412	256	256	236	236	235	235	205	209	197	207	195	198	190	190
Aa1280	1	431	431	125	137	207	207	192	206	412	412	250	250	236	242	230	230	209	213	195	209	198	198	165	170
Aa1281	1	427	429			213	215	194	194	412	412	250	250	236	236	235	235	213	213	197	205	195	198	170	170
Aa1282	1	429	429	165	169	197	201	194	194	412	412	250	250	236	236	230	230	193	209	195	207	195	195	165	175
Aa1283	1	427	427	139	139	207	217	192	202	412	418	250	250			230	230	201	213	191	207	195	195	170	170
Aa1284	1	427	431	125	137	187	201	182	184	412	412	250	256	266	314	230	230	209	213	191	191	195	195	170	170
Aa1285	1	427	427	137	137	201	217	182	182	412	412	253	253	228	320	230	230	213	213	207	207	198	198	175	175
Aa1286	1	429	431	125	137	211	225	184	184	412	412	250	250	236	320			213	213	191	191	195	201	170	170
Aa1287	1	427	429	137	137	201	221	186	186	412	412	250	256	232	236	230	230	193	193	191	207	195	198	175	175
Aa1288	1	427	427	143	143	201	201	186	186	412	412	250	250	238	258			213	217	191	191	195	198	170	170
Aa1289	1	427	431	137	151	205	231	192	192	412	412	253	253	236	268	230	230	205	205	191	195	198	198	175	175
Aa1290	1	431	431	137	137	201	217	182	192	412	412	250	253	230	240			201	201	207	207	195	195	170	185
Aa1291	1	427	427	125	139	217	231	182	194	412	412	250	256	236	236	230	230	201	213	195	195	195	195	170	170
Aa1292	1	429	431	125	125	201	213	182	182	412	412			236	236	230	230	213	213	191	191	195	201	175	190
Aa1293	1	429	431	137	143	207	221	182	194	412	418	250	250	236	238	225	225	201	209	207	207	195	198	185	185
Aa1294	1	425	427	137	167	205	213	184	198	412	412	253	253	240	260			205	213	195	207	195	201	170	170
Aa1295	1	427	431	137	151	211	217	184	194	412	412	256	256	236	286	230	230	209	213						
Aa1296	1	431	431	133	137	195	213	182	192	412	412	250	250	236	236	230	230	213	213	191	191	195	198	170	170

Aa1297	1	427	427	137	139	203	221	184	194	412	412	250	250	236	248	230	230	209	213	189	189	198	198	170	185
Aa1298	1	429	431	155	165	199	199	182	206			250	256	236	236			201	213	207	207	195	201	175	190
Aa1299	1	431	431	137	137	187	221	182	182	412	412	256	256	248	248	235	235	205	213	191	195	195	195	170	170
Aa1300	1	427	429	137	137	213	231	182	202	412	412	253	256	236	238	230	230	213	213	191	207	195	198	175	190
Aa1301	1	431	431	125	137	201	231	192	192	412	412	241	271	260	322			201	201	191	207	198	198	165	170
Aa1302	1	431	431	125	143	213	231	192	192	412	412	253	256	236	236			209	213	197	197	198	201	175	175
Aa1303	1	431	431			187	231	184	184	412	412	241	256	238	238	230	230	205	205	191	209	195	201	175	190
Aa1304	1	431	431	137	155	201	217	184	186	412	412	250	250	248	248	230	230	205	213	195	207	201	201	170	190
Aa1305	1	431	431	137	139	215	221	192	192	412	412	256	256	236	264	230	230	213	213	195	195	195	195	170	170
Aa1306	1	427	429	135	137	217	217	182	192	412	418	250	250	228	264	230	235	213	213	191	207	195	201	170	170
Aa1307	1	427	431	137	137	211	221	194	194	412	412	250	274	236	236	230	230	201	213	191	207	195	201	170	170
Aa1308	1	427	429	137	137	199	221	182	206	412	412	253	253	238	248	230	230	205	209	207	207	195	198	170	170
Aa1309	1	431	431	125	125	205	211	184	184	412	412	253	256	236	236	230	230	209	213	207	207	195	195	170	170
Aa1310	1	429	429	167	169	221	231	182	182	412	412	250	253	236	280	230	230	213	213	207	207	198	201	190	190
Aa1311	1	427	429	135	139	205	231	186	196	412	412	256	256	236	264	230	230	213	213	191	191	195	198	170	170
Aa1312	1	427	427	135	139	217	231	194	194	412	412	250	256	236	264	230	230	213	213	207	207	195	195	170	170
Aa1313	1	427	429	165	165	205	217	182	182	418	418	250	253	236	280	230	230	213	213	191	207	195	195	175	175
Aa1314	1	427	427	141	141	231	231	194	194	412	412	256	256	230	236	230	230	193	193	207	207	195	198	170	170
Aa1315	1	429	431	143	143	213	213	182	206	412	412	250	250	224	230	230	230			191	197	195	201	170	170
Aa1316	1	431	431	125	167	201	217	194	194	412	412	253	256	264	322			213	213	207	207	195	198	170	170
Aa1317	1	427	429	137	137	217	221	182	182	412	418	250	250	236	264			201	213	195	195	198	201	165	165
Aa1329	2	427	427	137	137	201	217	182	192	412	412	256	256	236	240	230	235	213	213	207	207	195	198	190	190
Aa1330	2	429	431	137	155	211	231	182	194	412	412	250	256	236	240	230	230	213	213	207	209	198	201	170	175
Aa1331	2	427	427	137	137	213	221	182	194	412	412	250	250	236	236	230	230	205	213	195	207	195	195	175	175
Aa1332	2	429	431	137	137	203	213	184	184	412	418	244	250	238	248	230	230	213	213	191	197	195	198	175	175
Aa1333	2	431	431	137	137	203	205	184	184	412	412	256	256	230	238			209	213	191	191	198	201	175	175
Aa1334	2	427	429	137	137	201	217	182	182	412	412	256	256	236	240			205	213	191	207	195	201	170	170
Aa1335	2	427	431	137	137	191	213	182	182	412	412	253	253	236	236	230	230	201	213	191	191	195	198	175	175
Aa1336	2	431	431	137	155	191	207	194	194	412	412	256	256	236	236	230	235	205	209	191	191	195	201	170	190
Aa1337	2	427	427	137	139	201	207	182	182	412	412	253	253	236	248	230	230	205	209	191	207	195	198	175	190
Aa1338	2	429	431	137	137	201	201	182	182	412	412	250	250	240	320	230	230	205	213	191	191	198	198	170	170
Aa1339	2	431	431	137	137	213	227	194	194	412	412	256	271	240	248	225	225	205	213	191	195	198	201	175	190
Aa1340	2	429	431	125	125	201	217	182	182	412	418	250	250	240	240	230	230	201	213	191	197	195	198	170	170
Aa1341	2	431	431	125	137	213	217	182	182	412	412	256	259	240	260	230	230	205	209	191	191	195	198	190	190
Aa1342	2	431	431	137	137	213	231	182	182	412	412	250	253	230	230	225	230	213	213	191	207	198	201	170	170
Aa1343	2	427	427	137	137	213	215	182	192	412	418	253	271	236	236	230	230	213	213	191	191	195	198	165	170
Aa1344	2	427	431	125	125	201	213	192	192	412	412	256	256	236	236	225	225	193	213	207	207	195	201	170	170
Aa1346	2	431	431	125	137	207	211	184	194	412	418	253	253	238	238	230	230	201	213	193	195	195	198	170	170
Aa1347	2	429	431	135	137	201	207	194	206	412	412	250	256	238	286	230	230	201	213	191	207	195	198	170	170
Aa1348	2	429	431	133	137	207	213	182	184	412	412	256	256	236	236	230	230	201	213	191	191	195	198	170	170
Aa1349	2	427	427	139	141	201	203	194	194	412	418	250	250	236	236	230	230	201	213	191	191	195	198	165	170
Aa1350	2	431	431	133	137	201	217	184	194	412	412	253	256	236	240	230	230	213	213	197	207	195	198	170	170
Aa1351	2	431	431	135	135	205	223			412	412	250	253	258	274	230	230	209	213	189	189	195	198	165	170
Aa1352	2	427	429	139	139	199	207	182	182	412	418	250	253	236	236	230	230	205	205	197	197	195	198	175	190
Aa1353	2	429	431	125	157	213	231	186	186	412	418	250	253	230	230	230	235	213	213	191	191	192	195	165	170
Aa1354	2	431	431	169	169	213	217	194	194	412	412			238	238	230	230			191	191	198	198	170	170
Aa1355	2	431	431	171	171	201	231	184	194			250	256	236	236	230	230	205	213	207	207	198	201	190	190
Aa1356	2	429	429	137	137	217	217	182	206			256	256	236	236	230	230	205	213	197	197	195	195	175	175
Aa1357	2	427	429	137	137	203	221	182	206	412	412	253	256	248	264	230	230			191	191	195	198	170	170
Aa1358	2	427	431	143	167	205	231	182	182	412	412	250	250	226	236	230	230	213	213	191	207	195	195	170	170
Aa1359	2	429	431	137	137	221	231	182	206	412	412	241	256	236	260			209	213	193	193	198	201	170	175
Aa1360	2	431	431	125	137	217	221	198	206	412	412	250	250	236	236	230	230	209	213	191	197	195	195	170	190
Aa1361	2	427	427	133	157	217	217	184	192	412	412	250	256	228	264	230	230	193	213	207	207	195	198	170	190
Aa1362	2	427	427	133	133	217	217	184	206	412	412	250	250	238	238	235	235	209	213	207	207	198	201	170	175
Aa1363	2	429	431	135	137	201	207	182	182	412	412	250	256	280	280	230	230	213	213	207	207	195	201	170	175
Aa1364	2	429	429			207	207	182	182	412	412	250	250	238	238	230	230	213	213	191	207	195	195	170	190
Aa1365	2	429	431			197	203	182	182	412	412	250	250	236	238	235	235	205	205	191	207	198	201	170	170
Aa1366	2	431	431	139	139	201	201	182	194	412	412	256	256	322	322	230	235	205	205	191	207	195	195	175	190
Aa1367	2	427	431	125	137	189	213	186	194	412	412	256	256	236	236	225	235	209	213	207	207	198	201	165	190
Aa1368	2	431	431	125	137	205	213	188	208	412	412	250	256	264	322	225	225	205	213	191	191	195	198	170	190
Aa1369	2	427	427	125	137	217	227	194	194	412	412	250	256	248	268	230	235	193	213	191	191	195	195	170	170
Aa1370	2	427	427	159	159	201	221	182	184	412	412	250	250	236	236	230	230	213	213	207	207	195	198	165	175
Aa1371	2	431	431	133	139	213	213	182	194	412	412	250	256	238	238	230	235	193	193	207	207	198	204	170	170
Aa1372	2	431	431	137	139	211	231	182	182	412	412	253	256	236	236	230	230	201	209	191	195	195	198	170	190
Aa1373	2	427	427	137	137	203	221	186	186	412	412	256	271	232	232	230	230	213	213	207	207	195	198	190	190
Aa1374	2	425	425	137	137	231	231	206	206	412	412	250	250	238	238	230	230	213	213	207	207	195	198	175	190
Aa1375	2	431	431			243	243	182	182	412	412	256	256	236	236	230	230			191	195	195	198	165	165
Aa1376	2	429	431	133	137	197	213	182	182	412	418	250	250	236	236	230	235	209	213	195	195	195	195	170	190
Aa1377	2	429	431	137	137	201	213	182	182	412	418	250	250	236	240	230	235	201	205	207	207	195	198	175	175
Aa1378	2	427	429	141	141	201	201	182	194	412	412	250	250	236	260	230	230	193	193	191	207	195	198	170	170
Aa1379	2	429	429	137	137	195	195	182	182	412	412	253	256	240	264	230	230	205	213	191	195	198	198	170	170
Aa1380	2	431	431	139	139	195	213	182	194	412	412	244	253	242	260	230	230	213	213	191	207	195	195	170	170
Aa1381	2	427	431			195	197	192	194	412	412	253	253	236	236	230	230			191	191	195	195	170	170
Aa1382	2	429	431	137	149	219	221	182	194	412	412	250	256	240	240			209	213	207	207	195	195	170	170
Aa1383	2	429	431	135	159	213	219	184	186	412	412	253	256	238	258	230	230	201	201	207	207	195	201	165	165
Aa1384	2	431	431	137	137	213	213	186	186	412	412	256	256	264	322	230	230	201	213	207	207	195	201	170	170
Aa1385	2	431	431	137	141	213	221	182	202	412	412	250	256	228	236	230	230	209	213	207	207	195	201	165	165
Aa1386	2	431	431	139	139	217	227	184	184	412	412	250	250	236	248	230	230			207	207	195	198	170	170

Aa1387	2	429	431	139	141	207	219			412	412	250	250	236	266	230	230	213	213	207	207	195	198	190	190
Aa1388	2	431	431	125	139	201	231	182	182	412	412	241	250	236	260	230	235	205	209	191	207	195	195	170	170
Aa1389	2	429	431			201	231	182	182	412	412	250	250	238	238	235	235	209	213	191	191	195	198	165	175
Aa1390	2	429	431	125	137	211	219	182	182	412	412	250	256	264	264	235	235	205	205	191	195	195	198	170	170
Aa1391	2	427	429	125	125	201	217	182	206	412	412	256	256	238	238	235	235	205	213	197	207	195	198	170	170
Aa1392	2	427	427	125	137	201	201	194	194	412	412	253	253	236	266	230	235	201	205	207	207	195	201	175	190
Aa1393	2	431	431			201	221	182	194	412	412	250	256	322	322			205	205	191	195	195	195	170	170
Aa1394	2	431	431	125	137	201	213	194	194	412	412	253	256	236	238	230	230	205	213	191	207	195	195	175	190
Aa1395	2	429	431	137	137	201	201	182	184	412	412	256	256	236	238	230	230	205	209	207	207	195	198	165	175
Aa1396	2	431	433	125	125	187	229	182	182	412	412	250	250	236	260	230	230	201	213	207	207	198	201	170	170
Aa1397	2	427	433	125	139	217	217	194	194	412	412	256	256	236	236	230	230	209	213	207	207	198	201	165	165
Aa1398	2	431	431	135	135	195	213	182	198	412	412	256	256	230	260	230	230	201	213	191	205	195	195	170	190
Aa1399	2	431	431	137	139	199	219	184	192	412	412	253	256	236	260	230	235	209	209	191	207	195	198	170	170
Aa1400	2	429	431	125	125	203	231	182	182	412	412	253	256	236	322	230	235	205	205	197	197	195	201	170	170
Aa1401	2	431	431	135	155	213	213	182	182	412	412	253	256	228	236	230	230	213	213	191	209	198	201	190	190
Aa1402	2	431	431	137	137	205	223	182	182	412	418	256	256	238	264	230	230	205	209	191	191	195	198	170	170
Aa1403	2	431	431	137	137	205	217	182	182	412	418	256	256	236	236	230	230	209	213	195	207	195	198	175	180
Aa1404	2	431	431	125	141	199	213	182	182	412	412	250	250	236	266	230	230	205	209	191	207	195	195	170	175
Aa1405	2	431	431	133	137	213	217	184	194	412	412	253	253	236	260	230	230	209	213	191	195	195	198	170	170
Aa1406	2	431	431	137	139	199	213	184	194	412	412	256	256	238	322	230	230	205	213	207	207	195	198	175	175
Aa1407	2	427	429	137	169	213	213	182	194	412	412	250	250	240	264			201	213	207	207	198	201	165	170
Aa1408	2	427	431	125	125	195	213	182	194	412	412	244	244	236	236	230	230	201	213	195	207	195	198	170	170
Aa1409	2	429	431	137	139	197	201	194	194	412	412	250	256	260	280	230	230	205	213	191	207	195	195	165	170
Aa1410	2	429	431	139	139	201	213	194	206	412	412	250	250	242	258	230	230			191	207	195	201	175	190
Aa1411	2	427	431	137	139	205	207	182	182	412	412	250	253	236	236	230	230	205	213	207	207	195	195	170	175
Aa1412	2	427	431	137	137	213	213	182	182	412	412	253	253	238	238	230	230	213	213	205	207	195	201	170	170
Aa1413	2	427	427	157	159	211	215	188	194	412	412	250	250	260	264	230	230	201	213	207	207	195	195	190	190
Aa1414	2	427	431	137	137	203	213	182	194	412	412	256	256	236	248	230	235	209	213	191	191	195	201	175	175
Aa1415	2	431	431	137	167	217	217	192	192	412	412	256	256	236	260	230	230	205	213	191	197	195	198	170	170
Aa1416	2	429	429	137	155	211	215			412	412	256	256	236	236			209	209	191	191	195	198	170	175
Aa1417	2	427	429	143	143	201	217	192	194	412	412	253	256	236	236	230	230	205	205	207	207	195	195	170	170
Aa1418	2	431	431	125	139	201	217	182	194	412	412	250	256	236	264	230	230	209	213	193	209	198	198	165	170
Aa1419	2	429	431			213	231	184	184	412	412	250	253	236	238	230	230	201	201	195	195	198	201	170	170
Aa1420	2	427	431	139	139	201	221	182	194	412	412	241	250	236	236	230	230	205	213	207	207	195	195	175	175
Aa1421	2	431	431	135	135	201	215	182	206	412	412	244	256	236	268	230	230	213	213	191	209	195	195	170	190
Aa1422	2	431	431	125	137	213	231	192	194	412	412	250	256	228	236	230	230	205	213	191	195	198	198	190	190
Aa1423	2	431	431	137	137	201	205	186	192	412	412	253	256	236	236	230	230	205	205	191	207	195	198	175	190
Aa1424	2	427	431	135	137	231	231	182	184	412	412	244	256	248	248	230	230	213	213	191	191	195	195	175	190
Aa1425	2	427	431	137	137	199	223	182	194	412	418	253	256	236	240	230	230	213	213	191	207	195	198	170	190
Aa1426	2	431	431	133	133	199	199	182	206	412	412	253	256	236	240	230	230	205	213	191	207	198	201	175	190
Aa1427	2	429	429	125	137	201	217	182	206	412	418	250	250	260	264	235	235	213	213	191	209	195	201	170	170
Aa1428	2	427	427	137	137	211	231	182	182	412	418	256	256	236	236	230	230	213	213	191	191	195	198	165	170
Aa1439	3	427	431	137	137	201	231	182	206	412	412	253	253	230	258	230	230	205	213	197	197	198	198	170	175
Aa1440	3	431	431	137	137	201	231	182	184	412	412	253	253	236	236	230	230	205	213	207	207	195	198	170	170
Aa1441	3	427	427	135	135	215	215	182	194	412	412	253	253	236	236	235	235	201	201	191	195	192	195	175	175
Aa1442	3	431	431	137	137	203	213	182	182	412	412	250	250	230	322	230	230	205	213	191	207	195	198	170	170
Aa1443	3	427	429	125	125	213	213	182	194	412	412	250	250	258	258	230	230	201	205	207	207	195	195	170	170
Aa1444	3	427	429	137	137	205	229	186	186	412	412	241	253	264	322	230	230	201	205	195	197	195	195	170	170
Aa1445	3	427	427	137	137	213	231	184	184	412	418	250	256	236	236	230	230	201	201	191	195	195	195	170	180
Aa1446	3	431	431	139	143	199	201	194	198	412	412	250	250	316	316	230	230	209	213	207	207	195	195	170	170
Aa1447	3	427	427	137	155	213	213	192	194	412	412	253	256	236	260			213	213	191	191	195	198	165	175
Aa1448	3	429	431	137	143	213	217	182	206	412	412			236	236	230	230	213	213	191	207	195	201	190	190
Aa1449	3	431	431	137	143	213	231	182	182	412	412	241	241	236	260	230	230	205	213	191	207	195	198	190	190
Aa1450	3	431	431	137	137	213	231	192	194	412	412	253	253	240	240	230	230	213	213	207	207	195	195	170	170
Aa1451	3	427	431	157	157	213	217	186	206	412	418	250	253	236	236	230	230	205	209	189	191	195	195	175	175
Aa1452	3	429	429	139	139	205	213	194	194	412	418	250	256	230	236	230	230	201	205	191	197	198	198	170	170
Aa1453	3	431	431	125	137	205	221	182	182	412	412			258	264	230	235	213	213	191	195	195	198	170	190
Aa1454	3	431	431	139	141	203	213	182	182	412	412	250	256	230	230	230	230	193	213	195	197	195	201	170	175
Aa1455	3	429	431	137	139	195	201	192	194			256	256	236	264	230	235	205	213	191	191	195	195	170	175
Aa1456	3	427	427	137	137	201	213	192	194	412	418	250	250	268	268	235	235	209	213	207	207	195	198	190	190
Aa1457	3	431	433	125	159	187	195	188	192	412	418	253	256	268	268	230	230	201	209	207	207	195	198	175	190
Aa1458	3	427	431	137	137	201	221	182	182	412	418	253	253	236	264	230	235	209	213	207	207	195	198	170	175
Aa1459	3	431	431	139	139	199	231	182	194	412	412	250	256	258	258	230	230	213	213	197	207	195	198	175	175
Aa1460	3	431	431	139	153	217	231	182	206	412	412	250	250	230	236			213	213	191	195	195	198	190	190
Aa1461	3	429	431	169	169	201	213	182	192	412	412	250	250	236	236	230	230	209	213	207	207	198	198	170	175
Aa1462	3	431	431	137	137	213	213	182	182	412	412			236	248	230	230	209	213	207	207	195	198	170	175
Aa1463	3	427	427	137	137	201	201	186	194	412	412	250	253	236	258	230	235	193	213	191	195	195	201	170	170
Aa1464	3	427	427	125	125	199	249	182	182	412	418	250	253	248	248	235	235	205	213	191	191	195	201	170	170
Aa1465	3	427	431	137	137	201	205	198	198	412	412	253	253	248	264	230	230	209	213	191	195	195	201	170	170
Aa1466	3	427	429	125	155	205	217	192	192			250	250	238	238	225	230	213	213	191	195	198	198	170	175
Aa1467	3	427	429	125	125	217	231	194	194	412	412	250	256	238	238	235	235	201	209	191	207	198	198	175	175
Aa1468	3	431	431	135	135	213	231	182	194	412	418	250	250	258	260			209	213	191	191	198	201	175	175
Aa1469	3	427	429	137	139	197	205	182	202	412	418	250	271	238	314	230	230	213	213	191	191	195	195	165	170
Aa1470	3	429	431	135	137	201	217	182	182	412	418	253	253	238	248	230	230	201	209	207	207	195	198	175	175
Aa1471	3	427	431	137	137	187	231			412	412	250	256	236	264	230	235	209	213	191	207	195	201	170	175
Aa1472	3	429	431	137	137	207	217	182	206	412	412	250	253	236	236			205	205	195	195	195	195	170	170
Aa1473	3	429	429	137	137	205	221	182	194	412	418	250	250	258	258	230	230	205	217	191	195	195	198	170	175
Aa1474	3	427	427	137	139	199	219	182	182	412	412	250	256	236	236	230	230	209	213	191	191	195	201	175	175

Aa1475	3	427	431	139	139	187	201	182	182	412	412	250	256	322	322	230	230	205	205	205	207	195	201	170	170
Aa1476	3	431	431	137	137	201	231	182	182	412	412	250	253	258	264	230	230	209	213	207	207	195	198	170	190
Aa1478	3	429	431	125	143	191	217	186	186	412	418	253	256	236	242	230	230	213	213	205	207	195	195	170	170
Aa1479	3	427	427	125	139	201	215	182	182	412	412	253	253	236	264	230	230	209	213	207	207	195	198	175	175
Aa1480	3	427	431			211	211	194	206	412	412	250	256	248	248	230	235	193	213	191	209	195	198	175	190
Aa1481	3	431	431	137	137	205	213	182	184	412	412	256	256	238	282	230	230	201	213	207	207	195	195	170	170
Aa1482	3	429	431	137	137	205	219	182	184	412	412	250	256	260	260	230	230	205	213	207	207	195	195	170	175
Aa1483	3	431	431	143	143	197	231	182	182	412	412	250	250	264	264	230	230	213	217	191	191	195	198	190	190
Aa1484	3	427	431	125	139	205	215	194	194	412	412	241	241	230	236	230	230	213	213	191	197	198	201	170	190
Aa1485	3	427	431	137	137	201	217	192	194	412	418	253	256	236	238	230	230	213	217	195	195	198	201	190	190
Aa1486	3	429	431	137	137	213	213	208	208	412	412	250	256	230	248	230	230	201	213	197	197	195	198	165	170
Aa1487	3	431	431	137	137	201	229			412	412	250	250	236	236	230	230	209	213	207	207	195	195	190	190
Aa1488	3	427	431	137	143	201	221	184	194	412	418	250	250	264	264	230	230	213	213	195	195	195	198	170	170
Aa1489	3	427	427	137	137	217	221	182	184	412	412	253	253	276	322	230	230	213	213	191	195	195	198	170	170
Aa1490	3	431	431	125	125	195	213	182	192	412	412	250	250	236	322	230	230	213	213	191	195	195	195	170	170
Aa1889.1	3	431	431	137	137	187	201	182	182	412	412	244	256	236	238	230	230	213	213	207	207	195	198	170	170
Aa1890.1	3	431	431	125	125	201	231	192	192	412	418	250	250	238	238	230	230	213	213	207	207	195	195	175	190
Aa1891.1	3	431	431	137	137	217	221	182	194	412	412	250	250	248	248	230	230	213	213	207	207	195	198	175	190
Aa1892.1	3	429	429	157	157	213	217	194	194	412	412	250	250	236	238	230	230	205	213	207	207	195	198	170	170
Aa1893.1	3	431	431	141	159	211	213	182	192	412	412	250	250	236	236	230	230	209	213	195	205	195	198	190	190
Aa1894.1	3	431	431	137	137	213	219	182	206	412	412	247	256	230	238	230	230	201	213	191	191	198	198	170	190
Aa1895.1	3	429	431	125	143	199	205	192	192	412	412	256	271	236	236	230	230	201	213	207	207	198	198	170	185
Aa1896.1	3	431	431	137	137	201	231	182	192	412	412	256	256	236	268	230	230	205	209	191	197	198	198	170	170
Aa1897.1	3	429	431	137	139	203	213	194	206	412	418	241	241	236	238	230	230	205	213	191	207	195	195	170	170
Aa1898.1	3	427	431	139	139	213	217	192	192	412	418	250	253	236	280	230	235	209	209	191	207	195	201	170	175
Aa1899.1	3	427	431	137	137	201	231	182	192	412	412	250	253	240	260	235	235	205	213	191	207	198	201	170	170
Aa1900.1	3	427	429	137	165	213	221	192	192	412	412	250	256	236	264	230	230	213	217	191	197	198	198	170	170
Aa1902.1	3	429	431	137	137	187	219	182	194	412	418	253	253	320	320	230	230	213	213	191	207	195	198	170	190
Aa1903.1	3	431	431	137	141	187	195	194	194	412	412	250	271	236	236	230	230	209	213	205	207	195	198	165	175
Aa1904.1	3	425	429	125	125	213	217	186	192	412	412	256	256	224	270	230	230	205	213	195	207	195	201	175	175
Aa1905.1	3	427	429	133	139	201	205	186	186	412	418	256	256	258	320			213	213	191	191	195	201	170	170
Aa1906.1	3	429	431	137	139	213	213	192	194	412	418	256	256	236	238	230	230	201	213	191	195	195	198	165	165
Aa1907.1	3	429	431	137	137	219	219	194	206	412	418	250	250	240	260	230	235	193	213	191	195	198	198	170	170
Aa1908.1	3	427	431	137	137	195	231	182	194	412	418	253	253	238	258	230	230	213	213	207	207	195	198	170	190
Aa1909.1	3	427	431	123	139	201	201	182	182	412	412	250	256	230	236	230	230	205	205	195	207	195	195	170	175
Aa1910.1	3	431	431	139	139	213	231	182	206	412	412	250	256	238	320	230	230	213	213	207	207	195	201	165	175
Aa1911.1	3	427	433	157	157	213	221	182	182	412	412	253	253	280	280			201	213	207	207	195	201	170	170
Aa1912.1	3	427	429	137	139	205	217	184	192	412	412	250	256	232	314	230	230	201	213	191	191	195	201	170	170
Aa1913.1	3	431	431	141	141	195	201	198	198	412	418	241	241	236	236	230	235	205	213	207	207	195	198	170	170
Aa1914.1	3	431	431	137	165	207	217	182	206	412	412	241	256	236	236	230	230	205	213	197	207	195	198	170	170
Aa1915.1	3	431	431	125	125	213	217	182	182	412	418	253	253	236	322	230	230	201	205	207	207	198	198	175	190
Aa1916.1	3	431	431	137	167	205	217	192	194	412	412	250	256	264	264	230	230	209	213	191	197	195	198	170	170
Aa1917.1	3	427	431	143	143	201	201	194	194	412	412	256	256	236	236	230	230	213	213	191	191	195	201	175	175
Aa1918.1	3	431	431	125	125	201	201	184	184	412	412	250	250	230	322	230	230	209	213	207	207	195	201	190	190
Aa1919.1	3	429	431	137	141	217	217	182	192	412	412			236	238	230	235	213	213	197	197	198	198	170	175
Aa1920.1	3	425	427	155	155	201	221	192	192	412	412	250	253	236	236	230	230	201	213	195	195	195	195	165	170
Aa1921.1	3	429	429	137	139	187	201	194	194	412	412	250	256	238	264	230	230	213	213	195	195	195	198	170	170
Aa1922.1	3	431	431	137	139	201	213	182	198	412	418	241	241	264	268	230	235	209	213	191	207	195	201	170	170
Aa1923.1	3	425	427	125	137	201	201	182	182	412	418	253	271	236	238	230	230	213	213	191	197	195	198	170	170
Aa1924.1	3	427	429	137	137	199	203	182	192	412	418	253	256	248	248	230	235	193	201						
Aa1925.1	3	431	431	137	137	219	223	182	194			250	256	224	314			213	213	207	207	195	201	170	170
Aa1926.1	3	429	429	137	165	205	219	194	194	412	412	256	256	236	236	225	230	209	213	191	191	195	195	170	170
Aa1927.1	3	427	427	139	139	211	217	194	194	412	412	253	253	236	238	230	230	213	213	207	207	195	195	170	190
Aa1928.1	3	431	431	137	137	195	215	184	194	412	412	250	256	232	264	230	230	205	209	207	207	198	201	175	175
Aa1929.1	3	431	431	137	137	203	207	182	194	412	412	253	253	228	230	230	235	213	213	205	205	195	198	170	170
Aa1930.1	3	431	431	125	125	201	221	182	182	412	412	253	256	236	240	230	230	213	213	193	195	195	201	175	175
Aa1931.1	3	431	431	137	143	211	219	182	182			256	256	236	238	230	235	213	213	191	191	198	198	175	175
Aa1932.1	3	427	431	167	167	187	217	194	194	412	412	253	253	236	240	230	230	213	213	191	207	198	198	170	170
Aa1933.1	3	431	431	165	165	215	221	182	182	412	412			248	264	230	230	213	217	191	191	195	201	170	170
Aa1934.1	3	429	431	137	137	217	231	182	182	412	412	256	256	236	322	230	235	213	213	207	207	195	198	170	170
Aa1935.1	3	431	431	139	169	213	223	182	194	412	418	256	256	236	238	230	230	213	213	207	207	195	201	170	175
Aa1936.1	3	427	429	125	137	205	221	182	184	412	412	250	253	236	236	230	230	213	213	191	207	198	201	170	170
Aa1937.1	3	429	439	141	155	201	217	194	194	412	412	250	253	236	240	230	230	213	213	191	205	201	201	170	170
Aa1939.1	3	429	431	137	141	201	217	182	194	412	412	253	256	236	236	230	230	205	209	195	207	195	195	170	170
Aa1940.1	3	427	431	133	139	217	221	194	194	412	412	250	253	236	236	230	230	205	213	195	195	198	198	170	170
Aa1549	4	427	431	137	137			182	184	412	412	256	256	238	238	230	230			195	207	195	201	170	170
Aa1550	4	427	429	125	125	215	231	182	184	412	412	250	256	236	236	230	230	213	213	197	207	195	201	170	170
Aa1551	4	427	427	125	137	217	229	194	194	412	412	250	256	236	236	230	230	205	213	191	207	195	195	165	170
Aa1552	4	431	431	125	125	213	213	182	194	412	412			266	320	235	235	201	213	207	207	195	198	170	170
Aa1553	4	431	431	137	137	201	201	194	194	412	418	247	250	260	264	230	230	213	213	195	205	195	198	170	170
Aa1554	4	431	431	153	153	201	213	182	182	412	418	250	253	258	264	230	235	205	213	207	207	195	201	170	180
Aa1555	4	429	431	137	137	201	213	182	182	412	412	253	256	236	248	230	230	201	201	197	207	195	201	170	170
Aa1556	4	431	431			187	209	194	194	412	412	250	253	236	238	230	230	213	213	195	197	195	198	175	175
Aa1557	4	431	431	123	123	221	221	192	192	412	412	256	256	242	242	230	230			207	207	195	198	165	165
Aa1558	4	429	431	125	139	201	221	182	182	412	412	253	253	236	242	230	230	201	213	195	207	198	201	170	170
Aa1559	4	429	431	137	159	211	233	192	192	412	412	253	253	238	238	230	230	209	213	207	207	195	198	170	175
Aa1560	4	429	431	137	137	201	213	182	192	412	412	256	256	236	264	230	230	213	213	191	207	195	198	170	170
Aa1562	4	431	431	137	137	201	213	194	194	412	412	241	250	236	236	235	235	205	213	207	207	195	198	170	175

Aa1563	4	427	431	125	137	229	229	186	192	412	412	256	256	240	240	230	230	201	209	191	207	195	201	170	190
Aa1564	4	429	433	137	137	201	217	192	198	412	418	250	256	236	264	225	225	201	213	189	207	195	195	170	170
Aa1565	4	427	431	125	139	205	217	170	170	412	412	250	250	236	236	230	230	213	213	191	207	198	198	170	170
Aa1566	4	431	431	137	137	201	205	184	184	412	418	253	253	236	238	230	230	201	209	191	191	198	201	175	175
Aa1567	4	427	431	137	139	217	217	182	182	412	412	250	256	236	236	230	230	205	213	189	207	195	198	175	175
Aa1568	4	429	431	137	137	217	231	184	202	412	412	256	256	236	238	230	230	201	213	191	207	195	198	170	190
Aa1569	4	431	431	125	137	221	221			412	412	253	253	236	236	230	230	205	213	195	207	195	198	170	175
Aa1570	4	431	431	137	137	187	201	192	192			250	250	236	270	230	230	213	213	189	189	195	201	165	170
Aa1571	4	429	431	125	137	197	217	182	182	412	412	241	241	236	236	230	230	209	209	191	207	198	198	185	185
Aa1572	4	429	431	137	137	205	217	184	186	412	412	253	253	236	238	230	230	209	213	197	197	195	195	170	170
Aa1573	4	429	429	137	159	203	217	182	192	412	412	256	256	236	240	230	230	209	213	191	195	198	198	170	170
Aa1574	4	425	431	141	155	217	217	182	194	412	412	250	250	236	248	230	230	205	213	191	191	198	201	165	165
Aa1575	4	427	431	133	157	199	217	182	202	412	412	250	256	236	240	230	230	213	213	207	207	195	198	170	180
Aa1576	4	427	427	133	137	205	231	194	202	412	412	250	250	236	320	230	230	213	213	191	191	195	198	190	190
Aa1577	4	427	429	137	137	199	207	184	184	412	412	250	256	238	264	230	235	201	213	191	195	195	201	175	175
Aa1578	4	429	429	137	137	201	221	192	194	412	412	250	253	264	264	230	230	209	213	195	197	195	198	165	190
Aa1579	4	431	431	137	137	213	213	182	182	412	412	253	253	236	270	230	230	193	209	191	191	195	195	170	175
Aa1580	4	429	431	125	125	207	211	184	194	412	412	244	253	236	264	230	230	213	213	191	197	195	201	165	170
Aa1581	4	431	431	137	139	213	221	192	192	412	412	256	256	236	236	230	230	213	213	207	207	195	195	170	175
Aa1582	4	431	431	125	137	201	231	182	186	412	412	250	253	236	264	230	230	213	213	191	195	198	198	170	190
Aa1583	4	427	429	137	137	191	217	182	198	412	412	250	253	240	260	230	230	213	213	191	197	195	198	175	190
Aa1584	4	427	427	165	165	217	221	194	202	412	412	253	256	236	236	230	230	201	213	207	207	195	198	170	185
Aa1585	4	427	429	137	137	213	231	182	182	412	412	250	250	236	240	230	235	213	213	191	191	195	201	170	170
Aa1586	4	431	431	133	133	201	205	182	194	412	412	253	256	228	236	230	230	201	213	191	191	195	201	165	170
Aa1587	4	427	431	153	153	201	231	192	192	412	412	250	250	264	268	230	230	201	213	191	197	195	198	170	175
Aa1588	4	429	431	137	141	213	223	182	182	412	412	256	274	236	236	230	230	201	205	191	207	195	195	190	190
Aa1590	4	429	431	135	135	187	201	192	192	412	418	250	253	240	258	230	230	209	209	191	191	201	201	170	170
Aa1591	4	429	429	125	137	201	217	184	198	412	412	253	256	260	264	230	235	213	213	207	207	195	195	175	175
Aa1592	4	425	431	125	155	201	203	194	194			253	253	240	240	230	230	209	209	207	207	195	198	175	175
Aa1593	4	427	427	125	165	195	211	192	192	412	412	253	253	226	240	230	230	209	213	191	191	195	201	170	170
Aa1594	4	427	427	137	137	231	231	206	206	412	412	250	250	236	236	230	235	205	213	207	207	195	195	175	190
Aa1595	4	427	431	155	155	201	205	192	206	412	412	241	256	236	258	230	230	213	213	191	207	195	198	190	190
Aa1596	4	431	431	137	137	183	231	192	192	412	418	253	253	240	240	230	230	201	213	191	209	195	198	170	170
Aa1597	4	429	429	125	125	201	209	182	182	412	412	253	253	240	240	230	235	209	209	191	195	195	198	175	175
Aa1598	4	429	431	133	133	203	221			412	412	253	256	236	264	230	230	209	213	207	207	195	195	170	170
Aa1599	4	431	431	125	139	221	221	182	184	412	412	253	256	236	236	230	230	213	213	207	207	195	195	190	190
Aa1600	4	431	431	155	167	215	221	194	194	412	412	250	256	236	248	230	230	201	213	191	207	195	198	170	190
Aa1601	4	427	429	137	153	207	207	194	198	412	412	250	259	240	260	230	230	205	209	191	207	195	198	190	190
Aa1602	4	427	427	125	125	205	207	194	194	412	412	250	253	240	240	230	235	205	213	191	207	198	198	175	175
Aa1603	4	427	427	137	137	217	217	184	184	412	412	250	256	236	236	230	235	201	201	191	207	195	198	170	175
Aa1604	4	429	431	141	153	213	231	182	186	412	412	256	256	236	236	230	230	201	213	191	191	195	198	165	165
Aa1605	4	431	431	141	141	213	231	182	194	412	412	256	256	236	260	230	230	209	213	191	195	198	198	190	190
Aa1606	4	431	431	135	135	205	221	192	194	412	412	250	256	236	270	230	230	201	201	195	195	198	201	175	190
Aa1607	4	431	431	137	139	211	219	182	192	412	412	250	250	236	322	230	230	213	213	193	195	195	195	170	170
Aa1608	4	431	431	137	137	199	199	184	206	412	412	250	256	236	236	230	230	209	209	191	197	195	201	170	175
Aa1609	4	431	431	137	141	203	207	182	184	412	412	256	256	240	260	230	230	205	213	191	207	198	198	170	175
Aa1610	4	431	431	125	125	217	217	192	192	412	412	250	256	236	268			193	213	191	207	195	195	170	170
Aa1611	4	427	431	137	137	205	213			412	412	250	250	258	270	230	230	205	213	191	191	198	201	175	175
Aa1612	4	427	427	137	155	195	219	194	194	412	412	250	250	240	270	230	230	201	213	191	207	195	198	170	170
Aa1613	4	427	427	137	137	217	219	182	182	412	412	250	256	238	264	235	235	213	213	207	209	195	198	170	170
Aa1614	4	429	429	133	137	217	217	192	206	412	412	250	250	236	238	225	225	213	213	205	205	195	198	170	175
Aa1615	4	431	431	133	137	205	205	182	182	412	412	250	256	236	320	230	230	205	213	197	197	198	198	165	170
Aa1616	4	431	431	133	135	205	217	170	182	412	412	256	256	236	236	230	230	213	213	207	207	198	201	170	170
Aa1617	4	427	427	125	137	213	213	192	192	412	412	256	256	240	258	230	230	209	213	191	195	195	195	170	170
Aa1618	4	427	431	137	137	201	217	182	182	412	412	256	256	270	314			193	205	191	191	195	195	170	180
Aa1619	4	427	427	137	137	213	221	182	194	412	412	253	256	228	238	230	230	209	213	197	207	195	198	170	170
Aa1620	4	429	429	125	137	187	195	194	194	412	412	250	250	236	264	230	235	213	213	189	195	195	195	175	175
Aa1621	4	429	429	125	165	207	217	182	194	412	412	253	256	236	264	230	230	209	213	191	191	195	198	170	170
Aa1622	4	429	431	125	125	213	217	182	194	412	412	244	256	236	236	230	230	213	213	207	207	195	198	170	190
Aa1623	4	429	429	125	143	207	213	206	206	412	412	250	256	230	320	230	230	201	213	195	197	195	198	165	175
Aa1624	4	431	431			219	219			412	412					230	230	205	213	195	207	198	201	170	175
Aa1625	4	427	427	125	137	201	201	194	202	412	412	253	256	236	260	230	230	201	213	191	191	195	198	165	190
Aa1626	4	429	431	125	139	195	235	182	192	412	412	250	256	236	236	230	230	205	213	191	191	198	201	170	175
Aa1627	4	427	431	137	137	195	219	182	186	412	412	250	256	236	238	230	235	209	213	191	191	195	198	170	175
Aa1628	4	429	431	125	137	195	231	184	194	412	418	250	256	238	264	230	230	209	209	207	207	195	198	170	170
Aa1629	4	431	431	135	135	199	205	182	194	412	412	253	253	230	230	225	225	217	217	191	197	195	195	170	175
Aa1630	4	427	431	125	165	201	231	182	182	412	418	250	256	264	264	230	230	209	209	207	207	195	198	165	170
Aa1631	4	427	427	141	141	199	217	184	192	412	412	256	256	322	322	230	230	201	213	207	207	195	198	175	175
Aa1632	4	431	431	167	167	201	213	192	192	412	418	250	256	236	236	230	230	205	209	207	207	195	198	175	175
Aa1633	4	431	431	125	125	205	219	182	198	412	412	250	253	236	264			205	205	195	207	195	201	165	170
Aa1634	4	427	429	137	165	205	205	182	184	412	412	253	256	236	320	230	230	193	213	207	207	195	201	175	175
Aa1635	4	431	431	137	137					412	418			248	248	230	230			207	207	195	195	170	170
Aa1636	4	431	431	137	137	219	231	188	188	412	412	250	256	236	236	230	230	205	205	191	191	195	198	170	170
Aa1637	4	427	429	137	137	187	225	182	198	412	412	256	256	240	240	230	230	213	213	195	207	195	195	175	180
Aa1638	4	431	431	169	169	195	217	184	192	412	412	250	250	242	264	230	235	213	213	191	191	195	198	175	175
Aa1639	4	431	431	169	169	211	217	182	196	412	412	256	256	238	240	230	230	209	213	191	195	195	198	165	165
Aa1640	4	431	431	125	125	203	213	182	182	412	412	253	253	236	258	230	230	205	209	207	207	195	195	170	170
Aa1641	4	429	431	137	149	221	221	186	194	412	412			236	240	230	230	213	213	191	191	198	201	170	170

Aa1642	4	431	431	125	125	201	207	194	196	412	412	250	253	260	264	230	235	213	213	191	205	195	195	170	170
Aa1643	4	429	431	137	137	213	213	194	194	412	412	253	253	236	264	230	230	205	205	195	195	195	198	170	175
Aa1644	4	431	431	137	139	199	213	182	184	412	412	256	256	230	238	230	235	205	205	205	207	195	201	190	190
Aa1645	4	429	431	157	165	213	213	192	194	412	412	253	253	248	286	230	230	201	213	191	191	201	201	170	185
Aa1646	4	427	427	137	137	213	217	182	182	412	412	253	256	236	322	230	230			191	191	195	198	170	170
Aa1647	4	431	431	155	157	205	231	182	194	412	412	250	256	230	236			205	205	191	191	198	198	170	170
Aa1648	4	429	431	139	141	205	205	194	202	412	412	250	253	236	238	230	230	213	213	191	197	195	201	170	180
Aa1659	5	431	431	125	137	213	213	184	192	412	418	250	256	236	236	230	230	201	205	191	191	195	195	185	185
Aa1660	5	427	427	137	137	201	213	182	182	412	418	256	256	238	248	230	230	205	209	191	207	198	201	170	170
Aa1661	5	431	431	133	155	201	207	182	182	412	412	244	256	236	248	230	230	205	213	191	207	195	195	170	170
Aa1662	5	427	429	137	137	201	217	192	202	412	412	253	256	230	248	230	230	209	213	207	207	195	201	170	170
Aa1663	5	427	431	139	143	187	217	184	206	412	412	253	256	236	236	230	230	213	213	195	207	195	201	170	175
Aa1664	5	429	429	133	133	213	227	206	206	412	412	250	256	236	236	235	235	209	213	191	191	198	198	170	175
Aa1665	5	431	431	125	137	201	219	194	206	412	412	250	250	240	264	225	225	209	213	207	209	195	198	165	170
Aa1666	5	431	431	137	141	187	221	182	194	412	412	250	256	236	260	230	230	205	213	195	209	195	198	175	175
Aa1668	5	427	431	137	165	207	217	182	194	412	412	253	256	230	230	230	235	213	213	197	207	195	198	170	170
Aa1669	5	429	431	137	137	201	211	182	182	412	412	256	256	236	238	230	230	213	213			195	201	170	175
Aa1670	5			137	137			182	182	412	412			226	240	230	230	201	205	189	195	195	198	170	190
Aa1671	5	431	431	139	155	201	201	182	182	412	412	256	256	248	280	235	235	205	209	191	205	195	195	170	180
Aa1672	5	431	431	137	137	211	217	182	182	412	412	256	256	238	248	230	230	213	213	205	207	195	198	170	170
Aa1673	5	431	431	125	137	199	199	182	194	412	412	253	256	230	236	230	230	205	205	191	191	195	198	170	175
Aa1674	5	427	431	125	137	221	221	182	182					236	240	230	230	205	205	197	207	198	198	170	170
Aa1675	5	431	431	155	157			184	184	412	412	241	250	228	236	230	230			191	191	195	198	165	165
Aa1676	5	429	431	135	137	203	205	184	194	412	412	256	256	238	238	230	230	213	213	191	191	195	198	165	165
Aa1677	5	431	431	125	137	201	211	182	182	412	412	250	253	230	230	230	230	209	213	207	207	195	198	175	190
Aa1678	5	427	427	137	139			182	182	412	412			236	236	230	230			191	207	195	195		
Aa1679	5	427	427	137	137	205	231	182	182	412	412	250	250	236	264			205	213	197	207	192	195	170	170
Aa1680	5	427	431	125	125	209	209	198	198	412	418	250	256	238	238	230	230	209	213	191	197	198	198	175	175
Aa1681	5	427	427	137	139	211	217	182	192	412	412	250	250	270	270	225	225	213	213	191	191	195	198	175	175
Aa1682	5			137	139	205	217	184	184	412	412					230	230	205	213	189	209	195	198		
Aa1683	5	431	431	137	143	217	231	182	182	412	412	250	256	228	236			205	209	207	207	198	198	170	170
Aa1684	5	431	431	137	137	187	201	182	184	418	418	244	244	230	236	230	230	213	213	207	207	195	198	170	170
Aa1685	5	427	429	137	155	201	225	182	194	412	412	256	256	236	236	230	230	201	213	191	191	198	198	170	180
Aa1686	5	427	427	137	137	213	223	182	184	412	412	256	256	232	236	230	230			191	207	195	195	175	175
Aa1687	5	431	431	139	139	213	217	182	182	412	412	256	256	236	236	230	230	201	201	191	191	198	201	190	190
Aa1688	5	431	431	139	143	187	205	194	194	412	412	253	253	240	240	230	230	213	213	191	191	195	198	190	190
Aa1689	5	431	431	125	125	197	201	182	182	412	412	244	244	238	264	230	230	209	213	207	207	198	204	190	190
Aa1690	5	429	431	125	137	201	217	194	194	412	412	253	256	236	266			213	213	207	207	198	198	165	170
Aa1691	5	427	431	137	137	213	217	184	184	412	412	250	253	236	236	230	230	209	213	207	207	198	201	170	170
Aa1692	5	427	427	125	155	219	227	182	194	412	412	244	244	260	264	230	230	213	213	195	195	195	201	170	175
Aa1693	5	427	427	165	167	217	231	182	182	412	418	250	253	236	316	230	230	209	209	205	207	195	195	170	175
Aa1694	5	427	429	137	137	205	205	182	182	412	412			230	230	230	230			207	207	195	201	170	170
Aa1695	5	429	431	155	157	197	217	194	194	412	412	253	256	228	228	230	230	201	213	207	207	195	195	170	170
Aa1696	5	429	429			211	217	182	182	412	412	256	256	230	230	230	230			207	207	195	195	190	190
Aa1697	5	429	431	125	137	201	213	182	182	412	412	256	256	238	248			205	205	191	207	195	195	170	170
Aa1698	5	431	431	137	167	213	213	182	184	412	412	250	250	236	270			193	205	189	189	195	201	170	170
Aa1699	5	429	429	143	143	191	195	182	194	412	418	250	253	236	240	230	235	205	213	191	191	195	195	175	175
Aa1700	5	431	431	137	137	203	217	182	184	412	412	253	256	238	240	230	235	205	205	207	207	198	204	170	175
Aa1701	5	431	431	125	155	213	217	194	206	412	412	256	256	230	238			193	213	207	207	195	195	170	170
Aa1702	5	429	431	137	137	201	231	184	184	412	412	256	256	238	238	230	230	213	213	207	207	195	198	165	175
Aa1703	5	429	429	137	137	205	213	182	182	412	418	250	256	258	258	230	230	213	213	191	191	195	198	170	170
Aa1704	5	427	427	139	139	219	221	182	186	412	412	256	256	238	264	230	230	205	209	191	207	198	198	170	170
Aa1705	5	429	429	137	143	213	231	182	194	412	412	241	250	230	248	230	230	209	209	191	207	195	195	170	190
Aa1706	5	431	431	139	155	213	231	182	182	412	412	241	241	280	280	230	230	213	213	191	191	195	198	170	190
Aa1707	5	427	431	135	137	217	217	184	198	412	412	253	256	236	236	230	230	205	205	195	195	198	201	165	190
Aa1708	5	427	431	137	139	205	221	182	192	412	412	256	256	236	264	230	230	205	209	191	207	195	198	170	190
Aa1709	5	429	431	137	153	201	205	182	182	412	412	250	256	236	280	230	230	213	213	207	207	195	195	170	170
Aa1710	5	431	431	137	137	207	213	182	182	412	412	250	253	236	240	235	235	205	213	191	195	195	201	190	190
Aa1711	5	429	429	133	137	213	215	182	182	412	412	253	253	236	246	230	230	205	209	189	207	198	201	170	170
Aa1712	5	431	431	139	139	201	205	182	198	412	412	256	256	236	264	230	230	201	213	197	207	195	195	170	170
Aa1713	5	429	431	137	165	219	219	182	184	412	412	250	250	238	238	230	230	201	201	207	207	198	198	170	170
Aa1714	5	427	427	137	165	187	213	182	194	412	412	244	244	240	240	230	230	205	213	191	191	195	198	170	170
Aa1715	5	431	431	125	137	201	213	194	194	412	412	256	256	230	230	230	230	213	213	191	191	195	201	170	175
Aa1716	5	425	431	125	125	211	217	182	182	412	412	250	256	240	240	230	230	213	213	207	209	198	198	165	170
Aa1717	5	427	427	155	157	195	195	188	192	412	412	244	244	264	264	230	230	213	213	207	207	195	195	190	190
Aa1718	5	429	431	137	139	199	227	182	182	412	412	256	256	230	236	230	230	213	213	191	191	198	198	170	170
Aa1719	5	427	431	137	165			182	182	412	412	250	253	224	264	230	230			205	207	195	198	175	175
Aa1720	5	431	431	137	139	201	231	184	184	412	412	253	253	260	260	235	235	205	213	191	191	195	198	170	170
Aa1721	5	431	431	137	139	203	205	186	186			253	256	236	236	230	230	201	213	195	207	195	198	165	170
Aa1722	5	431	431	135	135	201	211	182	192	412	412	256	256	236	264	230	230	213	217	191	195	189	198	165	190
Aa1723	5	427	427	139	139	201	231	194	194	412	412	253	253	240	276			213	213	191	207	195	198	175	190
Aa1724	5	431	431	125	167	205	231	182	196	412	412	250	250	260	260	230	230	213	217	207	207	192	195	170	175
Aa1725	5	431	431	139	139	201	213	194	194	412	412	241	250	230	236	230	230	209	213	207	207	198	201	170	170
Aa1726	5	431	431	133	139	201	205	182	182	412	412	253	253	238	248	230	230	205	213	207	207	195	201	190	190
Aa1727	5	431	431	137	137	217	217	194	194	412	412	250	250	236	264	230	230	205	205	189	207	195	195	165	165
Aa1728	5	427	427	125	125	213	221	182	192	412	412	256	256	230	236	230	235	205	213	191	191	195	198	170	175
Aa1729	5	431	431	137	137	213	215	186	186	412	412	244	256	230	264	230	230	205	205	191	207	198	201	190	190
Aa1730	5	429	431	139	139	215	221	184	184	412	412	253	256	236	322	230	230	209	213	191	191	195	198	170	170

Aa1731	5	429	429	125	137	201	205	192	194	412	412	253	256	240	240	230	235	209	213	207	207	195	198	170	190
Aa1733	5	429	431	133	137	221	229	182	194	412	412			236	236	230	235	205	213	195	195	198	198	170	170
Aa1734	5	429	429	125	137	201	203	182	182	412	412	256	256	230	322	235	235	209	209	197	207	195	198	170	170
Aa1735	5	431	431	135	135	211	231	194	194	412	412	253	253	260	264	230	230	209	213	191	191	195	201	170	175
Aa1736	5	427	429			205	221	192	198	412	412	256	256	236	264	230	230	205	213	207	209	195	198	170	175
Aa1737	5	431	431			197	201	184	184	412	418	256	256	228	228	230	230	213	213	195	209	195	195	170	170
Aa1738	5	427	429			207	217	182	198	412	412	256	271	236	320	230	230	193	217	191	195	198	198	175	190
Aa1739	5	427	429			223	223	182	182	412	418	250	250	236	236	230	230	213	213	191	207	195	195	170	170
Aa1740	5	431	433			199	203	182	192	412	412	253	256	238	238	230	230	205	209	207	207	195	198	170	170
Aa1741	5	429	429			195	203	192	206	412	412	256	256	236	260	230	230	205	213	191	191	195	201	190	190
Aa1742	5	431	437			207	211	192	206	412	412	250	250	236	280	230	230	205	213	191	191	195	195	175	175
Aa1743	5	429	431	141	141	187	201	194	194	412	412	244	253	240	240	230	230	201	205	195	195	201	201	165	190
Aa1744	5	429	431	137	167	205	213	182	182	412	412	250	250	238	238	230	230	201	205	207	207	195	198	170	170
Aa1745	5	431	431			203	217	194	194	412	418	256	256	230	264	230	230	201	201	207	207	195	198	170	170
Aa1746	5	427	429	137	145	213	213	194	194	412	412			236	236	230	230	213	213	191	191	195	195	170	170
Aa1747	5	427	431	137	137	215	217	182	194	412	412			228	236	230	230	201	213	207	207	195	195	170	170
Aa1748	5	427	431	133	133	205	215	182	182	412	412	253	256	238	238	225	225	201	205	207	207	195	198	165	170
Aa1749	5	427	429	155	157	187	207	184	184	412	412	253	256	240	264	230	235	213	213	191	191	195	201	165	175
Aa1750	5	429	431	137	139	201	203	182	182	412	412	253	256	264	264	230	235	201	213	191	207	195	195	170	170
Aa1751	5	431	431	137	137	213	217	182	182	412	412	250	256	260	260	230	230	209	209	195	195	195	195	165	165
Aa1752	5	431	431	137	137	199	225	184	184	412	412	256	259	260	260	225	225	205	205	207	207	198	198	170	170
Aa1754	5	431	431	125	125	201	201			412	412			236	236	230	230	205	213	205	207	195	198	170	170
Aa1755	5	427	431	125	137	201	205	184	184	412	412	256	256	236	264	230	230	205	213	207	207	198	201	175	175
Aa1756	5	427	427	125	137	205	213	184	194	412	412	253	253	236	248	230	230	213	213	191	191	195	195	175	175
Aa1757	5	431	431	137	141	199	203	194	194	412	412	250	256	236	236	230	230	201	201	191	191	198	201	175	175
Aa1758	5	427	431	137	137	205	213	182	182	412	412	256	256	240	264	230	230	201	209	191	207	198	198	175	175
Aa1769	6	429	431	137	165	203	213	182	192	412	412	253	256	232	322	230	230	205	213	207	207	195	198	170	170
Aa1770	6	431	431	141	141	191	207	182	182	412	412	250	250	264	264	230	230	209	213	191	191	195	198	175	175
Aa1771	6	429	431	125	137	205	231	192	192	412	412	256	256	236	264	230	230	205	213	191	191	195	198	170	170
Aa1772	6	431	431	125	137	201	201	194	198	412	412	250	250	250	250			213	213	189	209	195	198	175	175
Aa1774	6	431	431	143	143	201	211	184	198	412	412	241	250	228	228	230	230	209	213	191	207	195	195	190	190
Aa1775	6	431	431	137	137	201	223	182	208	412	412	250	256	236	236	235	235	193	213	189	207	195	198	170	175
Aa1776	6	431	431	125	125	207	207	182	182	412	412	256	256	236	236	230	230	193	213	197	207	195	201	170	170
Aa1777	6	427	429	139	139	215	223	184	184	412	418	253	253	236	322	230	230	213	213	207	207	195	195	190	190
Aa1778	6	431	431	137	137	205	231	192	192	412	412	250	256	232	238	230	230	213	213	191	207	198	198	165	165
Aa1779	6	427	431	141	141	201	201	192	194	412	412	253	256	236	236	230	230	209	209	191	207	198	201	170	170
Aa1780	6	431	431	151	151	201	213	184	184	412	412	250	262	264	264	230	230	201	213	207	207	195	201	170	170
Aa1781	6	427	431	137	137	187	205	182	206	412	412	250	253	236	240	235	235	213	213	191	207	198	201	170	170
Aa1782	6	427	427	141	165	205	207	182	194	412	412	250	256	230	238	230	230	205	213	191	191	195	195	165	165
Aa1783	6	429	431	135	137	201	213	184	184	412	412	241	253	238	242	230	235	213	213	191	195	195	198	170	170
Aa1784	6	425	431	125	141	201	213	182	182	412	412	253	256	248	248	230	230	209	213	191	207	195	195	165	165
Aa1785	6	427	431	125	139	217	217	194	194	412	412	244	253	236	236	230	235	209	213	195	195	198	201	175	175
Aa1786	6	431	431	125	125	201	227	182	194	412	412	256	256	236	248	230	230	213	213	195	195	195	201	170	190
Aa1787	6	427	429	125	137	191	201			412	412	253	256	238	274	230	230	201	213	195	197	195	195	170	175
Aa1788	6	427	429	137	155	201	221	192	192	412	412	253	253	236	248	230	230	205	213	191	195	195	198	170	175
Aa1789	6	429	429	125	133	201	215	206	206	412	412	250	253	230	230	230	235	201	201	197	197	195	195	170	170
Aa1790	6	429	429	137	157	195	201	182	182	412	412	253	253	236	264			193	205	207	207	195	195	170	170
Aa1791	6	427	427	125	137	201	221	192	194	412	412	256	256	236	236	230	235	213	213	195	207	195	198	190	190
Aa1792	6	427	431	137	137	201	221	194	194	412	412	250	256	236	238	230	230	209	213	191	207	195	195	170	190
Aa1793	6	429	431	125	125	205	201	192	196	412	412	250	256	238	264	230	230	209	209	191	191	195	195	165	170
Aa1794	6	427	427	157	159	201	217	182	182	412	412	250	250	260	264	230	230	213	217	191	209	195	195	165	175
Aa1795	6	431	431	143	143	213	217	182	182	412	412	256	256	236	248	230	230	209	213	191	191	195	195	175	175
Aa1796	6	427	427	139	139	199	199	182	182	412	412	256	256	236	264	230	230	213	217	191	191	195	195	170	170
Aa1797	6	431	431	165	165	217	217	182	182	412	412	250	271	264	264	230	230	213	213	191	191	195	195	170	170
Aa1798	6	431	431	137	137	219	245	194	206	412	418	250	256			230	230	213	213	207	207	195	195	170	170
Aa1799	6	429	429	125	137	201	201	184	194	412	412	250	253	236	236	230	230	213	213	191	191	195	201	175	175
Aa1800	6	429	429	137	137	213	213	198	198	412	412	250	256	264	280	230	230	213	213	191	191	195	201	165	170
Aa1801	6	431	431	139	139	187	231	182	184	412	412	253	256	236	236	230	230	205	213	197	207	195	201	170	170
Aa1802	6	431	431	143	145	207	213	182	186	412	418	250	256	236	264	230	230	201	201	191	191	195	201	165	165
Aa1803	6	429	429	137	137	201	221	182	194	412	412	250	256	264	270	230	230	209	209	191	207	198	201	170	170
Aa1804	6	427	429	135	169	207	231	182	182	412	412	250	256	228	236	230	230	205	213	205	207	195	201	170	175
Aa1805	6	431	431	125	165	199	221	194	194	412	412	250	253	238	240	230	230	205	213	191	191	195	198	170	170
Aa1806	6	431	431	137	137	195	227	182	182	412	412	250	256	236	240	230	230	205	213	207	207	195	201	175	175
Aa1807	6	427	431	137	137	217	227	194	194	412	418	253	256	236	236	235	235	213	213	191	207	198	198	170	170
Aa1808	6	431	431	137	141	201	215	182	182	412	412	250	256	236	236	230	235	209	217	191	191	195	195	170	170
Aa1809	6	427	431	125	125	205	217	184	192	412	412	250	250	236	238	230	230	205	213	207	207	195	201	170	170
Aa1810	6	427	429	125	137	187	217	182	184	412	412	256	256	264	280	230	235	193	213	191	191	198	198	170	170
Aa1811	6	429	431	137	157	201	213	182	192	412	418	250	256	230	236	235	235	209	209	207	207	195	195	170	175
Aa1812	6	427	429	137	137	203	213	182	182	412	412	250	259	238	260	230	230	193	213	195	195	195	201	170	175
Aa1813	6	429	429	137	137	217	221	192	192	412	412	256	259	236	236	230	230	209	213	207	207	195	201	165	165
Aa1814	6	431	431	125	125	205	213	182	182	412	412	253	256	236	236	230	230	205	209	191	195	195	198	170	170
Aa1815	6	427	435	137	139	207	217	182	182	412	412	250	253	242	258	235	235	205	213	191	191	195	198	170	190
Aa1816	6	431	431	137	137	213	213	186	192	412	412	250	250	236	264	230	235	213	213	191	207	198	201	165	170
Aa1817	6	427	427	139	139	217	217	192	198	412	412	250	256	230	238	230	230	209	213	207	207	195	198	175	175
Aa1818	6	427	431	139	139	207	219	182	188	412	412	250	253	238	264	230	230	213	213	207	207	195	195	170	170
Aa1819	6	429	431	137	137	203	227	182	182	412	418	256	256	266	266	230	230	201	209	191	195	195	195	170	170
Aa1820	6	427	429	139	139	205	231	182	192	412	412	250	250	236	236	230	235	213	213	207	207	195	198	170	170
Aa1821	6	431	431	125	125	205	231	182	192	412	412	241	250	238	260	230	235	213	213	191	191	195	195	170	170

Aa1822	6	431	431	165	167	187	205			412	412	256	256	240	280	230	230	201	205	195	207	195	198	190	190
Aa1823	6	431	431	137	137	203	217	198	198	412	412	250	250	236	264	230	230	205	213	191	207	195	198	170	190
Aa1824	6	431	431	125	125	187	205	182	194	412	412	253	253	238	258	230	230	205	209	191	191	195	198	170	170
Aa1825	6	431	431	137	139	217	231	182	206	412	412	253	253	236	236	230	235	205	209	195	195	198	198	185	185
Aa1826	6	427	431	125	137	205	213	194	194	412	412	250	250	238	264	230	230	205	213	191	191	198	201	170	170
Aa1827	6	431	431	125	125	213	221	182	182	412	412	250	259	240	240	230	235	201	213	207	209	198	198	165	170
Aa1828	6	431	431	125	153	213	213	182	182	412	412	244	244	236	264	230	230	209	213	191	191	195	198	170	170
Aa1829	6	431	431	125	125	211	211	184	186	412	412	244	271	248	248	230	235	209	213	207	207	195	198	170	175
Aa1830	6	427	429	137	137	201	219	182	182	412	412	253	256	236	320	230	230	213	213	207	207	195	198	165	175
Aa1831	6	431	431	143	143	201	213	182	194	412	412	256	256	236	268	230	230	205	209	197	197	198	198	175	190
Aa1832	6	429	431	139	139	205	213	182	194	412	418	250	256	236	236	230	230	209	209	191	191	195	198	170	190
Aa1833	6	431	431	125	137	217	231	194	194	412	412	256	256	236	260	230	230	205	213	191	207	195	198	170	190
Aa1834	6	431	431	125	125	201	217	182	182	412	412	253	256	248	268	230	230	209	213	191	191	195	201	175	175
Aa1835	6	431	431	139	139	213	213	182	194	412	412	250	253	230	230	230	230	213	213	207	207	195	195	170	170
Aa1836	6	431	431	137	137	217	219	182	182	412	412	250	256	238	322	235	235	213	217	191	191	195	198	170	175
Aa1837	6	429	429	139	139	205	205	186	194	412	412	250	250	236	236	230	230	205	213	195	207	195	201	170	170
Aa1838	6	425	425	137	137	205	215	184	192	412	412	250	253	238	238	230	230	205	205	191	191	198	198	175	175
Aa1839	6	431	431	137	169	201	213	182	194	412	412	256	256	232	314	230	230	213	213	207	207	195	201	165	175
Aa1840	6	429	431	133	167	211	217	192	192	412	412	250	253	236	314	225	230	213	213	207	207	198	198	170	170
Aa1841	6	427	431	137	137	201	215	182	182	412	412	250	256	314	314	230	230	205	213	191	191	195	198	170	170
Aa1842	6	431	431	133	139	205	217	182	182	412	412	253	256	258	314	230	230	213	213	207	207	195	195	170	170
Aa1843	6	431	431	137	137	207	213	182	182	412	412	256	256	260	260	230	230	205	213	207	207	198	198	190	190
Aa1844	6	429	431	133	133	217	225	182	182	412	412	253	253	228	228	230	235	201	213	207	207	198	201	175	175
Aa1845	6	427	429	137	137	215	231	194	194	412	412	253	256	236	264	230	230	213	213	191	195	195	198	165	175
Aa1846	6	429	429	137	157	205	213	196	196	412	412	256	256	240	258	230	230	209	213	191	207	195	198	190	190
Aa1847	6	431	431	143	143	205	229	194	194	412	412	253	253	228	236	230	230	209	213	189	207	195	195	170	175
Aa1848	6	431	431	125	137	201	229	192	192	412	412	250	250	238	280	230	230	213	213	207	207	195	198	175	175
Aa1849	6	429	431	125	139	187	211	184	184	412	412	250	250	236	236	230	230	209	209	195	195	192	201	170	170
Aa1850	6	431	431	141	159	201	215	192	192	412	412	250	250	238	238	230	235	205	213	195	195	195	198	175	175
Aa1851	6	427	431	137	137	207	217	192	192	412	412	253	256	224	224	235	235	209	213	191	209	195	195	175	175
Aa1852	6	429	431	125	125	207	231	186	196	412	412	256	256	236	236	230	230	209	213	191	209	195	198	185	185
Aa1853	6	427	427	137	137	195	219			412	412	253	256	264	322	230	230	213	213	205	207	198	198	170	170
Aa1854	6	427	429	137	137	205	205	182	182	412	412	253	253	236	260	230	230	205	213	207	207	198	201	170	175
Aa1855	6	431	431	137	137	211	213	194	198	412	418	250	256	240	280	230	230	201	205	191	191	195	198	170	175
Aa1856	6	431	431	137	137	213	213	194	194	412	412	250	250	228	240	235	235	213	213	207	209	195	195	170	170
Aa1857	6	427	433	125	137	217	221	182	182	412	418	250	256	260	264	230	230	209	213	191	195	195	201	170	175
Aa1858	6	431	431	139	139	217	221	182	182	412	412	256	256	240	322	230	230	209	213	191	191	198	201	165	170
Aa1859	6	431	431	139	139	195	217	182	196	412	412	250	253	240	240	230	235	209	213	191	195	195	195	165	170
Aa1860	6	431	431	139	141	191	213	194	194	412	412	253	253	230	230	230	230	209	213	191	195	195	198	170	170
Aa1861	6	429	429	137	137	207	207	194	194	412	418	256	256	248	248	230	230	213	213	191	191	195	201	170	170
Aa1862	6	429	431	137	137	217	217	182	206	412	412	250	256	230	264	230	230	209	209	191	195	195	198	165	170
Aa1863	6	431	431	143	143	211	213	192	194	412	412	253	256	236	236	230	230	213	213	195	205	198	198	170	170
Aa1864	6	427	429			213	213	182	194	412	412	250	250	258	258	230	230	205	209	197	197	195	198	170	170
Aa1865	6	431	431	157	157	201	201	182	182	412	412	250	256	236	236	230	230	213	213	191	191	198	201	170	175
Aa1866	6	429	429	133	139	201	213	182	184	412	412	256	256	236	236	225	230	201	213	191	207	195	198	170	170
Aa1867	6	427	429	137	137	221	231	194	194	412	418	250	256	236	238	230	230	209	213	191	207	201	201	165	175
Aa1868	6	431	431	125	145	217	231	194	194	412	412	250	256	236	236	230	235	213	213	191	191	195	195	170	170
Aa1870	6	431	431	125	137	201	205	184	194	412	412	250	256	264	264	230	230	201	205	197	197	195	198	170	170
Aa1879	7	427	427	139	139	201	211	182	194	412	412	250	250	238	322	230	230	201	213	191	191	198	201	170	170
Aa1880	7	429	429	137	137	201	205	184	192	412	418	253	256	236	238	235	235	205	209	191	207	195	195	175	180
Aa1881	7	431	431			205	221	182	182	412	412	250	256	264	286	230	230	209	209	191	209	198	198	175	190
Aa1882	7	427	429	137	137	201	205	184	184	412	412	244	256	236	238	225	235	209	213	191	191	195	198	190	190
Aa1883	7	431	431	125	137	213	217	182	182	412	412	250	253	236	236	230	230	213	213	191	197	195	198	190	190
Aa1884	7	429	433	125	135	199	201	202	202	412	412	256	256	236	236	230	230	213	213	191	195	195	195	170	170
Aa1885	7	431	431	137	137	201	201	192	192	412	412	253	253	260	260	235	235	213	213	207	207	195	201	175	190
Aa1886	7	431	431	139	143	201	217	194	194	412	412			248	260	230	230	217	217	195	207	195	198	165	175
Aa1887	7	427	431	133	143	201	213	182	182	412	412	253	253	270	270	235	235	209	213	197	207	201	201	170	175
Aa1888	7	431	431	125	139	205	215	192	192	412	418	253	253	280	280	230	230	209	217	207	207	198	201	175	180
Aa1889	7	431	431	143	169	201	217	196	202	412	412	250	250	236	236	230	230	209	213	191	191	198	201	170	175
Aa1890	7	431	431	139	139	197	199	182	182	412	412	250	250	264	264	230	230	205	209	191	207	195	195	170	175
Aa1891	7	431	431	139	155	211	231	192	198	412	412	250	250	236	236	230	230	209	213	191	195	198	201	170	170
Aa1892	7	431	431	137	141	187	223	182	184	412	412	250	250	236	248	230	230	213	213	191	207	195	198	175	175
Aa1893	7	429	431	133	137	203	221	182	202	412	418	256	256	236	238	230	230	209	213	191	191	195	198	170	175
Aa1894	7	427	429	139	139	211	211	182	184	412	412	250	256	236	260	230	235	213	213	207	207	195	198	170	170
Aa1895	7	429	429	137	165	207	217	194	204	412	418	253	256	236	236	230	235	201	213	191	191	195	195	170	170
Aa1896	7	431	431	137	143	203	231	192	206	412	412	250	256	236	248	230	230	201	213	191	197	198	198	170	175
Aa1897	7	429	431	135	135	201	223	206	206	412	412	250	250	322	322	230	230	209	213	191	191	195	198	175	190
Aa1898	7	427	431	139	143	211	231	192	198	412	412	250	256	236	264	230	230	193	213	195	209	195	201	170	175
Aa1899	7	429	431	157	159	195	231	182	202	412	412	253	256	236	248	230	235	213	213	207	207	195	198	170	170
Aa1900	7	429	431	137	155	199	231	192	194	412	412	253	253	260	268	230	230	201	213	191	191	195	198	170	170
Aa1901	7	429	431	137	137	217	223	182	182	412	412	256	256	236	258	235	235	205	213	207	207	195	195	170	175
Aa1902	7	427	427	125	125	201	201	206	206	412	412	250	250	236	260	230	230	205	209	207	207	201	201	170	170
Aa1903	7	429	431	137	137	201	231	184	186	412	418	250	256	228	236	230	230	201	201	191	207	195	195	170	170
Aa1904	7	427	427	155	155	199	201	182	182	412	412	250	250	238	238	230	230	209	213	207	207	195	198	170	190
Aa1905	7	429	429	139	139	201	219	182	194	412	412	253	256	238	260	230	235	213	213	191	191	195	198	170	175
Aa1906	7	431	431	155	155	205	213	194	196	412	412	256	256	238	260	230	235	201	201	197	197	195	195	170	190
Aa1907	7	427	429	137	137	213	213	182	182	412	412	256	256	264	270	235	235	205	213	207	207	195	195	170	175
Aa1908	7	427	427	125	137	213	217	184	184	412	412	253	256	230	238	230	230	193	209	191	207	195	195	165	165

Aa1909	7	429	429	133	137	201	221	194	204	412	412	250	250	236	264	230	230	213	217	207	207	195	195	165	180
Aa1910	7	427	431	125	165	197	197	194	194	412	412	250	256	264	264	230	230	209	213	207	207	195	198	190	190
Aa1911	7	427	431	137	137	203	217	186	202	412	418	256	256	236	264	230	230	205	205	205	207	195	198	165	165
Aa1912	7	431	431	133	137	203	205	182	192	412	412	250	250	240	280	230	230	205	209	195	195	195	195	175	175
Aa1913	7	427	427	125	125	195	231	184	194	412	412	250	250	236	236	230	230	201	213	195	207	195	198	165	170
Aa1914	7	431	431	137	137	201	205	182	182	412	412	250	250	238	264	230	235	201	213	195	207	198	198	175	175
Aa1915	7	427	429	135	135	221	231	186	186	412	412	256	256	248	270			205	205	195	195	198	198	170	175
Aa1916	7	427	431	137	137	187	217	182	182	412	418	250	253	230	264	230	230	209	209	207	207	195	198	175	175
Aa1917	7	427	431	133	137	201	201	180	204	412	412	250	256	248	270	230	230	213	213	191	207	195	201	170	190
Aa1918	7	429	431	137	137	205	213	192	192	412	412	250	250	238	322	230	230	205	209	191	207	195	195	170	175
Aa1919	7	431	431	137	137	219	221	194	194	412	412	250	250	230	230	230	230	213	213	191	191	195	195	170	170
Aa1920	7	429	431	137	137	201	217	182	184	412	412	250	250	236	264	230	230	205	213	191	195	195	198	165	170
Aa1921	7	427	429	125	143	199	219	182	184	412	412	250	256	228	236	230	230	201	205	191	207	195	195	165	165
Aa1922	7	431	431	137	157	205	221	194	194	412	412	256	256	238	238	235	235	205	213	191	195	195	198	170	170
Aa1923	7	431	431	139	139	219	237	182	182	412	412	256	256	260	260	230	230	201	213	191	207	195	198	175	175
Aa1924	7	431	431	149	149	201	219	194	194	412	412	250	250	230	238	230	230	205	213	189	207	198	201	170	175
Aa1925	7	431	431	125	137	191	217	186	186	412	412	256	256	238	238	230	230	209	213	207	207	195	195	165	170
Aa1926	7	427	429	137	137	195	227	182	194	412	412	250	253	236	264	230	230	201	201	207	207	198	201	175	190
Aa1927	7	431	431	137	137	201	203	182	182	412	412	244	250	236	240	230	230	205	213	197	197	195	195	165	165
Aa1928	7	431	431	139	139	197	215	194	204	412	412	250	256	236	238	230	230	209	209	191	207	195	198	170	170
Aa1929	7	431	431	139	165	201	201	184	206	412	412	253	256			230	230	201	209	191	191	195	195	165	170
Aa1930	7	431	431	125	125	195	223	182	182	412	412	256	256	230	236	225	230	213	213	191	191	195	201	170	170
Aa1931	7	431	431	143	169	207	213	180	180	412	412	256	256	232	322	230	230	205	209	191	191	198	198	170	170
Aa1932	7	427	429	137	137	205	205	194	206	412	418	250	256	236	236	230	230	213	213	195	195	195	198	170	175
Aa1933	7	431	433			205	213	182	182	412	412	253	256	236	260	230	230	209	213	191	191	195	201	175	180
Aa1934	7	429	431	137	153	211	217	182	182	412	412	250	250	236	236			209	213	207	207	195	195	170	170
Aa1935	7	427	431	125	137	205	217	184	194	412	412	250	250	236	236	230	230	205	213	191	191	195	198	170	175
Aa1936	7	427	431	137	137	207	217	182	182	412	418	241	241	238	238	230	230	209	213	207	207	195	201	170	170
Aa1937	7	427	431	165	167	211	217	182	182	412	412	250	250	260	260	230	230	205	213	195	207	195	195	170	170
Aa1938	7	429	431	137	137	221	231	182	182	412	412	256	271	238	264	235	235	213	213	207	209	195	201	170	170
Aa1939	7	431	431	135	167	213	231	184	184	412	412	250	256	236	236	230	230	213	213	207	207	195	198	190	190
Aa1940	7	431	431	135	137	183	213	192	192	412	412	250	253	280	314	230	230	205	205	193	197	195	195	180	190
Aa1941	7	431	431	125	137	203	213	182	206	412	412	241	250	242	242	230	230	205	205	207	207	195	198	170	180
Aa1942	7	427	427	135	135	205	213	182	182	412	412	256	256	236	236	230	235	205	213	207	207	195	201	165	170
Aa1943	7	427	431	137	165	195	211	184	202	412	412	250	256	236	236	230	230	213	213	191	191	195	198	170	170
Aa1944	7	431	431	125	125	217	217	182	182	412	412	250	253	236	236	230	230	213	213	191	195	195	198	175	175
Aa1945	7	427	431	133	155	201	201	186	194	412	412	250	256	240	240	230	230	213	213	191	207	195	201	170	170
Aa1946	7	431	431	125	137	217	231	182	182	412	412	253	256	236	264	230	230	213	213	191	191	198	201	165	165
Aa1947	7	431	431	125	143	217	217	182	182	412	412	250	256	236	266	230	230	213	213	191	191	195	198	165	175
Aa1948	7	431	431	137	137	199	201	182	182	412	412	250	256	236	236	230	230	213	213	191	191	195	198	170	170
Aa1949	7	427	431	125	125	201	213	182	182	412	412	256	256	240	248	230	230	205	213	191	207	195	201	170	170
Aa1950	7	429	429	137	137	203	213	194	194	412	412	250	256	236	258	230	230	213	217	207	207	195	198	170	170
Aa1951	7	427	431	155	155	197	211	192	194	412	412	244	250	260	264	230	230	213	213	195	207	195	198	170	175
Aa1952	7	431	431	137	165	205	227	182	192	412	412	250	256	236	236	230	230	213	213	191	205	195	198	190	190
Aa1953	7	429	431			191	219	182	182	412	412	250	256	258	260	235	235	205	213	207	207	195	198	165	170
Aa1954	7	427	431	137	165	221	225	192	192	412	412	250	250	240	322	230	230	209	213	197	197	195	201	175	190
Aa1955	7	427	431	137	137	213	227	182	182	412	412	259	259	260	260	230	230	205	209	207	209	198	198	190	190
Aa1956	7	429	429	125	125	217	217			412	412	253	256	230	236	230	230	201	213	191	207	195	198	165	170
Aa1957	7	427	431	141	141	217	231	194	202	412	412	256	256	230	236	230	230	205	205	191	191	195	201	165	190
Aa1958	7	431	431			207	217	194	194	412	412	253	256	236	264	230	230	201	209	191	209	195	195	175	175
Aa1959	7	431	431	133	133	227	231	182	182	412	412	253	256	238	266	230	230	209	213	207	207	195	198	175	190
Aa1960	7	427	429	137	137	217	221	182	194	412	412	256	256	230	236	230	230	209	213	195	207	195	198	170	170
Aa1961	7	431	431	125	125	197	221	182	184	412	412	256	256	236	236	230	230	209	213	191	209	195	198	170	185
Aa1962	7	425	427	137	167	201	219	182	192	412	412	253	256	236	236	230	230	213	213	195	207	198	198	170	175
Aa1963	7	427	429			227	231	182	182	412	418	250	253	236	236			205	213	195	207	195	201	190	190
Aa1964	7	431	431	137	137	195	213	184	206	412	412	253	253	238	238	230	230	205	213	197	197	195	201	165	165
Aa1965	7	427	431	125	139	201	217	182	194	412	412	253	274	236	236	230	230	205	213	191	191	195	198	170	175
Aa1966	7	431	431	125	145	213	217	186	208	412	418	253	256	248	260	230	230	205	213	197	209	195	201	170	180
Aa1967	7	431	431	139	141	201	211	182	194	412	418	253	256	322	322	230	230	193	213	207	207	195	198	175	175
Aa1968	7	431	431	141	141	211	217	184	184	412	412	253	256	236	236	230	230	205	205	191	207	195	195	170	170
Aa1969	7	429	429	139	139	201	213	182	206	412	418	253	256	248	248			205	209	191	193	195	201	170	190
Aa1970	7	429	431	137	137	203	217	194	194	412	412	253	253	238	238	230	230	213	213	207	207	189	195	170	170
Aa1971	7	429	431	137	137	207	207	206	206	412	418	250	253	236	258	230	235	209	209	197	197	195	198	175	175
Aa1972	7	429	429	125	125	197	201	194	202	412	412	253	256	226	314	230	230	205	209	191	195	195	195	170	170
Aa1974	7	427	427	137	137	199	217	182	182	412	412	253	256	236	236	230	230	213	213	207	207	195	198	170	170
Aa1976	7	431	431	137	137	231	231	182	192	412	412	250	250	236	322	230	230	201	209	197	207	195	198	170	170
Aa1977	7	427	427			225	231	184	184	412	412	250	256	260	264	230	230	205	205	195	207	195	198	170	175
Aa1978	7	427	429	137	137	217	231	182	194	412	412	256	256	228	228	230	230	213	213	207	207	195	195	170	170
Aa3735	8	429	429	125	143	201	215	186	192	412	412	256	256	238	238	230	230	213	213	191	207	195	198	170	175
Aa3736	8	429	431	137	137	199	213	184	184	412	412	253	253	236	238	230	230	213	213	207	207	201	201	190	190
Aa3737	8	427	429	139	139	195	221	192	198	412	412	244	253	236	236	235	235	201	205	191	209	195	198	170	175
Aa3738	8	431	433	137	155	187	199	182	182	412	418	250	250	248	258	230	230	205	213	207	207	195	195	170	170
Aa3739	8	427	427	125	125	213	221	182	182	412	412	244	256	236	264	230	235	201	209	191	191	195	195	170	170
Aa3740	8	427	427	125	125	187	211	192	196	412	412	250	250	236	264	230	230	205	205	195	209	195	195	170	175
Aa3741	8	431	431	137	139	207	221	182	182	412	412	241	253	232	236	235	235	213	213	205	205	198	201	170	170
Aa3742	8	427	431	125	139	201	231	204	204	412	412	253	253	240	260	230	230	213	213	207	207	195	198	175	175
Aa3743	8	427	431	139	169	221	231	182	194	412	412	250	256	236	248	235	235	205	213	191	207	195	201	170	170
Aa3744	8	429	429	135	135	201	201	184	198	412	418	256	256	230	236	235	235	213	213	191	207	195	198	175	190

Aa3745	8	429	429	157	157	187	203	182	194	412	412	250	256	236	236	230	230	213	213	191	207	195	195	170	190
Aa3746	8	431	431	135	137					412	412					230	230	205	209	191	197	198	201	175	175
Aa3747	8	429	431	139	169	205	217	182	182	412	412	250	256	236	266	235	235	201	209	195	207	195	198	175	175
Aa3748	8	431	431	137	137	201	203	184	184	412	412	250	250	236	236	230	230	205	213	191	195	195	198	170	170
Aa3749	8	425	425	125	125	201	219	194	194	412	412	250	250	236	236	230	230	209	213	191	207	195	195	175	175
Aa3750	8	427	431	135	135	213	231	184	206	412	412	253	256	264	264	230	230	205	213	191	191	198	198	180	180
Aa3751	8	427	427	157	169	205	217	184	184	412	418	253	253	236	238	230	230	213	217	207	207	195	198	170	170
Aa3752	8	427	431	137	137	217	221	182	182	412	418	253	256	236	236	230	230	205	213	191	207	198	198	170	170
Aa3753	8	431	431	125	137	213	217	186	194	412	418	250	256	236	322	230	230	209	213	207	209	195	201	170	170
Aa3754	8	431	431			205	231	182	198	412	412	250	256	236	238	230	230	213	213	195	195	195	195	170	170
Aa3755	8	427	431			201	205	184	184	412	412	256	256	322	322	230	235	205	213	207	209	195	201	170	175
Aa3756	8	429	433	133	155	213	213	182	182	412	412	253	253	236	236			213	213	191	207	195	198	170	170
Aa3757	8	431	431	125	137	219	223	182	206	412	412	250	253	238	264	230	230	213	213	207	207	195	195	170	170
Aa3758	8	431	431	137	137	201	219	170	182	412	412	256	256	236	236			213	217	207	207	195	195	170	170
Aa3759	8	429	429	137	137	213	213			412	412	256	256	228	240	230	235	201	213	191	207	195	195	170	170
Aa3760	8	431	431	133	139	203	217	194	194	412	412	256	256	236	236	230	230	209	209	191	207	195	201	170	170
Aa3761	8	427	431	125	125	195	219	194	194	412	412	250	250	258	258	230	230	213	217	191	207	195	198	170	170
Aa3762	8	431	431	125	137	201	203	206	206	412	412	253	253	238	238	230	230	213	217	191	191	195	198	170	170
Aa3763	8	431	431	137	139	205	217	186	194	412	412	250	256	236	236	230	230	209	213	195	207	198	198	170	170
Aa3764	8	431	431	125	133	201	217	182	182	412	412	253	256	258	258	230	230	205	209	191	195	195	198	165	170
Aa3765	8	431	431	133	139	213	243	184	188	412	418	253	256	236	236			213	213	207	207	195	198	165	165
Aa3766	8	427	431	137	137	187	201	182	192	412	412	256	256	236	236	230	230	209	217	191	207	195	201	165	170
Aa3767	8	427	431	125	125	201	213	192	192	412	412	256	256	238	238	230	230	201	201	191	195	198	204	170	170
Aa3768	8	427	431	137	137	201	215	182	184	412	412	250	253	236	238	230	235	209	213	207	207	195	198	170	175
Aa3769	8	427	431	135	135	187	201	194	194	412	412	256	256	238	248	225	225	205	213	191	207	201	201	165	170
Aa3771	8	429	431	137	137	187	221	182	194	412	412	256	259	260	260	230	230	213	213	207	207	195	195	170	175
Aa3772	8	427	427	125	137	217	223	182	182	412	412	250	256	236	322	230	230	213	213	189	205	195	198	170	170
Aa3773	8	427	431	137	143	205	217	192	208	412	412	250	253	238	248	230	230	213	213	207	207	195	201	170	175
Aa3774	8	431	431	137	137	205	217	194	206	412	412	244	250	236	240	230	230	205	213	195	207	195	195	170	170
Aa3775	8	427	431	125	137	213	221	196	196	412	412	250	256	232	236	230	230	213	213	195	195	195	198	170	170
Aa3776	8	427	431	125	137	205	223	182	184	412	412	253	253	236	240	230	235	205	205	191	191	195	198	170	175
Aa3777	8	429	431	137	137	213	217	182	186	412	418	253	256	236	236	230	230	209	213	191	207	201	201	170	170
Aa3778	8	427	431	125	125	213	223	182	182	412	412	250	256	230	320			209	213	197	197	195	198	170	175
Aa3779	8	427	431	137	137	199	231	192	194	412	412	241	256	230	264	230	230	213	213	191	191	195	195	175	175
Aa3780	8	427	431	125	125	201	203	170	182	412	412	256	256	236	236	230	230	205	209	197	207	195	198	170	190
Aa3781	8	431	431	137	137	201	205	184	184	412	412	253	256	228	228	230	230	193	213	207	207	198	201	175	175
Aa3782	8	429	431	125	137	191	213	194	202	412	412	253	271	224	224	230	230	213	213	207	207	195	198	175	175
Aa3783	8	431	431	133	133	205	205	194	204	412	412	244	253	238	268	225	225	213	213	191	207	195	195	170	170
Aa3784	8	431	431	125	151	201	231	184	194	412	412	256	256	236	236	225	225	213	213	195	195	195	198	165	170
Aa3785	8	429	431	139	155	217	227	182	194	412	412	250	256	240	264	230	230	205	213	191	191	195	195	170	175
Aa3786	8	431	431	137	137	209	209	198	198	412	412	256	256	238	280	230	235	209	213	207	207	198	198	185	190
Aa3787	8	427	427	125	125	201	231	182	182	412	412	250	256	236	238	230	230	205	205	195	207	198	198	170	175
Aa3788	8	429	431	123	125	199	221			412	412	256	256	236	240	230	230	213	213	191	191	198	198	185	190
Aa3789	8	431	431	137	139	213	219	182	206	412	412	256	256	236	236	225	230	201	201	191	191	195	198	190	190
Aa3790	8	427	431	137	137	201	201	182	194	412	412	253	253	232	236	230	230	201	213	205	207	195	198	175	175
Aa3791	8	431	431	137	137	217	217	182	192	412	412	271	271	236	264	230	230	213	213	191	195	195	195	165	165
Aa3792	8	431	431	135	155	211	231	182	182	412	412	253	256	236	236	230	230	201	213	191	207	195	198	165	170
Aa3793	8	427	431	125	125	199	213	192	194	412	418	253	253	236	280	230	230	201	213	195	195	195	198	170	175
Aa3794	8	429	429	137	165	201	203	182	182	412	412	253	253	236	242	225	235	213	213	195	195	195	198	170	170
Aa3795	8	429	431	139	165	213	213	182	186	412	412	256	256	236	322	230	230	209	213	197	197	195	195	170	170
Aa3796	8	429	431	137	137	201	213	182	182	412	412	250	253	268	268	230	235	201	213	191	207	195	195	170	170
Aa3797	8	431	431	137	139	213	217	182	206	412	412	250	256	230	230	230	230	205	205	195	207	195	198	165	165
Aa3798	8	427	427	137	155	201	231	182	206	412	412	253	253	264	274	235	235	213	213	191	191	195	198	170	175
Aa3799	8	427	427	125	125	187	201	182	182	412	412	256	256	232	270	230	230	209	213	207	207	195	198	170	175
Aa3800	8	429	431	137	137	219	221	194	194	412	418	250	256	236	236			209	213	191	209	195	201	170	170
Aa3801	8	431	431	137	137	201	213	192	192	412	412	250	256	224	236	230	235	209	213	207	207	195	195	170	170
Aa3802	8	427	427	135	159	201	205	182	192	412	412			236	266	230	235	205	213	195	205	195	198	170	170
Aa3803	8	427	431	137	149	203	221	192	194	412	412	250	253	236	260	230	230	205	213	191	207	195	198	165	170
Aa3804	8	431	431	125	125	213	217	182	202	412	412	250	250	236	240			213	213	207	207	195	195	170	175
Aa3805	8	431	431	133	133	195	217	194	206	412	412	256	256	236	236	230	230	209	217	191	209	195	198	170	170
Aa3806	8	431	431	125	133	199	199	182	194	412	412	250	250	240	280	230	230	213	213	189	209	198	198	190	190
Aa3807	8	429	429			205	229	182	184	412	412	250	250	236	238	230	230	213	213	193	207	195	195	190	190
Aa3808	8	431	431	141	141	187	231	182	206	412	412	250	259	236	236	230	230	213	213	191	207	195	198	170	170
Aa3809	8	427	431	125	125	201	205	182	182	412	412			236	240	230	230	205	213	191	207	195	195	165	165
Aa3810	8	429	429	155	155	205	215	188	188	412	412	253	256	236	238	230	230	201	209	191	191	198	198	170	170
Aa3811	8	427	431	137	137	201	203	182	182	412	412	256	256	236	236	230	230	213	213	197	207	198	198	170	170
Aa3812	8	431	431	125	125	215	217	206	206	412	412	250	256	236	260	225	225	213	213	195	197	195	195	170	170
Aa3813	8	427	431	125	137	195	217	192	194	412	412	256	256	236	236	230	230	201	201	207	207	195	195	170	170
Aa3814	8	429	429	135	135	197	201	192	194	412	412	250	256	236	236	230	230	213	213	189	209	195	195	175	175
Aa3815	8	425	431	125	137	205	219	194	194	412	412	250	250	238	248	230	230	205	213	191	207	195	195	175	175
Aa3816	8	427	431	137	137	201	213	182	182	412	412	241	250	236	250	235	235	205	213	191	207	195	198	170	190
Aa3817	8	425	427	143	143	205	217	182	194	412	412	250	250	236	236	230	230	201	205	207	207	195	198	165	165
Aa3818	8	429	431	125	125	213	217	182	182	412	412	250	256	236	264	230	230	209	209	197	197	198	201	170	170
Aa3819	8	431	431	125	125			182	182	412	412	250	253	236	236	230	230			191	191	195	204	165	175
Aa3820	8	425	431	125	137	205	217	194	194	412	412	253	253	228	236	230	230	213	213	191	207	195	201	165	170
Aa3821	8	427	431	137	143	189	217	194	194	412	412	250	253	238	266	230	235	201	205	189	207	198	201	170	175
Aa3822	8	427	429	137	139	199	207	194	194	412	412	253	253	248	264	230	235	209	213	191	191	195	195	165	175
Aa3823	8	431	433			201	211	182	182	412	412	253	253	232	232	230	230	213	213	191	207	195	195		

Aa3824	8	431	431	139	167	195	213	182	194	412	418	256	256	236	264	230	235	213	217	207	207	198	198	170	175
Aa3825	8	429	431	133	139	211	221	182	182	412	412	256	256	224	264	230	230	213	213	191	195	195	195	190	190
Aa3826	8	427	431	135	135	197	217	182	206	412	418	250	250	236	236	230	230	201	209	207	207	195	195	170	170
Aa3827	8	429	431	125	137	205	219	192	192	412	412	253	274	236	236	230	230	209	213	197	207	195	198	170	175
Aa3828	8	431	431	137	137	201	231	194	194	412	412	241	271	248	248	230	235	205	213	191	191	195	198	165	175
Aa3829	8	431	431	137	137	205	221	186	194	412	418	250	256	232	264	230	230	205	213	195	207	195	195	190	190
Aa3830	8	431	431	133	137	223	223	182	182	412	412	250	250	236	236	230	230	213	213	191	207	198	201	190	190
Aa3831	8	427	431	125	149	217	217	182	182	412	412	250	262	236	264	230	230	205	213	191	193	195	204	170	170
Aa3832	8	427	427	137	137	191	217	182	182	412	412	253	253	236	240	230	230	213	213	207	209	198	201	170	170
Aa3837	9	429	429			217	231	182	194	412	412	253	256	238	264			205	213	207	207	195	201	170	190
Aa3838	9	431	431	137	137	197	211	184	206	412	412	256	256	236	236			205	209	197	197	198	198	170	170
Aa3839	9	427	427	139	139	207	217			412	412	256	256	236	248	235	235	201	201	191	191	192	198	170	170
Aa3840	9	431	431	137	137	187	219	182	184	412	412	250	256	236	236			209	213	197	207	195	198	170	170
Aa3841	9	429	431	125	139	201	205	192	192	412	418	256	256	236	236	230	230	209	213	207	207	195	195	165	175
Aa3842	9	429	431	137	137	197	221	192	204	412	412	250	253	236	314			205	213	207	207	195	198	175	175
Aa3843	9	431	431	137	141	219	227	182	184	412	412	256	256	264	320	230	230	213	213	207	207	195	207	165	170
Aa3844	9	427	431	139	139	201	205	184	198	412	412	241	250	236	236	230	230	213	213	191	207	192	195	170	170
Aa3845	9	427	431	133	133	199	211	182	194	412	412	253	256	236	240	230	230	201	205	191	191	195	198	175	175
Aa3846	9	431	431	137	169	201	205	184	184	412	412	250	274	248	264	230	230	213	213	195	195	195	198	170	175
Aa3847	9	427	431	137	139	197	201	182	194	412	412	241	250	236	236	230	230	201	213	207	207	195	198	170	170
Aa3848	9	427	431	137	137	205	219	194	204	412	412	250	250	240	240	235	235	205	213	191	197	195	201	170	170
Aa3849	9	429	429	165	167	213	231	184	206	412	412	256	256	320	320	230	235	209	209	207	209	195	195	175	190
Aa3850	9	427	427	125	125	187	199	182	182	412	412	253	253	238	280	230	230	205	213	191	207	198	198	190	190
Aa3851	9	431	431	125	125	201	217	194	204	412	412	250	256	228	238	230	230	205	209	191	207	195	198	170	170
Aa3852	9	427	431	135	139	195	201	182	194	412	412	250	253	266	266	230	230	205	205	207	207	195	195	170	170
Aa3853	9	431	431	137	137	213	227	182	204	412	412	250	253	236	236	230	230	213	213	191	207	195	198	170	170
Aa3854	9	427	427	125	167	201	201	194	194	412	412	256	256	238	238	230	230	205	209	207	207	195	198	170	190
Aa3855	9	429	429	125	125	211	219	182	182	412	412	253	253	224	238	230	230	201	213	207	207	195	198	165	175
Aa3856	9	429	431	165	165	195	201	182	194	412	418	250	250	236	322	230	230	205	217	191	191	195	201	175	175
Aa3857	9	427	427	125	137	187	201	192	198	412	412	253	253	236	236	230	230	201	213	191	191	198	201	170	175
Aa3858	9	427	431	125	165	217	235	186	192	412	412	250	256	236	236	235	235	213	213	195	195	195	195	190	190
Aa3859	9	427	429	137	137	217	231	182	206	412	412	253	256	238	264	230	230	209	213	191	207	195	198	170	170
Aa3860	9	429	431	137	137	217	221	182	204	412	412	250	250	264	264	230	230	213	213	207	207	198	201	165	165
Aa3861	9	431	431	123	139	213	231	194	202	412	412	250	256	236	322	230	230	213	213	207	207	195	195	170	170
Aa3862	9	431	431	137	159	207	221	182	186	412	412	256	256	248	280	230	235	209	209	189	207	195	195	170	175
Aa3863	9	427	429	137	137	205	217	182	182	412	412	250	250	236	248	230	235	205	209	191	207	195	201	175	175
Aa3864	9	429	431	125	137	217	237	182	182	412	412	250	256	230	238	230	230	205	213	197	207	195	195	170	170
Aa3865	9	429	429	139	139	199	205	182	182	412	412	256	256	236	260	230	230	201	205	207	207	198	204	165	165
Aa3866	9	429	431	125	153	217	219	194	194	412	412	274	274	232	322	230	230	213	213	207	209	195	198	170	175
Aa3867	9	427	431	135	157	231	231	194	198			250	256	236	236	230	235	213	213	193	193	195	198	165	190
Aa3868	9	431	431	133	133	217	223	182	182	412	412	253	253	236	238	230	230	205	213	191	191	198	201	170	170
Aa3869	9	431	431	125	159	201	205	182	206	412	412	250	250	236	258	230	235	205	217	207	207	198	201	170	170
Aa3870	9	429	429	125	125	203	217	182	182	412	412	250	250	236	238	235	235	205	209	191	195	195	198	170	175
Aa3871	9	431	431	137	137	201	217	194	194	412	412	256	256	238	238	230	230	213	213	191	207	195	201	170	175
Aa3872	9	429	431			201	221	192	192	412	412	253	253	260	260			205	205	197	197	195	198	170	175
Aa3873	9	427	431	137	137	205	221	184	202	412	418	250	256	238	238	230	230	209	217	191	191	195	195	165	170
Aa3874	9	429	433	137	137	191	211	182	194	412	412	250	250	238	264	230	230	201	213	207	207	195	195	170	170
Aa3875	9	431	431	125	137	205	227	194	206	412	418	256	256	230	238	230	230	213	213	207	207	195	198	170	170
Aa3876	9	431	431	125	149	231	231	184	192	412	412	253	256	236	238			213	213	191	207	198	198	170	170
Aa3877	9	431	431	125	135	187	213	188	188	412	412	250	256	230	236	230	230	209	213	207	207	195	198	170	175
Aa3878	9	427	429	141	141	205	231	182	194	412	412	253	253	280	280	230	230	209	217	207	207	195	195	170	190
Aa3879	9	427	431	125	137	217	221	192	192	412	412	250	256	236	238	230	235	213	213	197	197	195	198	170	170
Aa3880	9	431	433	155	157	201	213	182	192	412	412	250	256	238	238	230	230	213	213	191	197	195	198	170	170
Aa3881	9	431	431	139	139	211	217	194	194	412	412	250	256	228	236	230	230	201	213	205	207	195	201	170	170
Aa3882	9	431	431	125	125	199	201	184	184	412	412	250	256	264	264	230	235	209	213	195	207	195	198	175	175
Aa3883	9	431	431	125	125	215	217	194	206	412	412	253	256	236	236	230	230	205	209	191	195	198	198	170	170
Aa3884	9	431	431	137	137	207	207	182	194	412	418	250	253	236	264	230	230	205	213	191	207	195	201	165	165
Aa3885	9	427	431	167	169	197	217			412	412	250	256	230	236	230	230	201	213	193	195	195	198	170	170
Aa3886	9	431	431	137	137	191	213			412	412	250	250	226	238	230	230	193	213	191	191	195	195	170	170
Aa3887	9	431	431	139	169	201	221	184	184	412	412	250	256	236	236	230	230	213	217	191	207	195	204	190	190
Aa3888	9	431	431	133	159	205	223	182	184	412	418	256	256	236	260	235	235	209	213	207	209	195	198	170	170
Aa3889	9	431	431	135	135	201	203	182	182	412	412	256	256	238	238	230	230	213	213	191	195	198	201	170	175
Aa3890	9	429	429	125	137	191	213	182	182	412	412	250	250	230	238	230	230	209	213	191	191	195	198	170	170
Aa3891	9	431	431	137	167	201	203	194	196	412	412	256	259	238	260	230	230	213	213	195	195	198	198	165	165
Aa3892	9	427	431	125	137	211	221	182	182	412	412	256	256	236	266	230	230	213	213	191	191	195	198	165	165
Aa3893	9	427	435	125	139	203	203	192	194	412	412	256	256	286	286	230	235	205	213	191	191	198	198	190	190
Aa3894	9	429	431	143	143	217	221	182	194	412	412	253	253	264	322	230	235	213	213	207	207	195	201	170	170
Aa3895	9	429	431	139	139	205	221	182	192	412	412	250	253	236	270	230	230	201	213	195	195	195	195	170	170
Aa3896	9	431	431	137	137	187	227	182	182	412	418	241	256	236	322	230	235	213	213	195	195	195	198	165	180
Aa3897	9	431	433	125	165	205	217	194	194	412	412	256	256	236	280	230	230	213	217	207	207	195	198	165	175
Aa3898	9	431	431	137	139	187	221	194	194	412	412	256	274	236	236	230	230	201	205	197	207	192	195	175	190
Aa3899	9	427	427	125	125	213	213	182	206	412	412	250	259	236	260	230	235	213	213	191	195	195	198	170	170
Aa3900	9	431	431	125	139	199	213	194	194	412	412	253	256	230	230	230	230	201	209	195	207	195	198	170	170
Aa3901	9	431	431	133	167	201	213			412	412	250	253	236	238	230	230	205	213	191	191	195	198	170	175
Aa3902	9	431	431	137	139	191	217	182	182	412	412	241	256	236	264	230	230	209	213	207	207	195	201	170	170
Aa3903	9	431	431	139	139	195	213	192	194	412	412	256	256	238	238	230	230	213	213	207	207	198	198	170	170
Aa3904	9	429	431	137	139	199	213			412	418	253	256	236	268			205	205	207	207	195	198	170	170
Aa3905	9	431	431	137	153	213	219	182	196	412	418	253	253	236	236	230	230	209	213	195	207	195	198	190	190

Aa3906	9	427	431	125	137	213	221	182	184	412	412	253	256	236	238	230	230	201	213	191	207	195	201	170	175
Aa3907	9	427	431	137	137	201	213			412	412	250	253	264	264	230	230	209	213	191	191	195	198	170	175
Aa3908	9	429	431			201	207	186	204	412	412	253	256	236	236	230	235	213	213	191	207	195	198	170	170
Aa3909	9	431	431	125	125	207	231	182	194	412	412	256	256	238	264	230	230	213	213	191	191	195	201	170	170
Aa3910	9	429	429	137	143	213	243	182	184	412	418	250	256	236	236	230	230	205	209	191	195	195	198	175	175
Aa3911	9	431	431	125	125	187	203	182	206	412	418	253	256	236	236	230	230	209	213	191	197	195	195	170	175
Aa3912	9	431	431	137	137	205	213	194	194			253	256	236	236	230	235	209	213	207	207	195	195	170	170
Aa3913	9	427	429	135	135	213	213	194	194	412	412	250	253	236	264	235	235	205	213	191	207	195	198	170	175
Aa3914	9	431	431	125	135	201	217	182	192	412	412	256	256	236	264	230	230	201	209	207	207	195	195	170	170
Aa3915	9	427	431	139	141	199	217	182	182	412	418	253	253	236	258	230	230	205	209	191	191	195	195	170	170
Aa3916	9	427	427	139	139	187	213	182	194	412	412	256	256	236	270	230	230	213	213	191	207	195	201	175	175
Aa3917	9	429	431	137	167	201	201	182	192	412	412	256	256	238	248	230	230	213	213	191	209	195	195	170	175
Aa3918	9	431	431	125	137	211	221	194	194	412	412	250	250	230	236	230	235	213	213	195	195	195	195	170	175
Aa3919	9	427	433	137	139	211	231	182	198	412	412	250	250	236	236	230	230	205	209	189	207	195	198	170	190
Aa3920	9	427	431	125	137	201	201	194	194	412	412	250	256	236	264	230	230	213	213	191	191	195	195	175	175
Aa3921	9	431	431	137	137	201	243	186	206	412	412	256	256	238	238	230	230	213	213	191	197	195	201	170	175
Aa3922	9	427	431			213	219	182	194	412	412	250	256	236	314	230	235	205	209	191	205	201	201	170	170
Aa3923	9	431	431	137	137	201	213	182	182	412	412	250	250	268	268	230	230	205	209	207	207	195	195	165	170
Aa3924	9	431	431	137	165	213	217	182	182	412	412	250	256	238	258	230	230	213	213	191	207	195	198	165	175
Aa3925	9	431	431	125	137	213	217	192	194	412	412	253	256	236	238	230	230	213	213	207	207	198	198	170	175
Aa3926	9	431	431	137	137	217	217	182	182	412	418	250	256	236	264	230	230	213	213	191	207	198	198	165	170
Aa3928	9	431	431	137	137	201	211	206	206	412	418	250	256	236	236	235	235	205	209	195	197	195	198	170	170
Aa3929	9	427	429	137	137	201	221	182	202	412	412	256	256	236	236	230	230	213	213	191	209	198	198	165	170
Aa3930	9	431	431			201	203	182	182	412	412	253	256	238	238	230	235	213	213	191	195	198	201	175	175
Aa3931	9	427	431	137	137	195	217	184	202	412	412	250	256	236	240	230	230	213	213	197	207	195	195	170	175
Aa3932	9	427	431	125	125	217	221	194	206	412	412	250	256	242	264	230	230	201	201	197	209	198	198	175	175
Aa3933	9	427	429	137	155	201	201	194	204	412	412	256	256	236	236	230	230	213	213	207	207	195	198	165	165
Aa3934	9	429	429			193	201	182	182	412	418	253	256	236	236	230	230	217	217	191	207	195	201	170	170
Aa3935	9	427	427	137	137	187	199	182	194	412	412	250	250	238	322	230	230	201	213	197	197	195	198	170	170
Aa3936	9	429	429	125	137	201	203	182	192	412	412	253	256	230	230	230	230	201	205	207	209	195	201	190	190
Aa3939	10	431	431	125	137	191	201	184	192	412	412	250	250	236	236	230	230	205	213	197	209	195	195	170	170
Aa3940	10	427	431	133	135	205	205	194	194	412	412	250	250	236	286	230	230	201	213	191	191	195	198	170	170
Aa3941	10	431	431	125	125	217	221	182	182	412	418	256	256	236	264	230	230	205	213	191	191	195	198	165	170
Aa3942	10	429	431	141	141	221	221	194	194	412	418	250	256	236	236	230	230	201	209	207	207	195	198	170	170
Aa3943	10	431	431	125	125	201	201	182	186	412	412	256	256	236	240	230	230	205	213	199	207	195	198	165	165
Aa3944	10	431	431	135	137	205	213	182	206	412	412			236	236	230	230	201	209	191	207	195	195	175	175
Aa3945	10	431	431	155	167	217	221	186	186	412	412	250	253	236	248	230	230	213	213	191	207	195	195	175	175
Aa3946	10	427	429	125	125	201	211	194	194	412	412			236	258	230	235	213	213	191	197	195	198	170	170
Aa3947	10	425	431	135	135	189	217	184	184	412	418	256	256	230	260	230	230	213	217	191	207	198	198	170	175
Aa3948	10	427	431	133	139	191	213	182	186	412	412	256	256	250	250	230	230	209	209	207	207	195	198	175	175
Aa3949	10	425	433	137	139	199	209	182	182	412	412	256	256	228	228	230	230	213	213	205	207	198	201	170	170
Aa3950	10	427	431	137	137	205	221	182	182	412	412	253	256	240	258	230	230	201	213	207	207	195	201	170	170
Aa3951	10	431	431	125	137	217	233	182	182	412	412	253	256	228	230	230	230	205	213	191	207	195	198	170	170
Aa3952	10	427	431	125	137	199	217	182	182	412	412			238	238	225	225	201	205	207	207	195	198	170	175
Aa3953	10	427	429	143	143	201	231	182	182	412	412	253	253	236	236	230	230	201	213	195	195	195	195	190	190
Aa3954	10	427	427	125	137	213	231	206	208	412	412	250	256	236	236			205	213	191	195	198	201	170	190
Aa3955	10	429	431	137	137	213	219	184	192	412	412	250	253	236	236	230	230	205	205	191	191	195	195	170	170
Aa3956	10	427	429	137	137	217	221	182	184	412	412	250	250	238	238	235	235	201	213	191	195	192	201	170	170
Aa3957	10	429	429	125	151	211	219	194	202	412	412	262	262	236	264	230	235	209	209	207	207	198	201	170	170
Aa3958	10	429	431	137	137	203	205	182	194	412	418	250	256	260	264	230	230	213	213	197	197	198	201	175	175
Aa3959	10	427	427	133	137	201	213	194	194	412	412	250	256	266	322	230	230	213	213	191	207	195	195	165	175
Aa3960	10	427	431	143	143	195	221	182	182	412	412	250	250	236	236	230	230	209	213	191	191	195	195	175	190
Aa3961	10	427	427	137	137	215	215	182	182	412	412	253	256	236	236	230	230	201	213	191	207	195	195	170	170
Aa3962	10	427	431	139	139	213	221	182	194	412	412	256	256	268	268	230	230	201	213	207	207	195	201	165	165
Aa3963	10	427	429	125	125	201	213	182	182	412	412	250	256	322	322	230	230	213	213	189	207	195	201	175	175
Aa3964	10	429	429	137	137	217	219	182	206	418	418	250	250	236	236	230	230	201	213	191	195	195	198	170	175
Aa3965	10	427	431	125	137	205	213	192	192	412	418	250	250	236	236	230	230	209	213	207	209	201	201	175	175
Aa3966	10	425	425	135	135	199	201	182	182	412	412	256	256	240	320	230	230	205	205	191	197	195	201	170	170
Aa3967	10	429	429	137	137	217	231	192	194	412	412	250	250	238	258	230	230	209	217	207	207	195	198	190	190
Aa3968	10	427	431			203	221	194	194	412	412	256	256	228	228	230	230	205	209	195	195	195	198	175	190
Aa3969	10	431	431			201	213			412	418	253	253	238	264	230	230	213	213	207	207	195	198	175	175
Aa3970	10	427	429	137	139	205	231	182	182	412	412	250	256	228	236	230	230	209	213	191	207	195	195	170	170
Aa3971	10	429	429	125	139	201	205	182	182	412	412	256	256	232	236	230	230	205	213	191	207	195	198	180	180
Aa3972	10	431	431	137	137	217	217	182	194	412	418	253	259	236	264	230	230	201	213	191	207	195	198	170	170
Aa3973	10	431	431	137	137	217	217	182	182	412	412	253	256	264	264	230	230	209	209	197	209	195	198	170	175
Aa3974	10	429	429	125	135	213	221	182	206	412	412	256	256	264	264	230	230	205	209	191	209	195	198	165	165
Aa3975	10	429	431	145	165	213	217	192	206	412	412	250	256	260	264	230	230	193	213	195	207	195	195	170	175
Aa3976	10	431	431	125	137	203	219	192	192	412	412	256	256	236	280	230	230	213	213	193	209	192	195		
Aa3977	10	431	431	137	137	211	213	182	194	412	412	253	253	236	240	230	230	213	213	207	207	198	198	165	175
Aa3978	10	427	427	125	143	201	213	182	184	412	412	253	256	260	266	230	230	213	213	191	207	195	195	170	170
Aa3979	10	427	427	137	137	201	217	182	182	412	412	253	256	236	270	230	230	213	213	191	191	201	201	170	175
Aa3980	10	431	431	137	137	201	211	186	198	412	412	253	253	230	240	230	230	205	209	191	207	198	198	170	170
Aa3981	10	427	431	133	133	201	211	206	206	412	412	250	250	238	258	230	230	213	213	191	207	198	198	175	185
Aa3982	10	427	429	137	137	213	231	184	192	412	412	250	253	236	236	230	230	213	217	191	191	195	198	165	165
Aa3983	10	429	431	141	141	201	225	206	206	412	412	250	250	238	238	230	230	209	209	191	191	195	198	170	170
Aa3984	10	427	429			201	217	182	206	412	412	250	256	238	238	230	235	201	205	207	207	195	195	175	190
Aa3985	10	431	431	141	141	201	203	184	204	412	412	250	250	230	230	230	230	213	217	195	207	195	198	170	170
Aa3986	10	431	431	125	139	213	223			412	412	250	250	240	240	230	230	209	213	191	191	195	195	165	165

Aa3987	10	427	431	137	137	201	223	194	194	412	418	256	256	230	322	225	225	213	213	207	207	198	198	170	175
Aa3988	10	425	431	137	141	217	231	182	182	412	412	250	256	236	264	230	230	209	213	195	195	195	195	170	170
Aa4653	10	431	431			217	213	192	194	412	412	250	256	238	242	230	230	201	213	191	195	195	201	190	190
Aa4654	10	427	431	139	139	213	231	192	194	412	412	256	256	240	260	230	230	209	213	191	207	195	195	170	175
Aa4655	10	431	431	137	137	201	227	182	194	412	412	250	256	230	230	230	230	209	213	191	209	198	198	170	170
Aa4656	10	427	431	133	143	201	207	182	194	412	412	253	256	236	236	230	235	209	213	191	207	192	198	170	170
Aa4657	10	431	431	133	133	225	225	182	196	412	418	253	256	236	236	230	230	213	217	195	195	195	195	165	170
Aa4658	10	429	431	139	141	211	215	194	194	418	418	241	250			230	230	205	213	197	197	195	195	165	170
Aa4659	10	431	431			211	217	186	186	412	418	244	256	236	236	230	235	209	213	191	207	195	195	170	170
Aa4660	10	427	431	125	141	195	213	194	198	412	418	250	256	260	264	225	225	193	213	195	195	195	195	175	190
Aa4661	10	431	431	137	137	201	201	194	194	412	412	256	256	236	280			205	209	191	191	195	195	170	185
Aa4662	10	431	431	155	155	201	201	182	196	412	412	256	256	240	258	235	235	205	213	207	207	198	198	165	170
Aa4663	10	431	435	143	143	213	223	182	192	412	412	250	250	230	270	230	230	209	209	207	207	198	204	165	170
Aa4664	10	429	431	155	155	205	205	186	186	418	418	250	250	238	238	230	230	205	209	191	207	195	195	180	180
Aa4665	10	431	431	137	155	205	207	182	182	412	412	256	256	264	264	230	230	209	213	191	191	195	195	165	190
Aa4666	10	431	431	165	165	217	223			412	412	253	256	236	236	230	230	213	213	207	207	195	195	175	175
Aa4667	10	427	427	169	169	201	231	182	182	412	412	256	271	240	260	235	235	205	213	195	195	195	198	170	170
Aa4668	10	427	429	131	131	231	231	182	196	412	412	250	250	258	258	230	230	209	209	195	195	195	198	170	190
Aa4669	10	427	431	125	125	201	211	192	206	412	412	253	253	264	322			213	213	191	191	195	195	190	190
Aa4670	10	427	431	125	125	207	217	182	182	418	418	253	256	236	260	230	230	213	213	191	207	195	198	170	175
Aa4671	10	427	431	125	137	195	219	188	206	412	412	250	253	236	236	230	230	213	213	205	205	195	198	170	170
Aa4672	10	429	431	125	125	191	217	182	182	412	412	256	256	236	236	230	230	213	213	207	207	195	198	170	170
Aa4673	10	429	431	125	125	205	217	182	192	412	412	244	250	238	238	230	230	205	209	191	195	195	195	170	170
Aa4674	10	427	431			201	219	182	194	412	412	256	256	240	322	230	230	209	209	207	207	195	198	170	175
Aa4675	10	431	431	135	135	201	231	194	194	412	412	244	253	238	238	230	230	213	213	191	207	195	195	170	170
Aa4676	10	427	431	137	151	201	217	182	182	412	412	256	256	240	240	225	230	213	213	191	195	195	198	170	170
Aa4677	10	425	431	125	125	205	213	182	194	412	412	250	271	236	264	230	230	201	201	191	191	195	195	170	170
Aa4678	10	431	431	135	137	217	217			412	412	250	256	238	266	230	235	205	209	207	207	195	198	170	190
Aa4679	10	427	427	137	139	213	231	180	182	412	412	241	250	236	236	230	230	213	213	191	207	198	198	170	170
Aa4680	10	427	431	137	137	217	217	194	194	412	412	250	250	248	264	230	230	201	209	195	207	195	195	190	190
Aa4681	10	431	431	137	139	201	217	182	202	412	412	256	256	230	236			205	213	191	191	195	195	170	170
Aa4682	10	427	431	133	137	201	201	184	184	412	412	250	256	236	238	230	230	201	201	191	207	195	195	165	190
Aa4683	10	429	431	155	155	217	217	182	182	412	412	250	256	230	238	230	230	213	213	207	207	195	198	170	170
Aa4684	10	429	431	137	139	221	223	194	194	412	412	250	256	230	264	230	230	205	209	191	195	195	198	165	170
Aa4685	10	431	431	125	125	201	227	182	192	412	412	256	256	240	282	230	230	213	213	197	207	198	198	170	170
Aa4686	10	431	431	137	137	199	199	184	184	412	412	253	253	248	248	230	235	213	213	191	207	195	198	175	190
Aa4687	10	429	431	125	133	187	199			412	412	250	256	238	264	230	230	213	213	207	207	195	198	165	175
Aa4688	10	427	427			201	219	182	194	412	418	244	253	230	238	230	230	209	213	191	197	195	198	165	165
Aa4689	10	431	431	155	155	201	213	170	182	412	418	250	256	266	280	230	230	205	213	191	207	195	198	170	170
Aa4690	10	427	427	125	125	217	217	182	182	412	412	253	256	236	238	230	230	201	209	191	191	195	198	175	175
Aa4691	10	431	431	137	137	201	201	182	186	412	412	250	256	236	322	230	230	213	213	191	191	201	201	170	170
Aa4692	10	429	431	137	137	213	221	184	194	412	412	250	253	238	238	235	235	209	209	191	207	195	198	170	170
Aa4693	10	429	431	139	139	205	221	194	194	412	412	241	250	228	228	230	230	205	213	207	207	195	195	165	165
Aa4694	10	429	429	151	165	207	213	182	202	412	412	253	256	236	264	235	235	213	213	207	207	195	195	190	190
Aa4695	10	427	429	125	137	219	221	186	194	412	412	250	256	240	240	230	230	213	217	191	207	195	195	185	190
Aa4696	10	427	427	137	137	213	221	182	194	412	412	253	253	236	236	230	230	205	213	205	205	198	198	170	170
Aa4697	10	429	431	137	137	213	217	182	194	412	412	256	256	236	236	235	235	213	213	207	207	195	195	175	175
Aa4698	10	427	429	151	151	201	201	182	198	412	412	253	253	260	260	230	230	213	213	191	207	195	198	165	170
Aa4699	10	427	427	137	137	201	217	182	184	412	412	250	259	240	258	230	230	193	213	191	195	195	195	175	175
Aa4700	10	427	429	139	139	213	221	182	184	412	412	244	250	238	238	230	235	205	213	191	207	195	198	165	165
Aa4701	10	431	431	125	139	207	207	182	182	412	412	250	256	236	236	230	230	201	201	191	191	195	198	170	180
Aa4702	10	431	431	125	125	203	223	182	184	412	418	250	250	250	250	230	230	193	213	205	207	195	195	170	170
Aa4041	11	429	429	137	137	213	223	182	194	412	412	250	256	236	322	230	230	213	213	197	197	195	198	170	170
Aa4042	11	427	431	137	139	201	227	182	192	412	412	259	259	236	248	230	230	201	205	207	209	198	201	170	170
Aa4043	11	427	427	125	137	205	219	182	182	412	412	250	253	230	264	230	230	201	209	207	207	201	201	165	190
Aa4044	11	431	431	137	137	187	221	182	182	412	412	241	253	236	264	230	230	213	213	191	209	201	201	180	185
Aa4045	11	427	429	125	135	201	207	182	184	412	418	256	256	238	238	230	230	201	213	191	207	195	201	165	165
Aa4046	11	431	431	125	125	221	221	182	182	412	412	256	256	236	260	230	230	205	213	191	191	198	201	175	175
Aa4047	11	431	431	125	125	205	217	204	204	412	412	250	250	236	264	230	230	209	213	189	195	195	198	170	175
Aa4048	11	431	431	123	137	205	231	182	182	412	412	241	250	236	236	230	230	201	201	191	207	198	198	175	190
Aa4049	11	431	431			199	213	192	194	412	412	250	250	236	236	230	230	205	213	191	207	198	201	170	175
Aa4050	11	431	431	125	125	205	211	182	194	412	412			240	240	230	230	213	213	195	197	195	198	165	175
Aa4051	11	431	431	125	137	187	215	192	194	412	412	250	256	238	264	235	235	201	209	191	209	195	201	170	170
Aa4052	11	427	435	137	137	205	225	182	182	412	412	250	253	260	260	230	230	209	209	191	191	195	195	170	170
Aa4053	11	429	429	127	169	199	221	184	184	412	412	250	250	236	236	230	230	205	205	191	191	195	195	175	175
Aa4054	11	429	431	125	125	195	201	182	182	412	418	250	250	236	240	230	235	209	213	191	207	195	201	170	170
Aa4055	11	427	431	125	157	191	201	180	186	412	412	250	256	236	240	230	230	193	209	191	191	195	195	170	170
Aa4056	11	431	431	139	139	197	197	180	194	412	412	256	256	236	236	230	230	213	213	191	191	195	201	165	170
Aa4057	11	431	431	133	137	201	225			412	412	250	250	236	240	235	235	205	213	191	207	195	198	175	175
Aa4058	11	429	429	133	143	217	219	196	196	412	412	250	256	236	236	235	235	213	213	191	191	195	198	170	170
Aa4059	11	427	431	135	137	199	205	184	194	412	412	256	256	236	236	230	235	213	213	207	207	195	198	165	170
Aa4060	11	431	431	139	157	187	203	182	204	412	418	241	256	240	280	230	230	213	213	207	207	195	201	165	175
Aa4061	11	431	431	135	135	203	217			412	412	250	253	236	264	225	230	205	213	191	207	195	195	170	175
Aa4062	11	429	431	125	125	201	231	180	180	412	412	250	256	314	314	230	235	209	209	191	207	201	201	170	180
Aa4063	11	431	431	137	137	203	213	198	198	412	412	250	256	238	238	230	230	205	213	191	207	195	198	170	170
Aa4064	11	431	431	125	125	203	231	192	202	412	412	253	253	248	248	225	230	205	213	207	207	192	198	170	170
Aa4065	11	431	431	137	137	199	203	186	206	412	412	250	256	236	260	230	230	209	213	191	191	198	201	170	175
Aa4066	11	427	429	137	139	205	231	182	202	412	412	256	256	230	236	230	230	205	209	191	197	198	198	190	190

Aa4067	11	427	431	133	133	221	231	182	192	412	418	244	250	264	264	230	230	209	209	191	191	195	198	165	175
Aa4068	11	427	429	125	137	205	217	182	192	412	418	250	256	236	236	230	230	205	213	191	191	195	198	190	190
Aa4069	11	431	431	125	125	187	207	182	184	412	412	253	253	260	264	230	230	209	213	207	207	195	201	190	190
Aa4070	11	427	431	125	167	217	227	182	194	412	418	256	256	236	236	230	230	201	213	191	191	195	198	170	175
Aa4071	11	429	431			201	217	184	194	412	412	250	253	236	260	230	230	193	213	191	195	195	195	170	175
Aa4072	11	427	431	137	139	191	201	194	194	412	418	244	250	236	236	230	230	209	209	191	191	195	198	170	170
Aa4073	11	427	431	139	157	205	223	184	192	412	412	250	256	238	238	230	230	205	213	207	207	195	195	165	170
Aa4074	11	429	431	137	137	213	213	194	194	412	418	250	256	236	240	230	230	201	213	191	207	195	201	170	170
Aa4075	11	431	431	125	125	217	221	182	182	412	412	256	256	236	236	230	230	213	213	207	209	195	201	170	170
Aa4076	11	427	427			217	217	184	206	412	412	250	250	236	236	230	230	213	213	207	207	195	198	190	190
Aa4077	11	427	431	139	139	203	215	184	192	412	412	250	256	298	298	230	230	209	209	207	209	195	195	170	175
Aa4078	11	431	431	139	157	217	219	184	194	412	412	253	271	238	238	230	230	213	213	191	191	195	201	170	170
Aa4079	11	429	429	125	137	201	227	186	186	412	412	244	244	236	240	230	235	193	193	191	191	195	198	175	190
Aa4080	11	431	431	143	143	205	207	182	182	412	412	256	256	236	236	230	230	209	213	191	207	195	198	190	190
Aa4081	11	431	431	137	137	213	213	182	206	412	412	253	262	248	264	230	230	205	213	195	195	195	201	165	170
Aa4082	11	431	431	139	139	219	231	198	198	412	412	256	256	236	320	230	230	213	213	191	207	195	195	175	175
Aa4083	11	431	431	137	137	213	215	182	194	412	412	253	256	236	280	230	230	213	213	207	209	195	195	170	170
Aa4084	11	431	431	125	135	213	213	184	206	412	412	250	256	230	240	230	230	213	217	195	195	195	198	170	175
Aa4085	11	429	431	137	137	201	201	182	182	412	412	250	250	236	240	230	230	213	213	191	207	195	195	170	170
Aa4086	11	431	433	165	165	221	221	182	198	412	412	250	250	238	320	230	230	213	213	191	191	195	195	170	175
Aa4087	11	429	429	137	137	201	221	182	182	412	412	250	256	228	260			209	213	191	207	198	201	165	170
Aa4088	11	431	431	169	169	201	211	182	206	412	412	250	250	236	236	230	230	213	213	207	207	195	195	175	190
Aa4089	11	427	429	139	139	211	231	182	204	412	412	250	253	236	236	230	230	213	213	207	207	198	201	165	165
Aa4090	11	431	431	139	143	213	231	182	208	412	418	256	256	264	264	235	235	205	213	191	207	195	198	175	190
Aa4091	11	427	429	145	167	201	201	182	184	412	412	253	256	236	314	230	230	193	213	191	195	198	198	170	170
Aa4092	11	427	431			213	213	192	194	412	412	253	256	258	264			193	213	197	207	195	198	170	170
Aa4093	11	425	427			213	231	192	206	412	412	250	250	236	236	230	230	213	213	207	207	195	198	165	175
Aa4094	11	431	431			199	213	182	182	412	412	253	256	264	264			205	213	207	207	198	198	170	170
Aa4095	11	429	431	125	137	237	237	182	184	412	412	253	256	236	322	230	230	209	213	207	207	195	195	175	190
Aa4096	11	429	429	137	137	201	205	182	194	412	412	250	256	230	236	230	230	209	213	191	207	198	198	165	170
Aa4097	11	431	431	143	143	201	231	182	188	412	412	250	250	236	240			201	205	191	195	195	198	165	165
Aa4098	11	429	431	137	167	213	231	182	206	412	412	253	253	236	264	230	230	213	213	195	195	195	198	190	190
Aa4099	11	429	431	145	167	203	203	184	192	412	412	250	262	240	264	230	230	213	213	191	207	195	198	165	170
Aa4100	11	429	431	125	125	191	221	170	194	412	412	253	256	260	264	230	230	209	213	171	191	198	198	170	170
Aa4101	11	431	431	125	125	201	213	194	194	412	412	256	256	236	264			205	213	197	209	195	195	170	180
Aa4102	11	429	429	125	125	187	217	182	182	412	412	250	253	260	264	230	230	205	213	207	207	198	201	170	170
Aa4103	11	431	431	125	125	217	217	194	194	412	412	250	256	258	258	230	230	205	209	207	207	195	195	170	170
Aa4104	11	431	431	125	125	217	231	184	184	412	412	253	262	236	264	230	235	209	213	197	197	195	201	170	175
Aa4105	11	427	427	125	125	213	231	180	182	412	412	250	250	236	270	230	230	205	213	195	197	195	198	175	190
Aa4106	11	427	431	135	135	203	231	182	182	412	412	250	250	236	240			213	213	207	207	195	198	175	175
Aa4107	11	429	431	125	137	195	221	184	194	412	412	256	256	238	314	230	230	209	209	191	197	195	198	165	165
Aa4108	11	431	431	125	137	207	217	184	186	412	412	250	256	224	224	230	235	213	213	191	207	195	198	170	170
Aa4109	11	427	427			223	231	182	182	412	412	250	259	260	260	230	230	205	205	207	207	198	204	170	170
Aa4110	11	427	429	125	125	217	217	182	182	412	412			236	236	230	230	213	213	205	205	195	198	170	170
Aa4111	11	427	429	125	125	217	217	184	188	412	418	253	253	236	236	230	230	213	213	195	207	198	198	165	190
Aa4112	11	427	429	125	125	205	213	182	186	412	412	250	253	240	240	235	235	205	213	195	197	195	201	175	175
Aa4113	11	431	431	125	125	205	207	192	192	412	412	253	253	236	314	230	230	201	213	191	207	195	201	175	175
Aa4114	11	427	431	125	125	201	217	182	182	412	412	256	256	236	260	230	230	213	213	197	197	195	198	165	170
Aa4115	11	429	431	125	125	225	227	182	192	412	412	250	253	264	264	230	230	205	209	207	209	195	195	165	165
Aa4116	11	427	427	125	125	217	219	182	206	412	412	247	247	228	236	230	230	201	213	207	207	195	201	170	170
Aa4117	11	427	431	125	125	205	213	186	194	412	412	253	253	236	264	230	230	209	213	195	209	195	198	165	175
Aa4118	11	427	431	125	125	205	227	188	188	412	412	256	256	236	260	230	230	205	213	195	195	195	201	165	170
Aa4119	11	427	427	125	125	205	215	198	198	412	412	250	256	230	248	230	230	201	205	191	207	195	198	165	190
Aa4120	11	427	427	125	125	187	201	194	206	412	412	250	256	236	238	230	230	205	205	191	191	195	198	180	180
Aa4121	11	431	431	125	125	205	205	192	194	412	412	253	253	236	264	230	230	213	213	189	189	195	201	170	170
Aa4122	11	431	431	125	125	197	201	184	194	412	412	250	256	236	236	230	235	193	213	207	207	198	198	165	170
Aa4123	11	427	429	125	125	211	231	182	206	412	412	256	256	228	260	230	230	213	213	195	207	195	195	170	170
Aa4124	11	431	431			201	213			412	412	250	250	248	248	230	230	205	205	191	207	195	195	165	190
Aa4125	11	429	431	125	125	211	231	182	194	412	412	253	253	236	236	230	230	205	213	207	207	195	204	170	170
Aa4126	11	429	429	125	125	231	231	192	202	412	412	250	253	236	264	230	230	205	205	207	207	195	195	170	170
Aa4127	11	431	431	125	125	221	231	194	206	412	418	250	250	236	264			205	205	191	207	195	201	180	180
Aa4128	11	431	431	125	125	213	231	194	194	412	412	250	250	228	242	230	230	213	213	195	207	195	198	165	165
Aa4129	11	431	431	125	125	187	231	186	206	412	412	253	253	236	258	230	230	209	213	191	191	195	195	175	175
Aa4130	11	431	431	125	145	205	221	192	194	412	412	250	250	240	240	230	230	209	213	207	209	195	195	170	170
Aa4131	11	431	431	137	139	197	213	194	194	412	412	250	250	244	264	230	230	205	213	191	195	195	198	170	170
Aa4132	11	431	431	137	137	201	207	182	192	412	412	253	253	240	280	230	230	205	209	191	191	195	195	170	175
Aa4133	11	431	431	137	137	201	205	182	196	412	412	244	250	236	236	230	230	213	213	197	197	195	195	165	170
Aa4134	11	427	427	137	137	199	201	182	184	412	418	256	256	238	264	230	235	205	213	191	207	195	198	170	170
Aa4135	11	427	431	125	157	187	213	182	204	412	412	253	256	230	236	230	230	213	213	207	207	195	195	170	170
Aa4136	11	431	431	125	145	221	221	182	206	412	412	250	256	270	270	235	235	213	213	191	207	198	198	170	170
Aa4137	11	427	431	125	125	213	213	182	202	412	412	250	256	236	238	230	235	213	213	195	207	198	201	170	170
Aa4138	11	429	431	137	137	203	205	182	206	412	418	253	253	236	258	230	230	205	209	191	191	195	195	170	175
Aa4139	11	431	431	157	157	201	201	182	188	412	412	250	256	224	236	230	230	213	213	207	209	198	204	185	185
Aa4140	11	429	431	133	137	213	221	182	182	412	412	253	256	236	260	230	235	213	213	191	195	195	201	175	175
Aa4143	12	429	429	137	137	201	219	182	182	412	412	253	256	240	314	230	230	201	213	191	207	195	198	170	175
Aa4144	12	431	431	137	143	221	221	192	194	412	412	253	253	236	270			201	209	191	191	198	201	170	170
Aa4145	12	425	429	155	155	213	217	182	206	412	418	256	256	236	264	230	235	201	217	207	207	195	198	165	170
Aa4146	12	427	429	125	139	205	221	184	198	412	412	250	256	238	238	230	230	213	213	207	207	195	198	185	185

Aa4147	12	427	431	137	137	199	199	192	192	412	412	250	256	236	248	230	230	209	213	191	207	195	195	170	170
Aa4148	12	425	431	137	137	205	213	182	204	412	418	250	250	236	264	235	235	213	213	207	207	195	201	170	175
Aa4149	12	427	431	137	143	205	221	188	198	412	418	250	271	264	264	230	230	213	213	191	207	195	195	170	175
Aa4150	12	427	431			201	213	182	182	412	418	241	250	236	240	230	230	205	213	207	209	198	201	170	170
Aa4151	12	427	431	125	137	201	201	182	182	412	412	256	256	240	248	230	230	209	209	193	193	195	195	170	175
Aa4152	12	427	427	137	137	199	213	206	206	412	418	250	256	244	264	230	230	209	213	191	207	195	195	170	170
Aa4153	12	427	429	125	125	187	201			412	412	250	256	238	320	230	235	201	213	207	207	195	198	170	190
Aa4154	12	427	431	139	139	213	215	182	194	412	412	250	256	232	238	230	230	201	205	207	207	195	201	165	175
Aa4155	12	429	429	141	141	217	217	182	192	412	412	250	256	238	238	230	230	209	209	207	207	195	198	170	170
Aa4156	12	429	431	137	165	217	231	204	204	412	418	250	256	236	240	230	230	213	213	207	207	195	195	175	175
Aa4157	12	427	429	125	137	199	213	182	182	412	412	256	256	224	238	230	230	209	209	207	207	198	201	175	175
Aa4158	12	427	431	137	137	207	213	182	182	412	412	241	256	236	236	235	235	205	213	197	197	195	198	170	170
Aa4159	12	431	431	139	139	191	231	182	182	412	412			232	236	230	230	201	213	191	209	198	198	175	175
Aa4160	12	431	431	137	165	201	213	182	208	412	412	250	253	236	314	230	230	193	213	205	205	198	198	165	165
Aa4161	12	431	431	137	139	201	231	182	182	412	412	250	256	258	258	230	230	213	213	189	209	195	201	175	175
Aa4162	12	427	429	137	139	201	205	182	206	412	412	250	253	232	270	230	230	213	213	197	207	195	198	170	180
Aa4163	12	431	431	139	139	203	211	182	186	412	412			238	238	230	230	213	213	207	207	195	198	170	175
Aa4164	12	431	431	137	137	201	221	182	194	412	412	250	256	228	238	230	235	213	213	207	209	195	195	170	185
Aa4165	12	427	431	137	137	203	231	194	194	412	412	256	256	236	236	230	230	209	209	191	191	195	198	175	175
Aa4166	12	429	431			211	223	184	184	412	412	253	256	264	264	230	230	213	213	189	195	198	201	170	170
Aa4167	12	427	429	133	137	199	217	194	194	412	412	250	253	236	270	230	230	201	213	191	207	198	201	170	170
Aa4168	12	431	431	137	137	205	219			412	412	253	256	236	236	230	230	213	213	191	191	195	198	170	170
Aa4169	12	431	431			213	217	182	194	412	412	256	256	314	314	230	230	213	213	191	207	198	198	170	170
Aa4170	12	427	431	137	137	187	213	194	194	412	412	250	256	236	236	230	235	193	193	197	197	195	201	170	170
Aa4171	12	427	429			201	221	184	192	412	418	250	256	236	236	230	230	201	213	207	207	195	198	170	170
Aa4172	12	427	429	137	141	215	217	184	192	412	412	250	256	270	280	230	230	205	217	191	191	195	195	175	175
Aa4173	12	431	431	137	137	199	201	182	192	412	412	241	256	258	264	230	230	213	213	195	207	195	198	170	170
Aa4174	12	431	431	137	137	203	207	182	182	412	418	250	256	236	240	230	230	209	213	191	195	195	201	175	190
Aa4175	12	427	431	125	139	221	223	182	208	412	418	250	262	236	264	230	235	205	213	197	197	195	198	170	175
Aa4176	12	429	431	169	169	231	231	182	186	412	412	250	250	240	260	230	230	205	205	191	207	195	198	175	175
Aa4177	12	431	431	125	125	195	231	182	182	412	412	250	256	264	264	230	230	205	205	195	207	195	195	175	190
Aa4178	12	427	427	139	165	203	215	182	194	412	412	256	256	236	322	230	230	193	213	195	195	195	195	170	170
Aa4179	12	431	431	133	133	213	217	194	194	412	412	250	256	238	238	230	230	213	213	207	207	195	195	170	175
Aa4180	12	429	431	125	125	213	221	194	198	412	418	250	250	238	248	230	230	213	213	193	197	195	195	170	170
Aa4181	12	427	431	125	125	195	217	182	194	412	412	256	262	238	264	230	230	201	205	207	207	195	198	170	175
Aa4182	12	429	431	137	137	201	205	184	192	412	418	256	256	236	236	230	230	209	217	207	207	198	198	170	170
Aa4183	12	429	431	137	137	215	217	182	182	412	412	256	256	236	270	230	230	209	213	191	207	195	198	170	170
Aa4184	12	429	429	125	125	201	205	186	192	412	418	256	256	240	264	230	230	213	213	195	195	195	198	170	190
Aa4185	12	427	427	137	137	201	217	194	206	412	412	250	271	236	276	230	230	201	213	191	191	195	198	170	170
Aa4186	12	431	431	137	137	205	231	182	192	412	412	253	253	236	236	230	230	201	213	195	207	195	201	170	170
Aa4187	12	427	431	137	137	205	213	192	206	412	412	250	256	236	236	230	230	213	213	195	195	195	201	190	190
Aa4188	12	431	431	139	159	205	229	182	184	412	412	250	262	264	322	230	230	201	213	191	191	195	195	175	175
Aa4189	12	431	431	137	137	207	213	184	194	412	412	256	256	240	286	230	230	201	213	191	207	195	195	165	165
Aa4190	12	429	431			199	201	182	184	412	412	250	256	260	260			201	213	197	209	195	195	170	170
Aa4191	12	431	431	125	137	213	213	182	192	412	412	250	250	238	238	230	230	205	213	197	197	195	195	165	175
Aa4192	12	427	431			217	223	182	184	412	412	250	256	248	258	230	230	213	213	189	207	195	198	190	190
Aa4193	12	427	431			201	213	184	184	412	412	253	256	232	316	230	230	209	213	191	207	195	201	170	180
Aa4194	12	429	431			205	205	182	192	412	412	250	250	258	274	230	230	209	213	191	195	195	198	175	190
Aa4195	12	427	429			203	217	206	206	412	412	250	262	264	274	230	230	213	213	207	209	195	198	190	190
Aa4196	12	427	431			199	213	182	184	412	412	250	250	230	238	230	230	213	213	191	207	195	198	170	170
Aa4197	12	427	429	125	125	201	205	184	194	412	412	247	250	236	266	230	230	201	209	191	207	195	198	165	170
Aa4198	12	425	431	125	125	211	213	182	194	412	412	253	256	230	236	230	230	205	213	191	207	198	201	170	175
Aa4199	12	429	431	125	125	201	217	182	194	412	412	250	250	236	236	230	230	193	213	191	191	198	201	170	170
Aa4200	12	431	431	125	137	201	221	182	182	412	412	256	256	260	322	230	230	213	213	207	207	195	198	170	170
Aa4201	12	431	431			201	209	182	182	412	412	256	271	238	238	230	230	201	205	191	197	195	201	170	180
Aa4202	12	431	431			227	229	182	194	412	412	250	250	248	260	230	230	213	213	207	207	195	195	170	175
Aa4203	12	429	429			187	201	184	194	412	412	256	256	236	238	230	230	201	209	191	207	195	198	165	165
Aa4204	12	427	427	125	137	203	213	194	194	412	412	253	256	236	236	230	230	209	213	207	207	195	201	190	190
Aa4205	12	431	431	133	137	191	195	184	204	412	412	250	250	232	266	230	230	193	201	191	195	195	198	165	165
Aa4206	12	431	431	137	149	201	205	186	186	412	412	256	256	236	260	230	230	205	213	191	207	198	198	170	170
Aa4207	12	431	431	137	165	201	231	182	182	412	412	253	256	236	248	230	230	209	213	207	209	195	195	170	170
Aa4208	12	427	429	125	125	201	223	194	194	412	412	241	253	230	238			209	213	207	207	198	198	170	170
Aa4209	12	427	431	137	143	201	223	192	206	412	412	256	256	230	230	230	230	201	205	195	207	195	201	175	190
Aa4210	12	431	431	137	137	211	217	182	194	412	412	250	256	238	238	230	230	213	213	191	207	195	204	170	170
Aa4211	12	427	431			205	213	184	194	412	412	250	256	238	238	230	230	213	213	207	207	198	198	170	175
Aa4212	12	431	431	135	137	213	217	182	182	412	412	250	250	228	248	230	230	205	213	195	197	195	201	175	190
Aa4213	12	431	431	125	125	191	205	182	182	412	418	250	256	236	236	230	230	213	213	197	209	195	198	175	190
Aa4214	12	427	429	135	167	187	201	182	206	412	412			236	248	230	235	213	213	207	207	195	198	165	175
Aa4215	12	427	431	125	133	205	213	182	182	412	412	253	253	264	264	230	235	213	213	191	191	195	195	170	185
Aa4216	12	431	431	137	139	201	213	182	182	412	412	253	256	236	238	230	230	205	213	191	195	198	198	170	170
Aa4217	12	431	431	135	165	205	213	182	182	412	412	253	253	236	236	230	230	213	213	207	207	195	198	175	175
Aa4218	12	431	431	137	137	205	205	194	202	412	418	250	250	226	226	230	230	213	213	207	207	195	198	175	190
Aa4219	12	431	431	135	137	213	213	194	194	412	412	253	253	264	264	230	230	209	213	197	197	195	195	170	175
Aa4220	12	431	431	137	137	213	217	192	192	412	412	250	256	248	248			209	213	207	207	195	198	165	170
Aa4221	12	427	431	139	139	191	201	194	194	412	412	250	256	236	236	235	235	213	213						
Aa4222	12	429	431	137	151	195	199	182	182	412	412	253	256	236	248	230	230	209	213	207	207	195	198	165	165
Aa4223	12	429	431	137	151	195	199	182	182	412	412	253	256	236	248	230	230	209	213	207	207	195	198	165	165
Aa4224	12	427	431	135	139	193	217	182	202	412	412	256	256	236	322	230	230	213	213	191	191	195	195	170	170

Aa4225	12	427	427			211	217	182	182	412	412	250	256	236	240	230	230	201	213	191	209	195	198	175	180
Aa4226	12	429	429	125	137	187	231	182	186	412	412	253	256	236	238	230	230	213	213	207	209	195	198	170	170
Aa4227	12	431	431	125	125	195	227	182	194	412	412	253	253	236	236	230	230	213	213	191	191	195	195	170	170
Aa4228	12	427	429	125	139	211	217	192	194	412	418	250	256	230	236	230	230	205	209	191	197	195	198	170	170
Aa4229	12	429	431	125	125	205	213	206	206	412	412	256	256	236	264	235	235	213	213	191	191	198	198	170	190
Aa4230	12	427	431	139	151	201	205	192	194	412	412	253	256	236	238	235	235	201	213	191	197	195	198	170	170
Aa4231	12	431	431	137	157	187	211	182	206	412	412	250	250	236	236	230	230	213	213	191	209	195	195	170	175
Aa4232	12	431	431	151	165	211	217	182	208	412	418	253	256	236	264	230	230	209	209	207	207	195	201	170	170
Aa4233	12	431	431	137	137	187	203	188	194	412	418			260	264	230	230	213	213	191	191	195	198	170	170
Aa4234	12	431	431	135	139	213	213	182	182	412	412	253	253	240	264	230	235	201	205	195	195	195	198	165	170
Aa4235	12	429	431	137	137	201	205	182	192	412	412	250	253	236	260	230	230	201	213	191	207	195	195	170	170
Aa4236	12	431	431	125	125	199	199	182	182	412	412	250	256	236	236	230	230	205	205	207	207	195	201	175	190
Aa4237	12	431	431	139	139	205	213	206	206	412	412	241	241	236	236	230	230	205	205	207	209	195	198	175	175
Aa4238	12	429	431	137	137	197	231	182	206	412	412	253	256	238	260	230	235	209	213	191	191	195	198	175	190
Aa4239	12	431	431	137	137	215	217	182	182	412	412	250	253	230	236	225	230	213	213	191	207	195	195	170	170
Aa4240	12	427	427	125	137	201	217	182	206	412	412	253	253	238	264	230	230	205	209	197	207	198	198	170	175
Aa4241	12	427	429	137	137	201	205	182	194	412	412	250	256	236	236	230	230	213	213	191	207	198	201	170	175
Aa4242	12	431	431	125	125	201	217	182	182	412	412	250	250	240	270	230	230	213	213	191	207	198	198	170	170
Aa4245	13	431	431	139	139	213	231	182	184	412	412	253	256	264	264	230	230	213	213	207	207	195	195	165	170
Aa4246	13	429	431	137	155	217	217			412	418	241	256	230	268	230	230	209	213	207	207	195	195	170	170
Aa4247	13	431	431	157	157	211	221	182	182	412	412	253	256	236	258			209	213	191	191	195	198	170	170
Aa4248	13	431	431	137	139	205	205			412	412	253	253	264	264	230	230	205	209	191	191	195	201	170	170
Aa4249	13	429	429	139	139	213	231	194	194	412	412	253	253	236	236	230	235	209	213	191	195	195	198	175	175
Aa4250	13	431	431	133	133	213	215	184	184	412	412	253	253	236	238	235	235	213	213	195	207	195	198	170	170
Aa4251	13	431	431	143	143	217	219	182	208	412	412	250	250	236	236			201	213	191	205	195	198	170	175
Aa4252	13	427	427	135	137	211	221	182	182	412	412	253	253	236	236	235	235	213	213	207	207	195	201	190	190
Aa4253	13	431	431	137	137	201	221	182	194	412	412	256	256	230	248	230	230	201	209	197	209	195	195	170	175
Aa4254	13	429	429	137	137	217	217	182	194	412	412	253	253	230	236	230	235	205	209	207	207	201	201	190	190
Aa4255	13	427	431	165	165	201	213	184	184	412	412	244	253	260	260	230	235	201	201	197	197	195	201	170	170
Aa4256	13	431	431	137	139	223	231	186	194	412	412	250	250	228	228	230	230	201	213	195	195	198	198	170	170
Aa4257	13	431	431	125	125	195	213	192	194	412	418	253	256	236	238	230	230	213	213	207	207	195	195	175	175
Aa4258	13	429	433	137	137	231	235	182	182	412	412	250	250	236	258	230	230	205	209	191	191	195	198	170	175
Aa4259	13	429	429	137	155	199	205	186	192	412	412	250	250	236	270	230	230	201	201	191	197	195	198	170	175
Aa4260	13	427	431	125	137	197	215	182	186	412	412	250	256	236	264	230	230	213	217	207	207	198	201	170	170
Aa4261	13	431	431	125	137	201	211	182	194	412	412	256	256	236	320	230	230	213	213	207	207	195	195	170	170
Aa4262	13	429	431	137	139	207	231	182	182	412	412	253	256	236	240	230	230	213	213	207	207	198	198	175	175
Aa4263	13	429	429	125	125	201	221	194	206	412	412	253	256	260	260	230	230	205	213	197	197	192	195	170	175
Aa4264	13	427	431	137	137	199	207	182	194	412	412	256	256	270	270	235	235	209	213	207	207	198	198	170	170
Aa4265	13	431	431			205	213	194	204	412	412	256	256	236	236	230	230	201	213	207	207	195	198	170	175
Aa4266	13	431	431	137	137	201	213	198	198	412	418	253	256	236	236	230	230	213	213	207	207	195	195	190	190
Aa4267	13	431	431	133	167	213	235	182	182	412	412	250	253	236	236	230	230	205	213	207	207	195	201	170	190
Aa4268	13	429	431	125	125	197	237	182	182	412	412	256	256	236	236	230	230	209	209	207	207	195	201	175	175
Aa4269	13	427	427	137	153	187	221	182	184	412	412	253	256	236	238	230	230	205	213	207	207	195	201	170	170
Aa4270	13	427	433	169	169	223	231	182	206	412	412	256	256	266	280	230	230	213	213	207	207	198	201	170	175
Aa4271	13	431	431	125	139	213	231	182	192	412	412	250	250	238	240	230	230	205	213	207	207	195	198	170	170
Aa4272	13	431	431	137	143	215	217	182	206	412	412	256	256	238	238	230	230	205	213	197	207	195	195	165	170
Aa4273	13	431	431	135	137	203	219	192	192	412	412	250	250	230	236	230	230	205	213	191	197	195	195	170	170
Aa4274	13	427	431	125	125	221	231	182	194	412	412	253	253	236	314	230	230	213	213	191	195	195	201	170	170
Aa4275	13	427	431	137	137	205	217	194	194	412	412	256	256	236	236	230	230	213	217	191	207	195	201	170	170
Aa4276	13	427	429	137	151	211	231	180	180	412	412	256	271	236	258	230	230	209	213	195	207	195	198	170	170
Aa4277	13	429	431	137	137	201	219	182	184	412	412	256	256	236	248	230	230	209	213	189	207	198	198	170	170
Aa4278	13	431	431	137	167	187	219	182	194	412	412	250	256	236	238	230	230	213	213	197	207	201	201	175	175
Aa4279	13	427	431	137	137	217	221	182	194	412	412	253	253	236	322	230	230	201	205	191	191	195	201	180	190
Aa4280	13	431	431	125	137	199	217	182	194	412	412	250	256	230	236			201	213	191	191	195	201	165	165
Aa4281	13	429	431	125	125	201	205	184	206	412	412	253	256	242	260	235	235	213	213	205	205	198	198	170	170
Aa4282	13	431	431	125	125	195	213	182	182	412	412	253	256	238	238	235	235	213	213	191	207	195	201	170	170
Aa4283	13	429	431	137	137	213	221	194	206	412	412	256	256	236	264	225	225	213	213	195	195	195	195	165	165
Aa4284	13	431	431	139	139	187	201	194	194	412	418	250	256	236	264			205	213	191	195	195	201	175	175
Aa4285	13	427	431			213	217	182	182	412	412	253	253	230	230			205	205	191	197	195	195	170	170
Aa4286	13	431	431	143	143	217	231	182	192	412	412	256	256	240	264	230	235	217	217	207	207	195	195	175	175
Aa4287	13	427	429	135	135	213	215	182	182	412	412	250	250	236	236	230	230	201	213	191	197	198	201	175	175
Aa4288	13	429	431	125	137	201	205			412	418	253	253	264	322	230	230	209	213	191	209	195	198	170	175
Aa4289	13	431	431			217	217	184	206	412	412	256	256	248	248			201	213	195	195	195	201	170	170
Aa4290	13	431	431	133	133	217	217	182	182	412	412	256	256	236	238			213	213	191	195	195	198	170	170
Aa4291	13	427	427	143	143	205	205	182	182	412	412	256	256	236	236	230	230	213	213	191	197	195	195	190	190
Aa4292	13	427	427	137	137	213	219	182	206	412	412	250	256	236	238	230	230	213	213	191	207	195	204	165	170
Aa4293	13	431	431	125	165	213	213	182	182	412	412	253	256	236	238	230	230	209	213	197	207	195	198	170	175
Aa4294	13	429	431	125	133	209	209	208	208	412	412	250	256	236	236	230	235	205	209	195	195	195	201	170	170
Aa4295	13	429	429	135	137	217	231	182	204	412	412	244	244	236	236	230	230	201	213	191	195	195	195	175	190
Aa4296	13	431	431	139	165	205	211	186	186	412	412	256	256	236	238	230	230	213	213	191	191	198	198	170	170
Aa4297	13	429	431			215	219	192	192	412	412	250	250	230	264	230	230	205	213	191	207	195	195	170	170
Aa4298	13	427	431	135	139	199	213	182	184	412	412	241	256	238	260	230	230	209	209	207	207	195	195	170	175
Aa4299	13	427	431			201	213	182	186	412	412	256	256	236	264	230	230	213	213	207	209	198	198	170	170
Aa4300	13	427	431	143	143	201	209	192	206	412	412	250	253	236	236	230	235	201	213	191	197	195	201	170	175
Aa4301	13	427	427	125	137	207	221	182	194	412	412	253	256	236	236	230	230	205	213	191	191	195	198	175	190
Aa4302	13	431	431	157	157	213	213	170	184	412	418	256	256	236	236	230	230	213	213	191	207	195	198	175	190
Aa4303	13	431	431	137	137	205	205	194	194	412	412	256	256	236	236	230	230	201	213	191	195	198	201	170	190
Aa4304	13	431	431	137	137	213	217	194	194	412	412	253	253	236	264	230	230	213	213	191	191	195	198	165	170

Aa4305	13	431	431	143	143	217	221	192	192	412	412	256	256	236	320	230	235	201	213	195	195	195	195	170	170
Aa4306	13	431	431	135	137	195	213	182	182	412	412	253	256	238	264	230	230	213	213	191	207	195	198	175	175
Aa4307	13	429	429	137	137	207	207	194	204	412	412	241	241	236	236	235	235	209	213	207	207	195	198	170	170
Aa4308	13	431	431	125	137	219	219	182	182	412	412	250	256	236	236	230	230	205	213	207	207	195	195	165	165
Aa4309	13	431	431	125	137	199	231	182	184	412	418	241	241	238	264	230	230	209	213	191	191	195	198	165	170
Aa4310	13	431	431	125	135	201	201	182	182	412	412	250	250	236	260	230	235	209	213	191	207	198	204	170	170
Aa4311	13	427	429	139	139	205	231	192	194	412	412	250	253	230	264	230	230	193	205	207	207	195	195	170	170
Aa4312	13	427	427	169	169	191	191	194	194	412	412	253	256	264	264	230	235	213	213	191	191	195	198	175	175
Aa4313	13	431	431	139	139	217	225	184	184	412	412	253	256			230	230	213	213	207	207	195	198	175	175
Aa4314	13	429	429	135	135	199	213	194	194	412	412	253	256	236	236	230	230	213	213	197	207	195	195	165	175
Aa4315	13	427	429	137	137	201	227	182	182	412	412	250	250	236	236	230	230	213	213	207	207	195	201	190	190
Aa4316	13	431	431	137	141	205	219	192	192	412	418	256	256	230	238	230	230	213	213	195	207	195	195	170	175
Aa4317	13	427	429	133	153	191	225	182	182	412	412	250	256	238	238	235	235	205	213	191	207	195	195	170	170
Aa4318	13	427	431	135	135	201	213	182	184	412	412	250	271	264	264	235	235	209	213	191	195	195	198	175	175
Aa4320	13	429	431	137	137	205	213	182	184	412	418	250	256	236	236	230	230	201	201	205	207	195	195	170	170
Aa4321	13	427	427	133	133	205	217	182	188	412	412	253	262	264	322	230	230	213	213	191	209	201	201	175	175
Aa4322	13	431	431	137	137	203	205	186	186	412	412	253	262	236	264			213	213	205	209	195	195	170	175
Aa4323	13	429	429	169	169	203	207	182	194	412	412	253	256	236	274	235	235	205	209	195	207	195	195	165	170
Aa4324	13	429	429	165	165	203	231	194	194	412	412	250	253	236	276	230	230	209	209	207	207	195	195	170	175
Aa4325	13	431	431	125	143	201	201	194	194	412	412	250	256	236	236			209	209	207	207	195	195	170	170
Aa4326	13	431	431	125	143	211	217	194	194	412	412	256	256	236	264	230	230	209	209	191	197	195	201	170	170
Aa4327	13	427	427	137	137	201	213	186	186	412	412	250	256	230	322	230	230	209	209	197	207	195	198	170	170
Aa4328	13	427	431	139	139	205	207	182	182	412	412	250	256	236	236	230	235	201	201	191	191	195	195	175	190
Aa4329	13	429	431	125	143	213	221	194	194	412	412	253	256	236	260	230	230	213	213	191	191	201	201	170	175
Aa4330	13	427	431	135	135	191	213	188	192	412	412	250	250	236	238	230	230	213	213	191	207	195	195	175	175
Aa4331	13	431	431	125	125	217	227	182	182	412	412	253	253	236	236	230	230	213	213	207	207	192	195	165	170
Aa4332	13	429	433	137	137	217	217	186	186	412	418	253	253	322	322	230	230	213	213	207	209	195	198	165	175
Aa4333	13	429	431	135	135	197	205	182	194	412	412	256	256	236	260			209	213	207	209	195	198	175	175
Aa4334	13	429	429	155	155	195	221	182	182	412	412	256	256	260	322			213	217	189	207	195	201	190	190
Aa4335	13	429	431			207	211	182	206	412	412	256	256	238	270			205	205	207	207	195	195	170	175
Aa4336	13	429	431	137	137	219	219	182	194	412	412	247	256	236	238	235	235	209	209	197	207	201	201	170	170
Aa4337	13	427	429	139	139	217	217	184	198	412	412	250	253	228	264	230	230	201	209	205	205	195	195	175	175
Aa4338	13	431	431	125	139	211	211	192	192	412	412	256	256	230	236	235	235	201	213	191	191	195	195	175	175
Aa4339	13	431	431	157	157	213	217	192	198	412	412	253	253	236	236	230	230	213	213	207	207	195	195	170	170
Aa4340	13	431	431	155	167	201	205	182	182	412	412	250	256	236	236	230	235	213	213	191	191	195	198	190	190
Aa4341	13	431	431	137	139	213	231	182	182	412	412	256	256	238	264	230	230	205	213	191	191	195	198	190	190
Aa4342	13	427	431	139	139	205	217	194	194	412	412			236	236	230	235	213	213	207	207	198	198	165	170
Aa4343	13	429	431	125	137	201	205	182	194	412	412	250	253	260	264	230	230	213	213	191	207	195	195	170	170
Aa4344	13	431	431	137	137	203	217	182	206	412	412	256	256	236	236	230	230	209	213	191	191	195	195	175	175
Aa4347	14	431	431	133	137	201	231	182	184	412	412	256	271	232	236	230	235	201	213	207	209	195	195	170	175
Aa4348	14	431	431	137	165	213	229	182	194	412	412	253	253	248	280	230	230	205	213	195	195	195	198	170	170
Aa4349	14	429	431	137	137	205	231	184	194	412	412	253	253	236	236	230	230	209	213	207	207	198	204	170	170
Aa4350	14	429	431	125	137	201	221	194	194	412	412	244	253	230	280	225	230	209	209	191	207	195	195	170	170
Aa4351	14	431	431	141	167	201	205	192	192	412	412	241	241	236	236	230	230	213	213	191	207	198	201	165	165
Aa4352	14	427	427	137	137	201	231	182	182	412	412	241	262	228	264	230	230	205	213	191	191	195	195	165	170
Aa4353	14	427	431	125	137	201	207	182	182	412	412	250	256	236	236	230	230	209	213	197	197	195	198	190	190
Aa4354	14	427	427	137	139	213	217	182	182	412	412	253	256	236	236	230	230	205	213	191	191	195	198	190	190
Aa4355	14	429	429	125	137	203	217	182	206	412	412	250	271	264	264	230	230	209	209	207	207	195	198	170	170
Aa4356	14	429	431	159	159	201	217	182	206	412	412	250	250	232	248	230	235	209	213	191	191	195	198	190	190
Aa4357	14	429	431	133	137	203	219			412	412	256	256	236	248	230	235	213	213	189	209	195	198	170	170
Aa4358	14	429	429			217	227	182	192	412	412	256	256	238	238	235	235	205	213	191	191	195	195	170	170
Aa4359	14	427	429	125	141	201	201	182	182	412	412	259	259	248	248	230	235	205	205	207	207	195	198	165	165
Aa4360	14	431	431	135	137	207	211	186	192	412	412	256	256	236	236	230	230	209	213	191	191	195	201	170	170
Aa4361	14	427	431	137	137	211	213	182	194	412	412	250	250	236	236	230	230	213	213	191	191	198	198	170	175
Aa4362	14	429	431	125	137	201	205	184	194	412	412	250	253	236	236	230	230	213	217	207	207	195	195	175	175
Aa4363	14	431	431	135	137	187	201	182	192	412	412	253	259	230	260	230	230	213	213	191	207	195	198	170	190
Aa4364	14	431	431	125	133	201	213	182	194	412	418	250	250	236	236	230	230	213	213	207	207	198	201	170	170
Aa4365	14	427	427			201	205	184	184	412	412	250	256	236	236	230	230	213	213	197	197	195	198	170	170
Aa4366	14	427	431	137	137	187	195	182	182	412	412	253	256	236	238	230	235	213	213	191	191	198	201	175	190
Aa4367	14	427	427	137	137	213	231	188	194	412	418	253	253	230	238			209	213	193	207	195	195	170	170
Aa4368	14	427	431	159	159	187	203			412	412	253	253	248	248	230	230	205	209	191	193	195	195	190	190
Aa4369	14	429	431	125	125	217	217	182	196	412	412	253	253	236	236	230	235	213	213	197	207	195	195	165	190
Aa4370	14	431	431	125	139	199	199	182	184	412	412	250	253	230	230	230	230	209	213	197	207	195	201	165	165
Aa4371	14	431	431	133	133	213	213	182	182	412	412	256	256	230	320	230	230	209	213	207	209	195	201	170	170
Aa4372	14	429	429			201	231	184	202	412	418	250	250	238	320	230	230	193	213	207	209	195	198	165	170
Aa4373	14	431	431	125	137	195	205	184	194	412	412	244	244	236	236	230	230	201	205	191	197	195	198	170	170
Aa4374	14	427	429	137	137	217	217	194	194	412	418	250	253	236	236	230	230	205	213	195	195	195	198	170	175
Aa4375	14	427	431	137	139	213	217	182	182	412	412	253	253	230	236	230	230	209	213	207	207	198	198	170	170
Aa4376	14	429	429	137	137	201	231	182	182	412	412	250	250	264	264	230	230	205	213	197	207	195	201	175	175
Aa4377	14	431	431	139	143	205	219	182	184	412	412			236	236	230	230	201	213	195	207	195	198	175	175
Aa4378	14	429	429	125	139	187	211	182	206	412	412	250	256	238	238	230	230	209	213	191	207	195	201	175	175
Aa4379	14	427	427	125	125	205	205	194	208	412	412	250	274	236	238	230	230	205	213	191	191	195	201	170	170
Aa4380	14	427	431	125	125	201	211	182	202	412	412	250	256	224	236	230	230	213	213	207	207	195	198	170	175
Aa4381	14	429	431	155	155	213	213	178	192	412	412	241	256	236	236	230	230	209	213	207	207	195	201	170	175
Aa4382	14	431	431	125	143	207	221	186	186	412	412	253	253	236	322	230	230	213	213	207	207	198	201	170	170
Aa4383	14	431	431	137	137	205	223	170	182	412	412	250	256	236	236	230	230	213	213	195	205	195	198	165	170
Aa4384	14	427	431	137	137	201	221	180	182	412	412	256	256	236	236	230	230	209	213	207	207	195	198	170	170
Aa4385	14	431	431	125	137	201	213	182	182	412	412	250	250	238	248			201	201	191	207	195	195	175	175

Aa4386	14	429	431	137	137	201	217	182	182	412	412	250	253	238	240	225	230	213	213	191	191	195	198	190	190
Aa4387	14	429	431	137	137	219	223	182	194	412	412	256	256	236	264	225	225	205	213	195	207	195	198	170	175
Aa4388	14	429	431	139	139	221	221	184	192	412	412	256	256	232	238	230	230	205	213	191	207	195	195	165	170
Aa4389	14	431	431	125	125	195	231	206	206	412	418	250	256	232	244	230	230	205	205	207	207	195	201	170	175
Aa4390	14	431	431	137	137	187	223	194	194	412	412	253	253	228	228	225	230	205	213	207	207	195	195	175	190
Aa4391	14	431	431	125	137	213	213	182	182	412	412	250	256	226	238	230	230	213	213			195	198	165	170
Aa4392	14	429	431	135	137	217	225	192	192	412	412	250	250	236	236	230	230	201	213	191	195	195	195	165	170
Aa4393	14	429	429	137	137	213	217	182	206	412	412	250	250	236	236	230	235	209	213	197	207	195	198	165	165
Aa4394	14	429	429	137	165	187	199	184	194	412	418	256	256	236	240	235	235	201	213	207	209	195	195	170	170
Aa4395	14	427	431	137	137	205	221	182	194	412	412	253	256	236	240	230	230	209	213	197	209	195	195	170	170
Aa4396	14	429	431	139	139	201	213	182	194	412	412	253	271	264	264	230	230	201	205	207	207	195	198	170	170
Aa4397	14	431	431	137	137	213	231	182	194	412	412	253	253	260	260	225	225	205	213	195	207	195	201	175	175
Aa4398	14	431	431	135	135	201	205	182	182	412	412	253	256	236	236	230	235	205	213	197	197	195	198	165	170
Aa4399	14	431	431	137	137	201	219	182	182	412	412	253	256	236	236	225	230	205	209	197	197	195	195	165	170
Aa4400	14	431	431	125	125	201	205	182	182	412	412	244	250	248	248	235	235	213	213	207	207	195	201	170	170
Aa4401	14	429	431	137	137	197	217	188	188	412	412	250	250	230	264	230	230	209	213	191	191	201	201	175	175
Aa4402	14	431	431	125	125	201	231	182	194	412	412	253	253	236	236	235	235	201	213	207	207	195	198	170	170
Aa4403	14	429	429	155	165	213	217	182	182	412	412	250	256	236	238	230	230	205	213	207	209	195	198	170	170
Aa4404	14	429	429	137	137	205	231	184	184	412	412	250	256	268	314	230	230	213	213	207	207	195	198	170	170
Aa4405	14	427	429	137	153	203	203	182	192	412	412	253	256	236	236	235	235	209	213	207	207	195	195	165	165
Aa4406	14	431	431			213	217	192	192	412	412	256	271	236	236	230	230	205	213	191	191	195	195	170	170
Aa4407	14	429	429	137	141	201	213	188	188	412	412	241	250	236	280	235	235	201	213	197	207	195	198	170	175
Aa4408	14	427	431	165	165	201	211			412	412	250	250	236	236	230	235	209	213	191	191	195	195	170	170
Aa4409	14	431	431	125	157	201	211	182	184	412	412	253	256	240	264	230	230	213	213	197	207	195	195	170	170
Aa4410	14	427	427	137	143	219	231	182	192	412	412	253	256	238	238	230	230	213	213	197	197	195	198	165	170
Aa4411	14	427	431	125	125	213	215	184	184	412	418	256	256	236	320	230	230	205	213	207	207	195	195	165	165
Aa4412	14	427	429	143	143	201	217	182	182	412	412	253	256	236	236	230	230	205	209	195	207	198	201	165	175
Aa4413	14	431	431	137	137	201	201	192	194	412	412	250	250	236	248	230	230	213	213	191	197	195	198	185	185
Aa4414	14	427	431			201	211	184	184	412	412	253	253	238	238	230	230	213	213	207	207	195	198	175	175
Aa4415	14	427	431	135	165	223	231	182	182	412	412	259	259	236	240	230	230	201	213	191	209	195	198	170	175
Aa4416	14	429	431	133	137	213	215	184	208	412	412	250	253	236	236	230	230	205	213	191	207	198	198	190	190
Aa4417	14	431	431	137	137	217	217	182	194	412	412			236	322	230	230	213	213	191	207	195	201	170	170
Aa4418	14	431	431	125	137	205	205	182	202	412	412	250	256	236	238	230	230	213	213	191	191	195	195	190	190
Aa4419	14	429	431	137	137	217	223	186	194	412	412	256	256	236	236	230	230	213	213	195	195	195	195	165	165
Aa4420	14	431	431	125	125	205	241	192	194	412	412	250	256	236	248	230	230	193	209	191	193	198	201	170	170
Aa4421	14	429	431	125	125	213	221	192	206	412	412	250	250	236	264	230	230	201	213	191	207	195	198	165	165
Aa4422	14	431	431	137	137	207	207	182	194	412	412	253	253	248	258	230	235	209	213	191	207	195	198	170	175
Aa4423	14	427	427	139	139	191	221	192	196	412	412	244	256	268	268	230	235	201	213	197	197	195	201	165	165
Aa4424	14	427	429			213	217	182	182	412	412	250	253	228	236	230	235	213	213	207	209	195	195	170	175
Aa4425	14	429	431	137	167	221	231	186	192	412	412	250	250	236	236	230	230	205	213	197	209	195	195	170	170
Aa4426	14	429	431	137	141	205	205	198	206	412	412	256	256	228	240	230	230	209	213	191	191	195	201	170	175
Aa4427	14	431	431	137	165	231	231	192	194	412	412	256	256	236	264	235	235	209	213	207	207	198	198	175	175
Aa4428	14	431	431	137	137	213	231	182	194	412	412	250	271	230	230	230	230	205	209	191	207	198	201	165	190
Aa4429	14	431	433	155	155	199	201	192	194	412	412	253	256	264	264	230	230	201	201	191	207	198	201	170	170
Aa4430	14	431	431	125	137	217	217	182	182	412	412	250	253	238	264	230	230	205	217	191	191	195	198	175	175
Aa4431	14	427	427	137	141	217	221	182	182	412	412	250	250	236	242			205	213	197	207	195	198	170	175
Aa4432	14	429	431	125	165	201	217	194	194	412	412	250	253	260	322	230	230	213	213	207	207	198	198	170	190
Aa4433	14	431	431	137	137	201	205	192	194	412	412	250	256	230	260	230	230	209	213	191	207	198	201	165	175
Aa4434	14	429	431	137	137	205	243	194	194	412	412	253	256	244	316	235	235	205	213	207	207	195	201	170	175
Aa4435	14	427	429	143	167	201	213	182	182	412	418	250	250	236	240	230	230	205	213	207	209	195	195	165	170
Aa4436	14	425	429	137	139	201	205	194	194	412	412	250	250	236	238	230	230	213	213	205	207	195	201	170	170
Aa4437	14	431	431	125	139	207	217	182	184	412	412	253	253	236	236	230	230	205	213	191	191	195	204	175	185
Aa4438	14	429	431	133	139	213	217	192	194	412	412	256	256	280	280	230	230	201	209	207	207	195	195	170	170
Aa4439	14	427	429	169	169	211	211	182	186	412	412	250	253	264	264	230	230	205	213	207	207	195	195	165	190
Aa4440	14	427	427	125	125	201	213	182	194	412	412	253	256	240	248	230	230	205	213	191	193	195	198	170	170
Aa4441	14	427	431	153	153	201	211			412	412	256	256	236	238	230	230	213	213	207	209	195	198	175	190
Aa4442	14	429	431	133	133	213	217	192	192	412	412	253	253	238	264	230	230	209	213	191	207	195	198	170	190
Aa4443	14	429	431	125	137	187	201	182	182	412	412	250	250	236	260	230	230	205	213	191	207	195	198	170	175
Aa4444	14	427	427	137	143	213	213	182	182	412	412	241	256	236	314	230	230	193	213	191	205	195	195	175	190
Aa4445	14	431	431	137	137	231	231	184	194	412	412	250	256	240	248	230	230	205	209	191	191	195	195	165	175
Aa4446	14	427	431	125	125	191	213	192	206	412	412	241	256	236	240	230	235	213	213	191	209	195	201	170	175
